# Interventions designed to improve financial capability: A systematic review

**DOI:** 10.1002/cl2.1225

**Published:** 2022-03-14

**Authors:** Julie Birkenmaier, Brandy Maynard, Youngmi Kim

**Affiliations:** ^1^ Saint Louis University School of Social Work St. Louis Missouri USA; ^2^ School of Social Work Virginia Commonwealth University Richmond Virginia USA

## Abstract

**Background:**

There is growing recognition that people need stronger financial capability to avoid and recover from financial difficulties and poverty. Researchers are testing financial capability interventions with adults, children, immigrant populations and other groups, but little is known about the effectiveness of financial capability interventions on financial behaviour and financial outcomes.

**Objectives:**

The purpose of this review is to inform practice and policy by examining and synthesizing evidence of the effects of interventions designed to improve financial capability. Financial capability interventions combine financial education and financial products and/or services. The research questions are: (a) What are the effects of interventions designed to improve financial capability on financial behaviour and financial outcomes? and (b) Does study(design), intervention (dosage, duration, type) or sample (age) characteristics relate to the magnitude of effect size?

**Methods:**

We conducted two identical rounds of electronic searches for two different time periods. In Round 1 searched for studies through May, 2017 and Round 2 searched from May, 2017 through May, 2020. For both rounds, we identified and retrieved both published and unpublished studies, including conference proceedings, through a comprehensive search that included multiple electronic databases, grey literature sources, organizational websites, government websites and reference lists of reviews and relevant studies. We also conducted forward citation searching using Google Scholar to search for studies citing the included studies. We also conducted a search on Google using key terms. We hand searched the table of contents of selected journals to identify potentially eligible reports not properly indexed. Finally, experts who were study or sub‐study authors of prior studies were contacted in an attempt to obtain unpublished studies, studies in process and published studies missed in the database search.

**Selection Criteria:**

To be eligible for this review, the intervention must have included a financial education component and a financial product or service. Studies must have also been conducted in any of the 35‐member countries of the OECD, and included a financial behaviour or financial outcome. To meet the criteria for delivering financial education, interventions must have delivered information about: (1) a variety of general financial concepts and behaviours, or advice about financial behaviours); (2) a specific financial topic; (3) a specific product; and/or (4) a specific service. To meet the criteria for access to a financial product or service, interventions must have facilitated access to one or more of the following: (1) a child development account; (2) a retirement account through an employer; (3) a ‘second chance’ checking account; (4) a matched savings account; (5) a financial service, such as financial counselling or coaching; (6) a bank account; (7) an investment vehicle; or (8) a home mortgage loan product.

**Data Collection and Analysis:**

Electronic searches of bibliographic databases and searches of other sources identified a total of 35,484 hits. Titles and abstracts were screened for relevance and 35,071 were excluded as duplicates or deemed inappropriate. The full text of the remaining 416 potential studies was reviewed and screened for eligibility by two independent coders. We excluded 353 reports that were deemed ineligible and included 63 reports that met inclusion criteria. Of the 63, 15 reports were deemed duplicates or summary reports. Of the remaining 48 reports, 24 were unique studies (using unique samples) that were included in this review. Six of those 24 studies were large longitudinal studies that presented unique analyses (using different time points, subsamples, and/or outcomes). Thus, we extracted data from 48 reports, reporting data and analyses from 24 unique studies. At least two review authors who were not study authors independently assessed risk of bias in all included studies using the Cochrane Collaboration's risk of bias tool.

**Results:**

The review summarizes evidence from 63 reports from 24 unique studies, which included 17 randomized controlled trials and 7 quasi‐experimental designs. In addition, 17 duplicate or summary reports were also located. This review identified several different types of previously evaluated financial capability interventions. Unfortunately, few interventions that were evaluated by more than one study measured the same or similar outcomes, thus there were not a sufficient number of studies of any of the included intervention types that could be pooled to conduct a meta‐analysis. Therefore, evidence is sparse about whether participants’ financial behaviours and/or financial outcomes are improved. While the majority of the studies used random assignment (72%), many of the studies had some important methodological weakness.

**Authors’ Conclusions:**

There is a lack of strong evidence about the effectiveness of financial capability intervention. Better evidence is needed about the effectiveness of financial capability interventions to guide practitioners.

## 1 PLAIN LANGUAGE SUMMARY

### 1.1 Little rigorous evidence on interventions combining financial education with financial products and services from mainstream financial institutions

There is no clear evidence that financial capability interventions, which include financial education linked to a mainstream financial product or service, improve financial behaviours or financial outcomes.

### 1.2 What is this review about?

The growth in individual responsibility for one's finances dovetailing with the growth in financial products and services, including those in the alternative financial services sector, has resulted in higher financial risk. People need stronger financial capability to avoid and recover from financial difficulties.

Financial capability, or the ability to use knowledge to demonstrate desirable behaviours towards financial well‐being, requires knowledge, access and ability to use a financial product or service.

This systematic review assesses the state of research on interventions that combined financial education and a mainstream financial product or service (‘financial capability interventions’). It examines the financial behaviours and financial intervention outcomes.

**What is the aim of this review?**

This Campbell systematic review examines the effects of interventions that combine financial education and mainstream financial products and services. The review summarizes evidence from 63 reports from 24 unique studies.

### 1.3 What are the main findings of this review?

#### 1.3.1 What studies are included?

For this review, the intervention must include financial education and a financial product or service. Studies that described interventions that provided only financial education, or financial education services (e.g., mentoring), or only facilitated financial access, were excluded.

This review includes studies that evaluate the effects of financial capability interventions compared to a group that received nothing, treatment as usual, or different treatment. A total of 63 reports were identified, with another 17 duplicate/summary reports. Therefore, 48 reports of 24 unique studies were included.

Six of the 24 are large longitudinal studies that are reported on in 28 sub‐studies that use various time points post‐treatment and/or examined different outcomes from the same or different samples.

The studies spanned the years 2004–2020 and were all conducted in the USA. The majority of the studies were randomized control trials.

### 1.4 What are the main findings of this review?

Financial capability interventions include financial education and access to a financial product or service from a mainstream financial institution.

Data were collected on financial behaviour and financial outcomes of the study participants using unstandardized instruments and included self‐reported and administrative data.

Behaviour changes included bank or retirement account opening, asset purchase, savings rate, budgeting and retirement savings rate. Financial outcomes included savings amount, credit score, debt amount, asset value and retirement savings amount.

This review identifies several types of previously evaluated financial capability interventions. Few interventions that were evaluated by more than one study measured the same or similar outcomes, thus there was an insufficient number of studies of any of the included intervention types that could be pooled to conduct a meta‐analysis. Therefore, evidence is sparse about whether participants' financial behaviours and/or financial outcomes are improved.

Many studies had important methodological weakness, and a high or unclear risk of bias.

### 1.5 What do the findings of this review mean?

There is a lack of strong evidence about the effectiveness of financial capability interventions. Better evidence is needed about the effectiveness of financial capability interventions to guide practitioners. Policy actors that seek to facilitate increased financial capability through the interventions included in this review need a stronger evidence foundation.

Additional research on financial capability interventions using protocols, strong and transparent methodology, manualised interventions, common outcomes and more complete reporting is needed.

### 1.6 How up‐to‐date is this review?

The review authors searched for studies up to May 2020.

## 2 BACKGROUND

### 2.1 The problem, condition or issue

#### 2.1.1 Financial capability

Financial capability, defined as ‘a consumer's ability to apply financial knowledge and perform desirable financial behaviours to achieve financial well‐being’ (Xiao & O'Neill, 2014), is gaining attention in practice and public policy. Financial capability requires both knowledge and access to and the ability to use a financial product or service. Today's financial context is more complicated than in previous generations in that it offers more types of financial products and services from which to choose, and people have greater responsibility for making some financial decisions with long‐term ramifications compared to previous generations that had, for example, increased access to pensions. The growth in individual responsibility dovetailing with the growth in financial products and services, including those in the Alternative Financial Services sector, has resulted in higher risk than ever for making financial decisions. These risks are most significant for lower‐income households, who have less income, fewer emergency savings (Collins & Gjertson, 2013), and less financial wealth (Killewald et al., 2017), than families with more income and wealth to absorb financial mistakes and economic downturns. These households also have less access to financial products and services from mainstream providers (Hegerty, 2016, 2019), such as bank accounts, affordable consumer loans, and employer‐provided retirement accounts, and lower levels of financial literacy to guide them (Lusardi & Mitchell, 2014). As a result of these trends and recent global financial crisis and subsequent growth in income and wealth inequality to levels unprecedented in recent US history, there is growing recognition that people need stronger financial capability to avoid and recover from financial difficulties and poverty (Miller et al., 2014; Mitchell & Lusardi, 2015).

### 2.2 The intervention

There is growing academic and public policy interest in helping people gain financial capability. Researchers are testing financial capability interventions with adults, children, immigrant populations, and other groups (Batty et al., 2015; Curley & Robertson, 2017; Huang et al., 2013; Theodos et al., 2015). These interventions use various methods to increase financial education combined with financial products and services. The interventions differ in their methods of financial education and financial products and services, and their method for combining them, but they share this coordinated combination. For example, interventions to help parents learn to save money include financial education and access to a college savings account for their child (Huang et al., 2013). Policymakers are showing increased interest in these interventions, and implementing new policies designed to increase financial capability. For example, the states of Maine and Nevada have started state‐wide financial capability and asset building programs (Clancy et al., 2015). Countries are creating national strategies on financial capability, such as the countries within the United Kingdom, which set out a clear description of the problem and define clear goals for specific populations and geographic areas (Bagwell et al., 2014; Kempson, 2009). As outcomes, studies measure financial behaviours, such as setting aside savings as emergency or short‐term savings (Azurdia & Freedman, 2016; Collins & Urban, 2015; Huang et al., 2013; Skimmyhorn, 2012; Theodos et al., 2015), engaging in financial management (e.g., keeping records of expenses and income, paying bills on time, and using a budget) (Theodos et al., 2015), improving credit (Birkenmaier et al., 2014a; Theodos et al., 2015), participating in retirement savings plan (Duflo et al., 2006) and saving for an asset, such as children's college education (Han et al., 2009; Huang et al., 2013; Sherraden et al., 2011). Some researchers have also studied financial knowledge (Azurdia & Freedman, 2016; Han et al., 2009; Theodos et al., 2015) and financial mind‐set (i.e., attitudes, motivation, and decision‐making) (Skimmyhorn, 2012; Theodos et al., 2015). These outcomes all have the potential to impact a person's financial behaviours, which in turn impacts financial well‐being, or the ability of a household to ‘fully meet current and ongoing financial obligations, …feel secure in their financial future, and…make choices that allow them to enjoy life’ (Consumer Financial Protection Bureau [CFPB], 2015). Other financial interventions also combine financial education and financial products or services and are considered in this review. These interventions may be known as financial counselling and coaching, and combine financial education and access to financial products and services. Financial mentoring and financial therapy are interventions that do not routinely combine these two, and are excluded from this study.

### 2.3 How the intervention might work

There is a growing awareness that financial knowledge, while necessary for optimal financial choices and behaviours, is insufficient by itself in today's world (Austin & Arnott‐Hill, 2014; Fernandes et al., 2014; Hastings et al., 2013; Miller et al., 2014; Mitchell & Lusardi, 2015). Results of meta‐analysis studies focused on financial education efforts alone suggest that by itself, financial education has weak effects on financial behaviour (Fernandes et al., 2014; Kaiser & Menkhoff, 2019; Miller et al., 2014). Fernandes et al. (2014) found that interventions to improve financial knowledge and skills explain only 0.1% of the variance in financial behaviours studied, with weaker effects in low‐income samples. A second meta‐analysis found that the impacts of financial education are highly heterogeneous (Kaiser & Menkhoff, 2016). Miller (2014) found that the combination of financial knowledge and skills has a positive impact on some behaviours and outcomes (savings, financial skills), but not in all studied (debt repayment), and had no significant overall effect size findings. Kaiser and Menkhoff (2019) found that financial education delivered in schools for children and youth had an effect of 0.07 standard deviation units on financial behaviours.

Thus, while financial education may lead to financial knowledge, the relationship between knowledge and behaviour appears weak. However, research suggests that access to financial products and services from mainstream financial institutions (e.g., bank accounts and retirement accounts) is significantly associated with financial behaviour (Birkenmaier & Fu, 2020), and financial behaviour is associated with financial well‐being indirectly through its relationship with one's objective financial situation. In other words, access to financial products and services is associated with financial behaviour, and financial behaviour may impact the objective financial situation, which may in turn impact financial well‐being (Walker et al., 2018).

**Figure 1:**
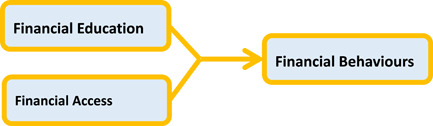
Framework of financial capability

Rather than focusing solely on financial education or on financial products and services, a focus on the combination of *financial knowledge and skills*, and *access to appropriate financial products and services* (Sherraden, 2013), or financial capability, has demonstrated promise to result in financial behaviours and outcomes that facilitate financial well‐being (Collins & Urban, 2015; Curley & Robertson, 2017; Huang et al., 2013; Theodos et al., 2015). This combination is grounded in Sen (1999) and Nussbaum's (2007) theoretical work on capability, which postulates that people's choices reflect both their knowledge and their real opportunities within their lived environment. Capability incorporates people's internal capabilities (abilities, knowledge, and skills) with external capabilities (e.g., the range of opportunities available through products, services, and institutions). Internal and external capabilities interact to further develop one's internal capabilities (Nussbaum, 2011, p. 21). Applying these concepts to financial capability means focusing on the financial decisions people make based on their innate ability, knowledge, skills, as well as the opportunities afforded them through their environment. Their innate ability to demonstrate financial behaviours is a result of the interaction of their internal and external capabilities, and growth of their internal capability through such interaction (Sherraden, 2013). To improve one's financial behaviours, a focus on both internal capabilities through financial education and external capabilities through the financial products and services available to them is needed. As shown in Figure 1, the financial capability framework recognizes that both financial education and financial products and services are determinants of an individual's financial behaviours, and that the interaction of the two allows individuals to apply their knowledge and skills through their financial behaviours, such as the degree to which they save money for emergencies or retirement, pay bills on time, and invest in assets that appreciate in value (Huang et al., 2014; Peeters et al., 2018). The interventions that use both elements also utilize important elements identified by the World Bank as essential to better financial interventions. By combining knowledge and financial products and services, interventions are ‘targeted and relevant’, provided at a ‘teachable moment’ when the participant might apply the information in a real‐world setting presently or in the near future, and ‘give exposure to information over the longer‐term’ through the use of a product or service (Lundberg & Mulaj, 2014).

Interventions designed to increase financial capability are diverse, yet designed to both strengthen financial knowledge and access to appropriate financial products to impact behaviour. Financial knowledge interventions involve a diverse range of types, goals, and delivery models. Some financial knowledge interventions utilize a set curriculum (Birkenmaier et al., 2014a), while others are tailored to the client's situation (Huang et al., 2013), expressed goals and interest areas (Theodos et al., 2015), or are specific to a financial product or service (Azurdia & Freedman, 2016; Collins & Urban, 2015). Financial knowledge interventions may have the goal of increasing general financial knowledge (Han et al., 2009), financial knowledge specifically related to a particular client situation (Theodos et al., 2015), or financial knowledge about a particular type of product or service (Azurdia & Freedman, 2016; Collins & Urban, 2015). Delivery models include one‐on‐one financial education (Azurdia & Freedman, 2016; Theodos et al., 2015) online financial education (Collins & Urban, 2015), and classroom‐based financial education (Birkenmaier et al., 2014a). In interventions designed to improve financial capability, these financial knowledge elements are paired with a financial product or service element, such as debt or credit interventions for a particular client situation (Theodos et al., 2015), a child education savings account (Huang et al., 2013), savings and checking accounts at banks and credit unions (Birkenmaier et al., 2014a), retirement saving plans (Collins & Urban, 2015), matched savings accounts for asset development (Han et al., 2009), or emergency savings accounts (Azurdia & Freeman, 2016), among others. The range of financial capability behaviours impacted includes savings, investment, record keeping, and loan repayment behaviours (Miller et al., 2014).

An important difference among interventions, however, is whether the intervention includes only financial education, or whether financial education is linked to financial products and services onto which participants can act to better their financial situation with their newfound knowledge. Interventions that include only financial education and are not designed to increase participant access to appropriate financial products and services that allow them to act on their knowledge assume that knowledge alone can result in financial behaviour change. Indeed, prior reviews have found that financial education alone has weak effects on behaviour (Fernandes et al., 2014; Kaiser & Menkhoff, 2019).

Financial capability interventions to be included in this review combine financial education and financial products and services and thus capitalize on the interaction of the two elements to affect financial behaviour. Some of the interventions are created for one‐time studies, while others are incorporated into long‐term programs that are supported through government and/or private funding. The interventions reported on in this review that are part of large programs are the following:

a. Individual development accounts (IDAs). IDAs are special matched savings accounts designed to reduce poverty for adults. IDA programs, available across the US, require small, regular participant savings over time, and completion of financial education. Account proceeds, plus a matched amount from public or private funds, can be used to start a small business, pay for post‐secondary education or purchase a home. Starting in the late 1990s, US federal funding was available from the Assets for Independence Act (AFIA), but federal funding was discontinued in 2017. Since then, individual US states and private funding has been supporting the programs (Center for Social Development, 2020b).
b. Child development accounts (CDAs). CDAs are saving or investment accounts that are opened as early as birth, and are targeted to Post‐secondary education for youth, home ownership and enterprise development in adulthood. Programs typically provide an initial deposits and sometimes matching funds. They often include mandatory financial education for children and families. While there is currently no national funding in the US, local, state, and national governments worldwide are creating them with private and public funds (Center for Social Development, 2020a).
c. Home ownership education and counselling. The US Department of Housing and Urban Development (HUD) provides counselling through intermediary approved agencies to consumers regarding homeownership throughout the US HUD‐certified counsellors provide advice and resources for consumers who wish to purchase a home, in addition to counselling about foreclosure and homelessness. Federal funding from HUD reimburses agencies for the cost of the counselling for some types of services, while consumers are charged reasonable fees for other types (US Department of Housing and Urban Development, 2020).
d. Employer‐provided retirement accounts. Employer‐provided retirement funds are generally one of two types: a defined benefit plan (i.e., a pension) that provides a specific monthly benefit for life; or a defined contribution plan, that provides a vehicle for employees and employers (or both) to contribute to an account an amount indexed to an annual salary. Federal tax benefits through the Internal Revenue Service are provided to employers who provide retirement benefits to their employees (Internal Revenue Service, 2020).
e. Financial counselling and coaching. Financial counselling and coaching is provided by a wide variety of public, private and government sources without specific government sanction or regulation. Financial counselling provides tailored financial education and advice, while coaching provides tailored goal‐setting and motivation, along with education (CFPB, n.d.). While distinct from each other, often in practice, there is also overlap. Interventions often partner financial counselling and/or coaching with other components, such as homeownership education and counselling and IDAs.

### 2.4 Prior reviews

While several prior reviews discussed next contribute to our understanding of financial capability interventions, they have limitations. Only four prior reviews have been conducted on interventions intended to improve at least one aspect of financial capability.

Fernandes and colleagues (2014) examined effects of financial literacy and financial education interventions on financial behaviours. Although the review methods were not clearly reported and the inclusion criteria were not well defined, the authors appear to have included 168 studies (published and unpublished), with 90 of those being studies that manipulated financial literacy with some education intervention and the remaining being studies that measured financial literacy (correlational studies). Fifteen of the 90 intervention studies used a randomized design, and the others used a quasi‐experimental or pre–post design. They found that financial education interventions had weak effects on financial behaviour, especially in low‐income samples. They found that financial literacy explains only 0.1% of the variance in financial behaviours studied, with weaker effects in low‐income samples.

Miller and colleagues (2014) took a seemingly broader approach to their review, including any intervention that would impact financial knowledge, attitudes, and/or behaviours. They identified 188 studies via their search of one electronic database (Econlit), prior literature reviews, studies completed within the World Bank, and websites likely to include relevant studies. However, the authors reported that, ‘to reduce the number of studies to a manageable size…only articles from peer reviewed journals were included from Econlit, for the period January 2009 to September 2013’ (p. 7). Despite their seemingly broader inclusion criteria related to the interventions of interest, their meta‐analyses reported on outcomes of financial education interventions from a small number of studies on the following outcomes: savings behaviour (*n* = 6), retirement savings (*n* = 5), loan defaults (*n* = 4), and record keeping (*n* = 5). Findings indicate that financial education interventions had a positive and statistically significant mean effect on retirement savings (effect size [ES] = 0.08; 95% confidence interval [CI], 0.01, 0.16), but a null or negative and non‐statistically significant mean effect on savings (ES = 0.03; 95% CI, 0.00, 0.06), record keeping (ES = 0.04; 95% CI, 0.00, 0.09) and loan default (ES = −0.02; 95% CI, −0.06, 0.02). The authors did not provide any analysis or discussion regarding whether the interventions had any clinical or practical significance for any of the outcomes. It is also important to note that the authors did not provide any details about how they calculated effect sizes or even which effect size statistic they were reporting. Overall, the reporting of the eligibility criteria and methods used to search, select, and extract data from studies was not clear. Miller and colleagues may have included interventions that encompassed financial literacy, education and access to products and services, but the authors' inclusion criteria were not clear and they did not describe the types of interventions included, aside from referring to them as ‘financial education’.

Kaiser and Menkhoff (2016) also conducted a review of financial literacy and financial education interventions on financial behaviours. The authors included 126 impact evaluation studies (published and unpublished) that were designed to impact financial knowledge or behaviours and that report on financial literacy and/or financial behaviour outcomes. 44% of the included studies overlap with the Fernandes et al. (2014) study. 83% of the studies were of classroom financial education, 8% were of online financial education, 2% were individualized counselling interventions, and 7% were informational and behavioural nudges. Results suggest that financial education intervention impacts are less effective for low‐income clients and for those in low‐ and lower‐middle income countries. They also found that offering financial education at ‘teachable moments’ and with increasing educational intensity increases the success of financial education efforts.

Kaiser and Menkhoff (2019) conducted a systematic review and meta‐analysis of studies on school financial education programs for children and youth. They built on their existing data set from their 2016 study by using the same search strategy to collect published studies on financial education in school between October 2016 and Sept 2018. They included 37 studies, 16 of which were new from their previous review. 18 of the 37 studies were RCTs and the others were QEDs. Their meta‐analysis relied on 177 effect size estimates, 70 of which refer to treatment effects on measures for financial knowledge and 107 refer to treatment effects on a set of financial behaviours among students, including credit behaviour, budgeting behaviour, saving and retirement behaviour, and insurance behaviour. They found that financial education programs have, on average, 0.33 standard deviation impact on financial knowledge, which is similar to educational interventions in other domains. They also found a .07 standard deviation impact on financial behaviours among students. The results were robust irrespective of the meta‐analytic method used and when accounting for publication bias. The authors find that effect sizes are statistically significant and larger for primary schools as compared to secondary schools, and higher intensity of teaching increases effectiveness. The authors make specific program recommendations for teaching financial education in schools. It should be noted that the reporting of the eligibility criteria was unclear regarding definition of school (i.e., public or private, homeschool, pre‐school, etc.). Findings also did not include effect sizes on the various outcomes related to financial behaviour.

While these reviews provide some evidence related to effects of financial capability interventions, serious evidence gaps remain.

Fernandes et al. (2014) and Kasier and Mankhoff (2016, 2019) included only financial education or literacy efforts without access to a financial product or service. Therefore, the interventions were not designed to increase participant access to appropriate financial products or service that allow them to act on their knowledge. Similarly, Miller and colleagues (2014) appear to have focused on financial literacy interventions. If they included financial capability interventions, they made no distinction between the different types of interventions. In addition to this limitation, the Miller et al. (2014) study also has some shortcomings that limit its usefulness in policy formation. The review had an insufficient search strategy by using a limited number of databases, and therefore the review may have missed potentially relevant studies. The review does not sufficiently describe the types of evidence included in the review and does not assess the risk of bias in the included studies. Thus, these studies do not provide evidence about whether interventions that combine financial literacy with financial products and services are effective.

As interventions to improve financial capability move forward, more evidence is needed about the effects of interventions that combine financial education and financial products and services. It is important that practitioners, policy makers and stakeholders have access to synthesized evidence of the effects of approaches to improve financial capability to make informed decisions, rather than relying on results of individual studies. A systematic review could inform practice decisions by providing evidence about the components of a financial capability intervention that are essential to effect participant financial outcomes. Policy decisions about essential program design elements required for funding could also be guided by evidence.

## 3 OBJECTIVES

The purpose of this review is to inform practice and policy by examining and synthesizing evidence of the effects of interventions designed to improve financial capability. Financial capability interventions combine financial education and financial products and/or services. The following are the research questions for this review:

What are the effects of interventions designed to improve financial capability on financial behaviour and financial outcomes?

Does study (design), intervention (dosage, duration, type) or sample (age) characteristics relate to the magnitude of effect size?

## 4 METHODS

### 4.1 Criteria for considering studies for this review

#### 4.1.1 Types of interventions

While definitions, terms, and practices vary across studies, for purposes of this review, financial capability is defined as ‘a consumer's ability to apply financial knowledge and perform desirable financial behaviours to achieve financial well‐being’ (Xiao & O'Neill, 2014). Financial capability requires both knowledge and access to a financial product or service. Studies eligible for this review examined the effectiveness of interventions designed to improve financial capability that use a combination of financial education or information, and access to a financial product or service.

To be eligible for this review, the intervention must have included a financial education component and a financial product or service.

To meet the criteria for delivering financial education, interventions must have delivered information about: (1) a variety of general financial concepts and behaviours (such as using a formal education curriculum that covers the time value of money and the importance of keeping financial records), or advice about financial behaviours); (2) a specific financial topic (such as a formal or informal one‐time education session about savings, homeownership, or a consumer credit report); (3) a specific product (such as retirement savings accounts or savings accounts that can be used for emergency savings); and/or (4) a specific service, such as the value of pre‐purchase homeownership counselling to gain access to low‐cost financing. Information about a specific product or a specific company also met the criteria, such as in the case when employer‐based financial education is focused on educating their employees about their retirement plan, and options for investing within their plan. Financial education could have been high‐intensity, such as one‐on‐one delivery, one‐on‐one tailored delivery, or face‐to‐face classes, or low intensity, such as flyers, emails, texts, videos, or online delivery.

To meet the criteria for access to a financial product or service, interventions must have facilitated access to one or more of the following: (1) a CDA (used for post‐secondary education or training, or another type of asset purchased at age 18 or older); (2) a retirement account through an employer; (3) a ‘second chance’ checking account (for persons listed in a consumer reporting bureau after having insufficient funds for a check; (4) a matched savings account (to pay debts to build assets); (5) a financial service, such as financial counselling or coaching; (6) a bank account; or (7) an investment vehicle, such as a Saving Bond or Certificate of Deposit (CD); or (8) a home mortgage loan product. Facilitating access includes linking participants to products or services that are tailored towards the population of participants (such as financially vulnerable populations or employees eligible for employer‐provided retirement benefits). This linking could involve financial counselling or coaching, or occur through another process, such as tax filing. The interventions could also facilitate access by signing participants up for the product or service (such as a savings account), deliver services as part of the intervention (financial counselling or coaching), and/or provide on‐going interpersonal support to make it possible for participants to maintain access to a product or service.

Studies that used multicomponent interventions were included as long as two of the components were financial education or information and access to a financial product or service. Studies that described interventions that provided only financial education, or a myriad of other types of services related to financial education, such as literacy, mentoring, goal‐setting, and therapy, or only facilitated access to products and services were excluded from this review.

In our search, we found multiple reports of large longitudinal study projects that included different subsets of the large sample, different time periods (e.g., post‐test, 18 months post, 36 months post) or different outcomes reported. We also found duplicate reports of the primary studies, as well as summary reports that spanned the findings of multiple primary and secondary studies. We designated these as secondary reports and extracted data from all reports that were relevant to a particular study/primary report.

Studies conducted in non‐OECD countries were excluded for several reasons. First, this limitation assisted in maintaining a reasonable scope to the project, and could produce findings relevant to a large population in the US and other developed countries. Second, the financial system in the US and other developed countries have distinctive features not shared by financial systems in developing countries. The comparison of impact of financial capability interventions may not be justified across financial systems. Studies were also excluded that teach financial education only or only facilitate access to financial products and services.

#### 4.1.2 Types of participants

Financial and economic policies and practices can be quite different for high‐income compared to low‐ and middle‐income countries. Therefore, the focus of this review was on financial capability interventions in high‐income countries. To be included, studies must have been conducted with participants in any of the 35 member countries of the Organisation for Economic Co‐Operation and Development (OECD). Studies with participants of all ages were included.

#### 4.1.3 Types of outcome measures

##### Types of outcomes

Studies must have measured at least one of the following primary outcomes. These outcomes reflected financial behaviour:

###### Behaviour change

Behaviour change refers to changes in financial behaviours of participants, such as opening a savings or checking account, owning a retirement or College Savings account, active use of savings or checking accounts, increased frequency of savings, change from using predatory financial products to mainstream financial products, purchase of an asset, reviewing credit report, etc.

###### Financial outcomes

Financial outcomes refers to implications of behaviour change, such as higher fund balance in a savings or checking account, higher net worth, lower debt, and improved credit scores.

If studies measured one of the above primary outcomes, data was to be extracted on the adverse effects as a secondary outcome, such as a decrease in material well‐being as a result of saving for a child's college education.

Measurement of above outcomes could have been conducted using standardized or unstandardized instruments, and self‐ or other‐reported or researcher administered measures. Thus, the reviewers did not exclude measures based on the type of measure, but planned to pool effects based on type of measure used (e.g., observational measures pooled with observational measures). In the planned meta‐analysis, to be included, study authors must have reported enough information to calculate an effect size. If sufficient information to calculate an effect size was not provided, every effort was made to contact study authors and request the necessary information.

#### 4.1.4 Types of study designs

To mitigate threats to internal validity, studies must have used a prospective randomized controlled trial (RCT) or quasi‐experimental (QED) research design with parallel cohorts (the control/comparison group cannot consist of study dropouts). Studies using single‐group pre–post test design (SGPP), or single subject design (SSD), or historical comparisons were excluded.

#### 4.1.5 Types of comparison conditions

For RCT and QED studies, wait list control, no treatment, treatment‐as‐usual and alternative treatment groups were considered acceptable comparison groups.

#### 4.1.6 Other criteria

The reviewers anticipated making attempts to have non‐English articles translated; however, no eligible articles were located that required translation or inclusion in the excluded studies table.

#### 4.1.7 Duration of follow‐up

The reviewers included measurement points at post‐test and all follow‐up time points. We planned to synthesize studies that reported similar follow‐up time points (i.e., up to 3 months, 3–6 months, 6–12 months, >12 months) if there were more than two studies that reported sufficient data.

#### 4.1.8 Types of settings

All settings were eligible for inclusion.

### 4.2 Search methods for identification of studies

#### 4.2.1 Electronic searches

We conducted two identical rounds of electronic searches for two different time periods. In Round 1 searched for studies through May, 2017. Round 2 was an update to our search for studies from May, 2017 through May, 2020. For Round 1 and Round 2, we identified and retrieved both published and unpublished studies, including conference proceedings, through a comprehensive search that included multiple electronic databases, grey literature sources, and reference lists of reviews and relevant studies (Kugley et al., 2017).

**Figure 2:**
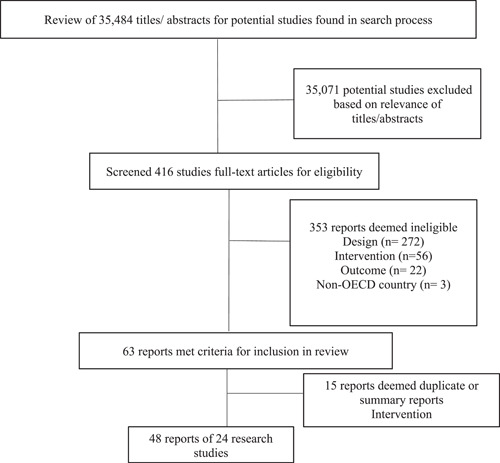
Study screening process

The search strategy and results of the search are documented in a detailed flow chart (Figure 2: Study Screening Process).

Table 1 contains the identity of the electronic databases and research registries, and government (e.g., the Consumer Financial Protection Bureau, Federal Reserve Banks) and organizational websites searched for this study. The databases were searched using key terms, as well as the names of specific types of financial capability interventions (e.g., ‘Child Development Account’ and ‘pre‐purchase homeownership education’).

**Table 1:** Search information

*Name of Database*	
ABI/INFORM	ERIC
Academic Search Complete	JSTOR
Bloomberg Professional Service	PAIS Index
Business Source Premier	PsychInfo
Database of Research on International Education	Public Affairs Index
Dissertation & Theses Global	Social Science Research Index
EconLit	Social Sciences Citation Index
Education Source	Social Work Abstracts
	Sociological Abstracts
*Research Registries*	
The National Institute for Health US National Library of Medicine (Clinicaltrials. gov)	
The International Clinical Trials Registry Platform (https://www.who.int/clinical-trials-registry-platform)	
*Government Websites*	
Consumer Financial Protection Bureau (https://www.consumerfinance.gov/)	
Federal Reserve Banks (12) (https://www.federalreserve.gov/aboutthefed/federal-reserve-system.htm)	
US Treasury Department (https://home.treasury.gov/)	
*Organization Websites*	
Alliance for Financial Inclusion (AFI) (https://www.afi-global.org/)	
Center for Social Development (https://csd.wustl.edu/)	
CGAP (https://www.cgap.org/)	
Citi Foundation (https://www.citigroup.com/citi/foundation/)	
Employee Benefit Research Institute (https://www.ebri.org/)	
Global Partnership for Financial Inclusion (https://www.gpfi.org/)	
Micro Financing Opportunities (https://www.microfinanceopportunities.org/)	
Russell Sage Foundation (https://www.russellsage.org/)	
Urban Institute (https://www.urban.org/)	
*Journal Hand Searched*	
Journal of Family and Economic Issues (Vol 31(1) – 41(1))	
Journal of Community Practice (Vol 18(1) – 28(2))	
Journal of the Society for Social Work and Research (1(1) – 11(2))	
Journal of Community Practice (Vol 44(1) – 54(1))	
Journal of Poverty (Vol 14(1)‐24(3))	
*Experts Contacted*	
Beverly, Sandra (Center for Social Development, Washington University in St Louis)	
Collins, J. Michael (University of Wisconsin‐Madison)	
Dubnicki, Alissa Louise (Syracuse University)	
Grinstein‐Weiss, Michal (Social Policy Institute, Washington University in St. Louis)	
Huang, Jin (Saint Louis University School of Social Work)	
McKernan, Signe‐Mary (Urban Institute)	
Mills, Gregory (Urban Institute)	
Modestino, Alicia S. (Northeastern University)	
Moulton, Stephanie (Ohio State University)	
Ratcliffe, Caroline (Urban Institute)	
Roll, Stephen (Social Policy Institute, Washington University in St. Louis)	
Tufano, Peter (Said Business School, University of Exford)	

In addition to databases and organizational websites, research registries and websites, the reference lists from prior reviews (Fernández‐Olit et al., 2019; Kasier & Mankhoff, 2016, 2019; Miller et al., 2014) and included studies were harvested for potential studies. We conducted forward citation searching using Google Scholar to search for studies citing the included studies. We also conducted a search on Google using key terms. The Google Scholar search included conference proceedings. We hand searched the table of contents of selected journals to identify potentially eligible reports not properly indexed (see Table 1 for more details). Our search for conference proceeding by Google Scholar and hand searching of relevant journals did not find any additional studies from the results of our search of the databases, websites, reference lists of prior reviews, Google Scholar and Google.

Finally, experts who were study or sub‐study authors of prior studies were contacted in an attempt to obtain unpublished studies, studies in process and published studies missed in the database search. Contact was attempted with 12 experts (i.e., study authors) through email. Each expert was contacted twice by email with a request for information about unpublished studies, studies in process and published studies missed in the database, as well as missing data. If there was no response, no further contact was made. No additional unpublished studies were provided by any author from within our time frames (see Table 1 for more details).

##### 4.2.1.1 Search terms and keywords

We used combinations of terms related to the intervention and study design to search the electronic databases. Database‐specific strategies were explored for each database in consultation with a librarian at Saint Louis University, including the use of truncation and database‐specific limiters, and thesauri were consulted to employ more precise search strategies within each database. Below are examples of the types of terms we used (see Appendix A for more details about database searches):

Intervention: (financial OR economic OR bank) AND (education OR knowledge OR literacy), AND (capability OR access OR inclusion OR exclusion OR attachment)

AND

Report type: (evaluation OR intervention OR treatment OR outcome OR program OR trial OR experiment OR ‘control group’ OR ‘controlled trial’ OR ’quasi‐experiment’ OR random*)

The databases were also searched using the following names of specific types of related interventions: ‘Individual Development Accounts’, ‘Child Development Accounts’, ‘credit counseling’, ‘pre‐purchase’ and home OR house, ‘second chance’ or ‘2nd chance’ and accounts, workplace OR ‘employer‐sponsored’ AND retirement OR savings, and ‘financial education’.

### 4.3 Data collection and analysis

#### 4.3.1 Selection of studies

The lead reviewer, Julie Birkenmaier, conducted the initial search in all sources between January and May 2017 for Round 1, and between May 2017 and May 2020 for Round 2. For each of the two rounds, the search results were saved in an electronic format using the reference manager software Endnotes. At this stage, the lead reviewer examined titles and abstracts and discarded results that were obviously ineligible (non‐empirical report, book review, editorial, non‐OECD country, etc.). For those that were not obviously ineligible, the reviewer retrieved the reports, saved them in an electronic file, removed duplicates, and documented the bibliographic information, source, and date retrieved. The data was then uploaded into the online platform Covidence. Julie Birkenmaier and another content reviewer, Youngmi Kim, independently screened each of the reports for eligibility using a screening instrument (see Supporting Information Appendix B). The two reviewers compared the coding and identified all discrepancies. The review team discussed and resolved all discrepancies through consensus. Any included study coauthored by one of the two reviewers was only screened by a non‐author (e.g., Youngmi Kim screened and coded studies co‐author by Julie Birkenmaier). Another reviewer, Brandy Maynard, screened as the second reviewer for those studies co‐authored by Julie Birkenmaier or Youngmi Kim.

#### 4.3.2 Data extraction and management

Two reviewers independently coded all studies that passed the eligibility screening process described above using a structured data extraction form (see Supporting Information Appendix C). The coders pilot tested the code form together using diverse types of studies and discussed any items that were unclear and ensured mutual understanding of all items. Following pilot testing of the form, the two coders independently coded 100% of the included studies. The coders compared coding and identified and discussed discrepancies, which were resolved through consensus.

Any study coauthored by one of the reviewers was coded by two other reviewers (e.g., Youngmi Kim and Brandy Maynard coded studies that were co‐author by Julie Birkenmaier, and vice versa). Multiple reports on individual studies were collated. The data extraction form included items related to bibliographic information and source descriptors, methods and procedures, context, nature, and implementation of the intervention, sample characteristics, and outcome data needed to calculate effect sizes.

#### 4.3.3 Assessment of risk of bias in included studies

##### Risk of bias

Three review authors participated in risk of bias assessment. At least two review authors who were not study authors independently assessed risk of bias in all included studies using the Cochrane Collaboration's risk of bias tool (Higgins et al., 2011). The review authors assessed risk of bias for each of the seven following domains: sequence generation, allocation concealment, blinding of participants and personnel for all outcomes, blinding of outcome assessors for all outcomes, incomplete outcome data for all outcomes, selective outcome reporting, and other potential sources of bias (i.e., researcher allegiance, funding source). Each study was coded as ‘low’, ‘high’, or ‘unclear’ risk of bias on each of the domains. Following independent coding by at least two review authors, they met to identify and resolve any discrepancies through consensus. We anticipated that most studies included in this review would be at high risk of bias in terms of allocation and blinding; thus, we did not plan to restrict analyses based on risk of bias in any domain.

#### 4.3.4 Synthesis procedures and statistical analysis

The review authors conducted descriptive analyses on variables of interest from all included studies to provide information regarding: (1) publication type; (2) study design; (3) sample size; (4) target beneficiaries; (5) race; (6) gender; (7) income; (8) intervention target; (9) intervention goal; (10) financial capability intervention type; (11) financial education; and (12) age of beneficiaries.

Following descriptive analysis, we examined the data to calculate effect sizes and prepare for the meta‐analysis. All effect sizes were calculated using the Practical Meta‐Analysis Effect Size Calculator (Wilson, n.d.). For continuous outcomes, we used author reported means and standard deviations to calculate the effect size when possible. When means and standard deviations were not reported, we calculated the ES using other data when possible. In one case, dichotomous data were reported (e.g., number of participants who opened or did not open an account) and thus we calculated those effect sizes using a 2 × 2 frequency table and converted to d using the Practical Meta‐Analysis Effect Size Calculator. We provided a narrative summary and tables describing the study characteristics and effect sizes (or primary author reported results when we could not calculate an effect size) for each of the outcomes of interest. Effect sizes could not be calculated for some studies due to authors not reporting sufficient information (most commonly, authors not reporting standard deviations). Review authors contacted study authors for missing information and in one case, the author did respond with more data.

As discussed in the Results section, a meta‐analysis could not be conducted due to the diversity of types of interventions that did not make sense to pool (e.g., effects of college savings with retirement accounts) and/or a lack of study authors reporting similar outcomes and/or similar time frames. For example, effect sizes were able to be calculated for saving amounts for three studies, but this outcome is measured across three distinct types of interventions. While the studies in this review met the criteria, the included studies reported on interventions that were disparate in terms of the financial product, the targeted population, and goals. Thus, we chose to group the interventions in a way that would be more meaningful and useful to practitioners and policy makers; therefore, we decided not to pool effects across these different categories of interventions with different intervention goals (i.e., saving for child's college education, saving for retirement, lowering debt, etc.).

##### Deviations from the protocol

The review authors followed the protocol (Birkenmaier et al., 2019) with three deviations: (1) A second round of search and extraction for sources between May 2017 to May 2020 was conducted; (2) effect sizes were not pooled across different types of interventions; and (3) because the studies had larger sample sizes (i.e., all >150), we did not see the need to correct for small sample size bias using Hedges' *g* as planned.

##### Criteria for determination of independent findings

The review authors were interested in two primary outcome constructs: behaviour change and change in access to a financial product or service resulting in financial outcome changes. The review authors anticipated that some included studies may use multiple measures for each outcome, multiple reports of the same outcome measure, multiple follow‐up time points, more than one intervention, and possibly more than one counterfactual condition. These circumstances create statistical dependencies that violate assumptions of standard meta‐analytic methods. To ensure independence of study‐level effect sizes, we included only one effect size estimate from each independent sample at each distinct measurement point (e.g., post‐test, follow‐up). Although we were not able to conduct a meta‐analysis, we were very attentive to ensuring that we were not counting multiple reports as independent included studies.

## 5 RESULTS

### 5.1 Results of search

#### 5.1.1 Terms used in this final report

This review reports on 24 studies, six of which are large longitudinal studies with multiple sub‐studies. The following terms are used to assist with clarity in this report:

*Unique Studies* have unique samples. (There are 24 in this review).

*Large Longitudinal Studies* have unique samples, but are reported on through sub‐studies resulting from using different time points, subsamples, or outcomes in the analysis. [There are six large longitudinal studies in this review (included in the 24 unique studies), reported on in 28 sub‐studies].

*Sub‐studies* are those studies resulting from a large longitudinal study project that use the same samples in various ways. (There are 28 sub‐studies in this review). The sub‐studies used sub‐samples from the larger longitudinal study, the same samples with different reported outcomes, or the same outcomes at different time points.

*Reports* refers to publicly available summary documents of included studies (a total of 63 reports with duplicates and summary reports, and 48 unduplicated reports in this review).

#### 5.1.2 Study names

Some studies that are well known by their program name will be referred to by their program name (e.g., SEED OK). Other studies do not have program names, and will be referred to by the last name of the first author.

Figure 2 provides a flow chart of the search and selection process. As seen in Figure 2, electronic searches of bibliographic databases and searches of other sources identified a total of 35,484 hits. Titles and abstracts were screened for relevance and 35,071 were excluded as duplicates or deemed inappropriate. The full text of the remaining 416 potential studies was reviewed and screened for eligibility by two independent coders. We excluded 353 reports that were deemed ineligible (see Supporting Appendix D) and included 63 reports that met inclusion criteria. Of the 63, 15 reports were deemed duplicates or summary reports. Of the remaining 48 reports, 24 were unique studies (using unique samples) that were included in this review. Six of those 24 studies were large longitudinal studies that presented unique analyses (using different time points, subsamples, and/or outcomes). Thus, we extracted data from 48 reports, reporting data and analyses from 24 unique studies.

#### 5.1.3 Included studies

##### Study characteristics

###### 5.1.3.1 Unique studies

Twenty‐four unique studies met inclusion criteria (see Supporting Information Appendix E for a list of reports that reported on the included studies, and Table 2 for a summary of characteristics of all included studies). Six of the 24 unique studies were large longitudinal studies (reporting unique analyses in 28 sub‐studies). These include the American Dream Demonstration Project (9 sub‐studies), Credit Building in Individual Development Account (IDA) Programs (3 sub‐studies), SEED for Oklahoma Kids Child Development Account Experiment (9 sub‐studies), Michigan SEED Children's Savings Program (2 sub‐studies), the Parks Opportunity Program (3 sub‐studies), and the Assets for Independence Program (2 sub‐studies).

**Table 2:** Characteristics of included studies across studies (*k* = 24). Characteristics by 24 unique study projects

	*k*	%		*k*	%
**Study characteristics**					
**Large longitudinal study projects (*k* = 6, 25% of total studies)**	**Sub‐studies**		**Sample size**		
American Dream Demonstration Project	9	NA	164–426	5	21
SEED for Oklahoma Kids Child Development Account Experiment	9	NA	427–850	4	17
Credit Building in Individual Development Account (IDA) Program	3	NA	1000–5500	8	33
Michigan SEED Children's Savings Program	2	NA	13,000–17,000	3	13
Parks Opportunity Program	3	NA	95,000–700,000	4	17
Assets for Independence	2	NA			
**Study design**			**Targeted special populations**		
Randomized control trial (RCT)	17	71	Low‐income	2	8
Quasi‐experiment design (QED)	7	29	Newborns	1	4
			Low and moderate‐income	7	29
			Employees	3	13
			Head Start Families	1	4
			Credit card holders	1	4
			Renters	1	4
			Debtors	1	4
			School children	1	4
			Unemployed	1	4
			Youth in workforce development	1	4
			None (General)	2	8
			Adult in workforce development	1	4
			First‐time home buyer	1	4
**Participant characteristics**					
**Age of beneficiaries**			**Income**		
Infant‐pre‐kindergarten	2	8	Low	8	33
Kindergarten‐5th grade	1	4	Low‐Moderate	8	33
Adults	18	75	All incomes	6	25
Unknown	1	4	Unknown	2	8
Multiple	1	4			
**Predominant subject race**			**Previous financial education assessed pre‐intervention**		
White	8	33	Unknown	23	96
African American	5	21			
Unknown	8	33			
Other	2	8			
Hispanic	1	4			
**Intervention characteristics**					
**Intervention targeted to people without**:			**Financial capability intervention**		
Assets—General	9	38	Matched Savings Accounts (Individual Development Accounts)	4	17
Education savings	3	13	Child Development Accounts	3	13
Credit	4	17	Retirement accounts	4	17
Retirement savings	4	17	Youth bank account	1	4
Bank account	2	8	Pre‐purchase homeownership education	1	4
Home mortgage	2	8	Adult financial education, counselling and coaching	6	25
			Tax refund saving and investment	5	21
**Intervention goal**			**Financial education provided**		
Bank Account	2	8	Low intensive	11	46
Savings/investment	9	38	High intensive	13	54
Education savings	3	13			
Retirement savings	4	17			
Strong credit	4	17			
Home mortgage	2	8			
**Outcome studied**					
**Behaviour change**			**Financial outcomes**		
Account opening	7	29	Saving amount	18	75
Retirement savings rate	3	13	Debt amount	5	21
Saving rate	1	4	Credit score	7	29
Budget	2	8	Asset value	3	13
Purchased asset	3	13			

As seen in Table 2, the majority of the 24 unique studies were randomized control trials (*k* = 17, 71%), and the remaining were quasi‐experimental designs (*k* = 7, 29%). Sample sizes were wide‐ranging: 1–400 (*k* = 5, 21%), 401–850 (*k* = 4, 17%), 1000–5500 (*k* = 8, 33%), 13,000–17,000 (*k* = 3, 13%) and 95,000–700,000 (*k* = 4, 17%). There were many different targeted special populations for the interventions: Low‐income (*k* = 2, 8%); newborns (*k* = 1, 4%); low and moderate‐income households (*k* = 7, 29%), employees, (*k* = 3, 13%), Head Start families (*k* = 1, 4%), credit card holders (*k* = 1, 4%), renters (*k* = 1, 4%), debtors (*k* = 1, 4%), school children (*k* = 1, 4%), unemployed (*k* = 1, 4%), youth in a workforce development program (*k* = 1, 4%), adult in workforce development program (*k* = 1, 4%), first time homebuyer (*k* = 1, 4%) and 2 studies had no targeted population (general public) (8%).

###### 5.1.3.2 Sub‐studies

Large longitudinal studies resulted in 28 sub‐studies. The sub‐studies used sub‐samples from the larger longitudinal study, the same samples with different reported outcomes, or the same outcomes at different time points.

The American Dream Demonstration Project (ADD) large longitudinal study resulted in nine sub‐studies (Grinstein‐Weiss et al., 2008, 2012, 2013a, 2015; Han et al., 2009; Huang, 2010; Huang et al., 2016; Lombe, 2004; Mills et al., 2006). The intervention was a randomized control trial that targeted low‐income people. As seen in Table 3, the sub‐studies were reported as journal articles (78%), unpublished reports (11%) and other (Powerpoint presentation) (11%). Most of the sub‐studies were conducted 10 years after baseline (44%), followed by 4 years post‐baseline (33%), and the remaining used the two time points of 18 months and 4 years post‐baseline (22%). The dominant sample sizes were 500–799 (33%) and 800–1000 (33%). High‐intensity financial education was provided in the intervention (100%).

**Table 3:** Characteristics of sub‐studies from american dream demonstration project (*k* = 9)

	*k*	%		*k*	%
**Study characteristics**					
**Time point post‐baseline**			**Sample size**		
4 years	3	33	336–499	2	22
18 months and 4 years	2	22	500–799	3	33
10 years	4	44	800‐1000	3	33
			1001 and over	1	11
**Publication type**					
Journal	7	78	**Financial education**		
Unpublished report	1	11	High intensity	9	100
Other	1	11			
**Participant characteristics**					
**Age of beneficiaries**			**Income**		
Range of 24‐45	1	11	Low	9	100
34‐38 years old	6	67			
39+	2	22	**Gender (% male)**		
			17	2	22
			20‐21	6	67
			Not reported	1	11
**Predominant subject race**			**Previous financial education assessed pre‐intervention**		
White	9	100	Unknown	9	100
**Outcomes studied**					
**Behaviour change**			**Financial outcomes**		
Purchased asset	7	78	Saving amount	1	11
(Homeownership)	3	33	Debt amount	2	22
(Education)	1	11	Asset value	3	33
(Retirement savings)	1	11			
Retirement savings rate	1	11			
**Quality measures**					
**Results of statistical comparisons of pre‐test differences on outcomes**			**Results of statistical comparisons of pre‐test differences on demographics**		
No statistical comparison made	2	22	No statistical comparison made	0	0
No statistically significant differences	5	56	No statistically significant differences	0	0
Statistically significant differences found	2	22	Statistically significant differences found	9	100

The Credit Building in Individual Development Account (IDA) Program (Credit Building) resulted in three sub‐studies (Birkenmaier et al., 2012, 2014a, 2014b). This intervention also targeted low‐income people, and was also a randomized control trial. As seen in Table 4, the three sub‐studies were reported as journal articles (100%). The sub‐studies were conducted 1, 2 and 3 years post‐baseline. The sample size was 164‐165 for all of the sub‐studies (100%). High‐intensity financial education was provided to the participants (100%).

**Table 4:** Characteristics of sub‐studies from Credit Building in Individual Development Account (IDA) programs (*k* = 3)

	*k*	%		*k*	%
**Study characteristics**					
**Time point post‐baseline**			**Sample size**		
1 year	1	33	164–165	3	100
2 years	1	33			
3 years	1	33			
			**Financial education**		
**Publication type**			High intensity	3	100
Journal	3	100			
**Participant characteristics**					
**Age of beneficiaries**			**Income**		
31–41 (mean)	3	100	Low	3	100
**Predominant subject race**			**Gender (% male)**		
African American	3	100	13%	2	67
			Not reported	1	11
			**Previous financial education assessed pre‐intervention**		
			Unknown	3	100
**Outcomes studied**					
			**Financial outcomes**		
			Credit score	3	100
**Quality measures**					
**Results of statistical comparisons of pre‐test differences on outcomes**			**Results of statistical comparisons of pre‐test differences on demographics**		
No statistical comparison made	2	66	No statistical comparison made	2	66
No statistically significant differences	1	33	No statistically significant differences	0	0
Statistically significant differences found	0	0	Statistically significant differences found	1	33

The SEED for Oklahoma Kids Child Development Account Experiment (SEED OK) large longitudinal study was a randomized control trial that resulted in nine sub‐studies (Beverly et al., 2014; Beverly, Kim, et al., 2015; Clancy et al., 2016; Huang et al., 2013, 2015, 2017; Huang et al., 2019; Nam et al., 2013; Wikoff et al., 2015). The intervention targeted newborn children. As seen in Table 5, the sub‐studies were reported as journal articles (89%) and unpublished reports (11%). Most of the sub‐studies were conducted 18 months–3 years after baseline (44%), followed by 6–7 years post‐baseline (33%) and 4–5 years (22%). The sample size was 0–450 (*k* = 1, 11%), 2626–2700 (*k* = 5, 56%) and 2701–2704 (*k* = 3, 33%). The sub‐studies reported on both participant‐owned accounts (100%) and state‐owned account (67%). Low‐intensity financial education was provided in the intervention (100%).

**Table 5:** Characteristics of sub‐studies from Saving for Education, Entrepreneurship and Down payment (SEED) OK experiment (*k* = 9)

	*k*	%		*k*	%
**Study characteristics**					
**Time point post‐baseline**			**Sample size**		
18 months–3 years	4	44	426	1	11
4–5 years	2	22	2626–2700	5	56
6–7 years	3	33	2701–2704	3	33
			**Account type studied**		
**Publication type**			Participant‐owned	9	100
Journal	8	89	State‐owned	6	67
Unpublished report	1	11			
			**Financial education**		
			Low‐intensity	9	100
**Participant characteristics**					
**Age (parents)**			**Income**		
20–29 years old	7	78	All	8	89
Not reported	2	22	Low‐income	1	11
			**Gender (% male)**		
			52%–54%	4	44
			Not reported	4	44
			No males	1	11
**Predominant subject race**			**Previous financial education assessed pre‐intervention (parents)**		
White	9	100	Unknown	8	89
**Outcomes studied**					
**Behaviour change**			**Financial outcomes**		
Account Opening (Participant Owned)	6	67	Saving amount	6	67
			Asset value	2	22
**Quality measures**					
**Results of statistical comparisons of pre‐test differences on outcomes**			**Results of statistical comparisons of pre‐test differences on demographics**		
No statistical comparison made	6	67	No statistical comparison made	0	0
No statistically significant differences	1	11	No statistically significant differences	2	22
Statistically significant differences found	2	22	Statistically significant differences found	7	78

The Michigan SEED Children's Savings Program (MI SEED) was also a randomized control trial, and resulted in two sub‐studies (Engelhardt et al., 2012; Marks et al., 2009). This intervention targeted families involved with the (federal preschool) Head Start program. As seen in Table 6, the two sub‐studies were reported as unpublished reports (100%). The sub‐studies were conducted 4 years post‐baseline. The sample size was 600 and 628 for the sub‐studies (100%), and only participant‐owned accounts were studied (100%). Low‐intensity financial education was provided to the participants (100%).

**Table 6:** Characteristics of sub‐studies from Michigan Saving for Education, Entrepreneurship and Downpayment (MI‐SEED) experiment (*k* = 2)

	*k*	%		*k*	%
**Study characteristics**					
**Time point post‐baseline**			**Sample size**		
4 years	2	100	600	1	50
			628	1	50
			**Account type studied**		
**Publication type**			Participant‐owned	2	100
Unpublished report	2	100			
			**Financial education**		
			Low‐intensity	2	100
**Participant characteristics**					
**Age (parents)**			**Income**		
30–35 years old	2	100	All	1	50
			Not reported	1	50
**Predominant subject race**					
White	1	50	**Gender (% male)**		
Not reported	1	50	9%–10%	1	50
			Not reported	1	50
			**Previous financial education assessed pre‐intervention (parents)**		
			Unknown	2	100
**Outcomes studied**					
**Behaviour change**			**Financial outcomes**		
Account Opening	1	50	Saving amount	2	100
Budgeting	1	50			
**Quality measures**					
**Results of statistical comparisons of pre‐test differences on outcomes**			**Results of statistical comparisons of pre‐test differences on demographics**		
No statistical comparison made	1	50	No statistical comparison made	0	0
No statistically significant differences	0	0	No statistically significant differences	1	50
Statistically significant differences found	1	50	Statistically significant differences found	1	50

The Parks Opportunity Program, also known as the ‘Assessing Financial Capabilities Outcomes Adult Pilot’, was a randomized control trial. The study resulted in three sub‐studies (Collins & Nafziger, 2019; Gons, 2013; Wiedrich et al., 2014). The intervention targeted employees. As seen in Table 7, one sub‐study was reported as an unpublished report (33%), one study was a journal article (33%), and the third as ‘other’ (presentation) (33%). The sub‐studies were conducted at 6 and 12 months, and 9 months post‐baseline. The sample size was 865 (*k* = 1, 25%) and 1034 (*k* = 2, 75%).

**Table 7:** Characteristics of sub‐studies from Parks Opportunity Program (also known as the 'Assessing Financial Capabilities Outcomes Adult Pilot') (*k* = 3)

	*k*	%		*k*	%
**Time point post‐baseline**			**Sample size**		
6 and 12 months	2	75	865	1	25
9 months	1	25	1034	2	75
**Publication type**			**Financial education**		
Journal article	1	33	High intensity	3	100
Other (presentation)	1	33			
Unpublished report	1	33			
**Participant characteristics**					
**Mean age of beneficiaries**			**Income**		
36	3	100	Low	3	100
			**Gender (% male)**		
			21–22	3	100
**Predominant subject race**			**Previous financial education assessed pre‐intervention**		
African American	2	75	Unknown	3	100
Not reported	1	25			
**Outcomes studied**					
			**Financial outcomes**		
			Saving amount	1	33
			Debt amount	2	66
			Credit score	3	100
**Selected quality measures**					
**Results of statistical comparisons of pre‐test differences on outcomes**			**Results of statistical comparisons of pre‐test differences on demographics**		
No statistical comparison made	2	75	No statistical comparison made	2	75
No statistically significant differences	1	25	No statistically significant differences	0	0
Statistically significant differences found	0	0	Statistically significant differences found	1	25

The Assets for Independence Program was a randomized control trial. As seen in Table 8, the study resulted in two sub‐studies (Mills et al., 2016; Ratcliffe et al., 2019). Both sub‐studies were reported as unpublished reports (100%). The sub‐studies were conducted at 1 and 3 years post‐baseline. The sample size was 621 (*k* = 1, 50%) and 807 (*k* = 1, 50%).

**Table 8:** Characteristics of sub‐studies from Assets for Independence program (*k* = 2)

	*k*	%		*k*	%
**Study characteristics**					
**Time point post‐baseline**			**Sample size**		
1 year	1	50	621	1	50
3 years	1	50	807	1	50
**Publication type**			**Financial education**		
Unpublished report	2	100	High intensity	2	100
**Participant characteristics**					
**Mean age of beneficiaries**			**Income**		
Unreported	2	100	Low	2	100
			**Gender (% male)**		
			28.2	1	50
			30	1	50
**Predominant subject race**			**Previous financial education assessed pre‐intervention**		
Hispanic	2	100	Unknown	2	100
**Outcomes studied**					
**Behaviour change**			**Financial outcomes**		
Purchase asset	2	100	Saving amount	2	100
Homeownership	2	100			
Business ownership	2	100			
Post‐secondary education	2	100			
**Selected quality measures**					
**Results of statistical comparisons of pre‐test differences on outcomes**			**Results of statistical comparisons of pre‐test differences on demographics**		
No statistical comparison made	2	100	No statistical comparison made	1	50
No statistically significant differences	0	0	No statistically significant differences	0	0
Statistically significant differences found	0	0	Statistically significant differences found	1	50

#### 5.1.4 Participant characteristics

5.1.4.1

###### 5.1.4.1.1 Unique studies

In the 24 unique studies, most of the study participants were adults (*k* = 18, 75%), followed by infant to pre‐kindergarten (*k* = 2, 8%), kindergarten through fifth grade (*k* = 1, 4%), unknown (*k* = 1, 4%) and multiple (*k* = 1, 4%) at the time of the beginning of the study. The predominant race for the majority of the study projects was White (*k* = 8, 33%), followed by unknown (*k* = 8, 33%), African American (*k* = 5, 21%), Hispanic (*k* = 1, 4%) and other (*k* = 2, 8%). Most of the unique studies had subjects of low‐income (*k* = 8, 33%) and low‐moderate income (*k* = 8, 33%), followed by all incomes (*k* = 6, 25%), and two (8%) had unknown income. The majority of unique studies (*k* = 23, 96%) did not assess whether the participants previously had received financial education.

####### 5.1.4.1.1.1 Sub‐studies

As seen in Table 3, in the nine sub‐studies resulting from the American Dream Demonstration Project (ADD) large longitudinal study, the predominant race for the majority of the study projects was White (*k* = 59, 100%). Study participants were adults who were predominately 34–38 years old (67%). All of the sub‐studies had low‐income participants. Most of the sub‐studies had a sample of 20% males (67%). None of the sub‐studies assessed whether the participants previously had received financial education.

As displayed in Table 4, in the three sub‐studies resulting from the Credit Building in Individual Development Account (IDA) Program [Credit Building] large longitudinal study, the predominant race for the majority of the study projects was African American (100%). Study participants were predominately 31–41 years old (100%). All of the sub‐studies had participants with low incomes. Two of the sub‐studies had a sample of 13% males (67%), while the gender breakdown was not reported for the third sub‐study. None of the sub‐studies assessed whether the participants previously had received financial education.

As seen in Table 5, in the nine sub‐studies resulting from the SEED for Oklahoma Kids Child Development Account Experiment (SEED OK) large longitudinal study, study participants' parents were predominately 20–29 years old (78%), and all of the participant children were born in 2007. The dominant sample sizes were 2626–2700 (56%) and 2701–2704 (33%). The predominant race for the majority of the study projects was White (100%). 89% of the sub‐studies had participants with all income levels. 44% of the sub‐studies had a sample of 52%–54% males, while the gender breakdown was not reported for 44%. The majority of the sub‐studies (89%), did not assess whether the participants previously had received financial education.

As displayed in Table 6, in the two sub‐studies resulting from the Michigan SEED Children's Savings Program (MI SEED) large longitudinal study, the predominant race for the majority of the study projects was White (50%) and not reported (50%). One sub‐studies had participants with all incomes, and the other was not reported. Study participants were predominately 31–41 years old (100%). One sub‐study reported that 9%–10% of the participants were male, and the other study did not report gender breakdown. Neither of the sub‐studies assessed whether the participants previously had received financial education.

As seen in Table 7, in the three sub‐studies resulting from the Parks Opportunity Program large longitudinal study, the predominant race was African American for two sub‐studies (75%) and not reported for the other (25%). Study participants were an average of 36 years old (100%) for all sub‐studies. All three of the sub‐studies had participants with low‐incomes, and the studies had 21%–22% males. None of the sub‐studies assessed whether the participants previously had received financial education.

As seen in Table 8, in the two sub‐studies resulting from the Assets for Independence Program Evaluation, the predominant race was Hispanic for both sub‐studies. The mean age for study participants was not reported for both sub‐studies. Both sub‐studies had participants with low incomes, and the studies had 28.2% and 30% males. None of the sub‐studies assessed whether the participants previously had received financial education.

#### 5.1.5 Intervention characteristics

5.1.5.1

###### 5.1.5.1.1 Unique studies

As seen in Table 2, the interventions examined in the 24 studies were targeted to people without assets (*k* = 9, 38%), education savings (*k* = 3, 13%), credit (*n* = k, 17%), retirement savings (*k* = 4, 17%), a bank account (*k* = 2, 8%) or a home mortgage (*k* = 2, 8%). The intervention goal for the majority of the studies was saving and investment (*k* = 9, 38%), retirement savings (*k* = 4, 17%), strong credit (*k* = 4, 17%), education savings (*k* = 3, 13%), a bank account (*k* = 2, 8%), and a home mortgage (*k* = 2, 8%). The interventions themselves were IDAs (*k* = 4, 17%), CDAs (*k* = 3, 13%), retirement accounts (*k* = 4, 17%), adult financial education, counselling or coaching (*k* = 6, 25%), tax refund saving and investment (*k* = 5, 21%), a bank account (*k* = 1, 4%), and homeownership education and counselling (*k* = 1, 4%). As by definition all of the financial capability interventions include financial education, the majority of the interventions included high intensive financial education methods (*k* = 13, 54%) such as classes or one‐on‐one teaching (e.g., Birkenmaier et al., 2012, 2014a, 2014b). Eleven studies (46%) used low intensive methods, such as a mailed flyer (Clancy et al., 2016) or explaining general savings principles while delivering a tax return product (Duflo et al., 2006).

#### 5.1.6 Outcome measures

In the 24 unique studies, data were collected on the outcomes of financial behaviour and financial outcomes of the study participants using unstandardized instruments, and including self‐reported and administrative data.

##### Financial behaviour change

Across the 24 unique studies, which were reported in the 48 reports (primary study and sub‐studies), five financial behaviours were measured in 13 measurements. Six studies measured bank account opening: Duflo et al. (2006); Engelhardt et al. (2012); Lusardi et al. (2009); MI SEED [Engelhardt et al., 2012]; Osborne et al., 2016; and SEED OK [multiple sub‐studies]. Two measured retirement savings rates: Collins and Urban (2016) and Goda et al. (2012). Two studies measured saving rate: ADD [Grinstein‐Weiss et al., 2015] and Grinstein‐Weiss, Cryer, et al. (2017). Three studies measured budgeting: Collins and Urban (2016); Kim et al. (2005); and MI SEED [Marks et al., 2009]. Three measured asset purchase (of homeownership, post‐secondary education and retirement savings): ADD [Grinstein‐Weiss et al., 2008, 2012, 2013a; Huang, 2010; Huang et al., 2016; Lombe, 2004; Mills et al., 2006); Assets for Independence [Mills et al., 2016; Ratcliffe et al., 2019]; Tufano, 2014.

The six bank account openings were measured at the following time points post‐intervention: (a) immediately (Duflo et al., 2006; (b) 10 weeks (Osborne et al., 2016); (c) end of treatment and 6 and 12 months later (Collins & Urban, 2016); (d) 18 months (SEED OK [Nam et al., 2013]); (e) 3 years [SEED OK (Huang et al., 2013)]; (f) 4 years (MI SEED [Engelhardt et al., 2012); (g) 4–5 years (SEED OK [Wikoff et al., 2015]); (h) 5 years (SEED OK [Huang et al., 2017]); and (i) 6 years (SEED OK [Huang et al., 2015]).

Retirement savings rate was measured at the following time points: (a) end of treatment, 6 and 12 months later (Collins & Urban, 2016); and (b) end of treatment (Goda et al., 2012). The saving rate was measured at end of treatment (Grinstein‐Weiss, Cryer, et al., 2017), and 10 years (Grinstein‐Weiss et al., 2015). Budgeting was measured at the following time points: (a) end of treatment at 6 and 12 months later (Collins & Urban, 2016); (b) 18 months (Kim et al., 2005); and 4 years (MI SEED [Marks et al., 2009]).

Asset purchase was measured at the following time points: (a) end of treatment (Tufano, 2011); (b) 1 year (Assets for Independence [Mills et al., 2016]); (c) 3 years (Assets for Independence [Ratcliffe et al., 2019]).

##### Financial outcomes

Across the 24 unique studies, which were reported in the 48 reports (primary study and sub‐studies), four financial outcomes were measured in 24 measurements.

Seventeen measured saving amounts: ADD [Grinstein‐Weiss et al., 2015]; Assets for Independence [Mills et al., 2016; Ratcliffe et al., 2019]; Parks Opportunity Program [Wiedrich et al., 2014]; Collins and Urban (2016); Duflo et al. (2006); Goda et al. (2012); Leckie et al. (2010a); Loke et al. (2016); MI SEED [Engelhardt et al., 2012; Marks et al., 2009; Osborne et al., 2016] SEED OK [Beverly et al., 2014; Beverly, Kim, et al., 2015; Clancy et al., 2016; Huang et al., 2015, 2017; Nam et al., 2013]; Grinstein‐Weiss et al., 2015; Grinstein‐Weiss, Cryer, et al., 2017; Grinstein‐Weiss, Russell, et al., 2017; Moulton et al., 2015; Roll et al., 2018; Smith et al., 2017; Tufano, 2014.

Five studies measured debt amounts: ADD [Grinstein‐Weiss et al., 2008; Huang, 2010]; Parks Opportunity Program [Collins & Nafzinger, 2019; Gons, 2013]; Kim et al. 2005; Smith et al., 2017; Theodos et al., 2016].

Seven measured credit score: Parks Opportunity Program [Collins & Nafzinger, 2019; Gons, 2013; Wiedrich et al., 2014]; Credit Building [Birkenmaier et al., 2012, 2014a, 2014b]; Modestino et al., 2019; Moulton et al., 2015; Roder, 2016; Smith et al., 2017; Theodos et al., 2016.

Three measured asset value: ADD [Han et al., 2009; Huang, 2010; Leckie et al., 2010a; Lombe, 2004]; and SEED OK [Huang et al., 2015, 2019].

Saving amounts were measured at the following time points: (a) end of treatment (Duflo et al., 2006; Goda et al., 2012; Grinstein‐Weiss et al., 2015; Grinstein‐Weiss, Cryer, et al., 2017; Grinstein‐Weiss, Russell, et al., 2017; Loke et al., 2016; Roll et al., 2018; Tufano, 2014); (b) end of treatment and 6 and 12 months later (Collins & Urban, 2016); Parks Opportunity Program [Collins & Nafzinge, 2019; Wiedrich et al., 2014]; (c) 10 weeks (Osborne et al., 2016); (d) 1 year (Assets for Independence [Mills et al., 2016]; and Moulton et al., 2015; (e) 18 months (Nam et al., 2013); (f) 30 months (SEED OK [Beverly et al., 2014]); (g) 3 years (SEED [Beverly, Kim, et al., 2015]), (Assets for Independence [Ratcliffe et al., 2019); (h) 4 years (Leckie et al., 2010a); and MI SEED [Engelhardt et al., 2012; Marks et al., 2009]); (i) 5 years (SEED [Huang et al., 2017], Smith et al., 2017); (j) 6 years (SEED [Huang et al., 2015]); (k) 7 years (SEED OK [Clancy et al., 2016]); and (l) 10 years (ADD [Grinstein‐Weiss et al., 2015]).

Debt amounts were measured at the following time points: (a) immediately after treatment (Theodos et al., 2016); (b) end of treatment and 6 and 12 months later (Parks Opportunity Program [Collins & Nafzinger, 2019; Wiedrich et al., 2014]); (c) 9 months (Parks Opportunity Program [Gons, 2013]); (d) 18 months (Kim et al., 2005); (e) 18 and 48 months (ADD [Huang, 2010]); (f) 4 years (ADD [Grinstein‐Weiss et al., 2008]); and (g) 5 years (Smith et al., 2017]).

Credit score was measured at the following time points: (a) 6 months before and immediately after treatment (Theodos et al., 2016); (b) end of treatment and 6 and 12 months later (Parks Opportunity Program [Wiedrich et al., 2014]); (c) 9 months (Parks Opportunity Program [Gons, 2013]); (d) 1 year (Credit Building [Birkenmaier et al., 2012]) and Moulton et al., 2015; (e) 18 months (Modestino et al., 2019); (f) 2 years (Credit Building [Birkenmaier et al., 2014b]; Roder, 2016); (g) 3 years (Credit Building [Birkenmaier et al., 2014a]); and (h) 5 years (Smith et al., 2017) after intervention.

Asset value was measured at the following time points: (a) 18 months and 48 months (ADD [Huang, 2010]); (b) 4 years (ADD [Han et al., 2009; Lombe, 2004) and Leckie et al., 2010a; (c) 6 years (SEED OK [Huang et al., 2015]); and (d) 7 years (Huang et al., 2019).

##### 5.1.6.1 Unique studies

In the 24 unique studies, data were collected on financial behaviour and financial outcomes of the study participants using unstandardized instruments, and included self‐reported and administrative data. Six of the studies are large longitudinal studies, whose outcomes are reported in the sub‐studies (discussed next). As seen in Table 2, together with the remaining 18 studies, five behaviour changes were studied as outcome measures: bank or retirement account opening (*k* = 7, 29%), retirement saving rate (*k* = 3, 13%), saving rate (*k* = 1, 4%), budgeting (*k* = 2, 8%) and purchased asset (*k* = 3, 13%). The financial outcomes refer to implications of financial behaviour change. Savings amount (*k* = 18, 75%), debt amount (*k* = 5, 21%), credit score (*k* = 7, 29%), asset value (*k* = 3, 13%) were studied.

*Sub‐studies*—In the 28 sub‐studies of the six large longitudinal studies, data were also collected on financial behaviour and financial outcomes of the study participants using unstandardized instruments, and included self‐reported and administrative data. The data are displayed in Tables 3, 4, 5, 6, 7, 8. As Table 3 shows, in the nine sub‐studies resulting from the American Dream Demonstration Project large longitudinal study, in terms of behaviour outcomes, the majority studied the change of purchased asset (78%), including homeownership (*k* = 3, 33%), education (*k* = 1, 11%) and retirement savings (*k* = 1, 11%). Also studied was retirement savings rate (*k* = 1, 11%). Regarding financial outcomes, the largest number of sub‐studies focused on asset value (33%), followed closely by debt amount (22%) and saving amount (11%). As seen in Table 4, in the three sub‐studies resulting from the Credit Building in Individual Development Account (IDA) Program large longitudinal study, in terms of outcomes, all of the sub‐studies assessed financial outcomes of credit score (100%). Table 5 displays characteristics of the nine sub‐studies resulting from the SEED for Oklahoma Kids Child Development Account Experiment large longitudinal study. All sub‐studies assessed the behaviour change of account opening (100%). Regarding financial outcomes, the largest number of sub‐studies focused on saving amount (67%), and two studies focused on asset value (22%). As seen in Table 6, in the two sub‐studies resulting from the Michigan SEED Children's Savings Program large longitudinal study, in terms of behaviour change outcomes, one sub‐study assessed account opening (50%) and budgeting (50%). Relative to financial outcome, both studies assessed the financial outcome of saving amount (100%). As Table 7 displays, in the three sub‐studies resulting from the Parks Opportunity Program large longitudinal study, two studies assessed the financial outcome of debt amount (66%), three studies assessed credit score (100%), and one sub‐study assessed the saving amount (33%). Table 8 displays the characteristics of the Assets for Independence sub‐studies. Both assessed the financial outcomes of saving amount and purchase asset.

#### 5.1.7 Excluded studies

As seen in Figure 2, the 353 ineligible reports were excluded during full‐text review due to not meeting one or more of the following eligibility criteria: ineligible design (*k* = 272), ineligible intervention (*k* = 53), ineligible outcome (*k* = 22), and non‐OECD country (*k* = 3). Ineligible study designs included by reason of not quantitatively assessing the effects of an intervention. The ineligible designs include, for example, all forms of qualitative research, literature, conceptual essays, and policy research. See Supporting Information Appendix D for a full list of excluded studies/reports and reasons for exclusion).

### 5.2 Risk of bias in included studies

We used the Cochrane Risk of Bias tool (Higgins et al., 2011) that is commonly used to assess risk of bias in experimental studies of intervention effects. Although later versions are available, Campbell has not made a policy to adopt newer versions of the Cochrane Risk of Bias tool.

As seen in Table 9, the risk of bias varied across studies. The majority of the study designs were randomized controlled trials, and the remainder of the studies were quasi‐experimental designs with comparison groups.

**Table 9:** Risk of bias

Study Name: American Dream Demonstration Project (9 sub‐studies)		
Type of bias	Judgement	Support for judgement
Random sequence generation (selection bias)	Unclear risk	Non‐random assignment to groups in sub‐studies
Allocation concealment (selection bias)	Low risk	Allocation concealed
Blinding of participants and personnel (performance bias)	Unclear risk	Not reported if participants/personnel were blinded
Blinding of outcome assessment (detection bias)	Low risk	Outcome assessors were blinded
Incomplete outcome data (attrition bias)	High risk	Both tx and control group had high attrition (>20%)
Selective outcome reporting (reporting bias)	Unclear risk	Study protocol was not found
Other biases (research allegiance, funding, confounds)	High risk	Authors designed the treatment intervention

Study Name: Assets for Independence (2 sub‐studies)		
Type of bias	Judgement	Support for judgement
Random sequence generation (selection bias)	Low risk	Random assignment to groups
Allocation concealment (selection bias)	Unclear risk	Concealment not reported
Blinding of participants and personnel (performance bias)	Unclear risk	Not reported if participants/personnel were blinded
Blinding of outcome assessment (detection bias)	Low risk	Evaluator role independent of intervention
Incomplete outcome data (attrition bias)	High risk	Tx and comparison group had high attrition
Selective outcome reporting (reporting bias)	Unclear risk	Study protocol was not found
Other biases (research allegiance, funding, confounds)	Unclear risk	Unclear whether there were allegiance or funding confounds

Study Name: Collins and Urban (2016)		
Type of bias	Judgement	Support for judgement
Random sequence generation (selection bias)	High risk	Non‐random assignment to groups
Allocation concealment (selection bias)	Unclear risk	Allocation concealment not described
Blinding of participants and personnel (performance bias)	Unclear risk	Not reported if participants/personnel were blinded
Blinding of outcome assessment (detection bias)	Low risk	Assessors were blinded to group assignment
Incomplete outcome data (attrition bias)	Unclear risk	Attrition rate not reported
Selective outcome reporting (reporting bias)	Unclear risk	Study protocol was not found
Other biases (research allegiance, funding, confounds)	High risk	Authors designed the treatment intervention

Study Name: Credit Building in Individual Development Account (IDA Programs (3 sub‐studies)		
Type of Bias	Judgement	Support for judgement
Random sequence generation (selection bias)	High risk	Non‐random assignment to groups
Allocation concealment (selection bias)	Unclear risk	Allocation concealment procedures not described
Blinding of participants and (performance bias)	Unclear risk	Not reported if personnel participants/personnel were blinded
Blinding of outcome assessment (detection bias)	Low risk	Outcome assessors were blinded
Incomplete outcome data (attrition bias)	Low risk	Low attrition
Selective outcome reporting (reporting bias)	Low risk	Study protocol was followed
Other biases (research allegiance, funding, confounds)	Low risk	No allegiance or funding confounds

Study Name: Duflo et al. (2006)		
Type of bias	Judgement	Support for judgement
Random sequence generation (selection bias)	Low risk	Random assignment to groups
Allocation concealment (selection bias)	Unclear risk	Allocation concealment not described
Blinding of participants and personnel (performance bias)	Unclear risk	Not reported if participants/personnel were blinded
Blinding of outcome assessment (detection bias)	Low risk	Outcome assessors were blinded
Incomplete outcome data (attrition bias)	Low risk	Low attrition
Selective outcome reporting (reporting bias)	Unclear risk	Study protocol was not found
Other biases (research allegiance, funding, confounds)	High risk	Authors designed the treatment intervention

Study Name: Goda et al. (2012)		
Type of bias	Judgement	Support for judgement
Random sequence generation (selection bias)	Low risk	Random assignment by departments and matched‐quads
Allocation concealment (selection bias)	Low risk	Allocation based on department and demographics
Blinding of participants and personnel (performance bias)	Unclear risk	Not reported if participants/personnel were blinded
Blinding of outcome assessment (detection bias)	Low risk	Assessors were blinded to group assignment
Incomplete outcome data (attrition bias)	Unclear risk	Attrition rate not reported
Selective outcome reporting (reporting bias)	Unclear risk	Study protocol was not found
Other biases (research allegiance, funding, confounds)	Unclear risk	Unclear whether there were allegiance or funding confounds

Study Name: Grinstein‐Weiss, Cryer, et al. (2017)		
Type of bias	Judgement	Support for judgement
Random sequence generation (selection bias)	Low risk	Random assignment to groups
Allocation concealment (selection bias)	Low risk	Concealment occurred
Blinding of participants and personnel (performance bias)	Low risk	Participants/personnel were blinded
Blinding of outcome assessment (detection bias)	Low risk	Evaluator role independent of intervention
Incomplete outcome data (attrition bias)	Low risk	Tx and comparison group had no attrition
Selective outcome reporting (reporting bias)	Unclear risk	Study protocol was not found
Other biases (research allegiance, funding, confounds)	Unclear risk	Unclear whether there were allegiance or funding confounds)

Study Name: Grinstein‐Weiss et al. (2015)		
Type of bias	Judgement	Support for judgement
Random sequence generation (selection bias)	Low risk	Random assignment to groups
Allocation concealment (selection bias)	Low risk	Concealment occurred
Blinding of participants and personnel (performance bias)	Low risk	Participants/personnel were blinded
Blinding of outcome assessment (detection bias)	Low risk	Evaluator role independent of intervention
Incomplete outcome data (attrition bias)	Low risk	Tx and comparison group had no attrition
Selective outcome reporting (reporting bias)	Unclear risk	Study protocol was not found
Other biases (research allegiance, funding, confounds)	Unclear risk	Unclear whether there were allegiance or funding confounds)

Study Name: Grinstein‐Weiss, Russell, et al. (2017)		
Type of bias	Judgement	Support for judgement
Random sequence generation (selection bias)	Low risk	Random assignment to groups
Allocation concealment (selection bias)	Low risk	Concealment occurred
Blinding of participants and personnel (performance bias)	Low risk	Participants/personnel were blinded
Blinding of outcome assessment (detection bias)	Low risk	Evaluator role independent of intervention
Incomplete outcome data (attrition bias)	Low risk	Tx and comparison group had no attrition
Selective outcome reporting (reporting bias)	Unclear risk	Study protocol was not found
Other biases (research allegiance, funding, confounds)	Unclear risk	Unclear whether there were allegiance or funding confounds)

Study Name: Kim et al. (2005)		
Type of bias	Judgement	Support for judgement
Random sequence generation (selection bias)	High risk	Non‐random assignment to condition
Allocation concealment (selection bias)	Unclear risk	Allocation concealment unclear
Blinding of participants and personnel (performance bias)	High risk	No blinding of participants or personnel
Blinding of outcome assessment (detection bias)	Unclear risk	Evaluator role not reported
Incomplete outcome data (attrition bias)	High risk	Tx and comparison group had high attrition
Selective outcome reporting (reporting bias)	Unclear risk	Study protocol was not found
Other biases (research allegiance, funding, confounds)	Unclear risk	Unclear whether there were allegiance or funding confounds

Study Name: Learn$ave IDA Project		
Type of bias	Judgement	Support for judgement
Random sequence generation (selection bias)	Low risk	Random assignment to groups
Allocation concealment (selection bias)	Unclear risk	Allocation concealment procedures not described
Blinding of participants and personnel (performance bias)	Unclear risk	Not reported if participants/personnel were blinded
Blinding of outcome assessment (detection bias)	Low risk	Outcome assessors were blinded
Incomplete outcome data (attrition bias)	High risk	Both tx and control group had high attrition (>20%)
Selective outcome reporting (reporting bias)	Unclear risk	Study protocol was not found
Other biases (research allegiance, funding, confounds)	Unclear risk	Unclear whether there were allegiance or funding confounds

Study Name: Loke et al. (2016)		
Type of bias	Judgement	Support for judgement
Random sequence generation (selection bias)	High risk	Non‐random assignment to groups
Allocation concealment (selection bias)	Unclear risk	Allocation concealment procedures not described
Blinding of participants and personnel (performance bias)	Unclear risk	Not reported if participants/personnel were blinded
Blinding of outcome assessment were blinded	Low risk	Outcome assessors (detection bias)
Incomplete outcome data (attrition bias)	Unclear risk	Attrition rate not reported
Selective outcome reporting (reporting bias)	High risk	Only tx group outcomes reported
Other biases (research allegiance, funding, confounds)	High risk	One author designed the treatment intervention

Study Name: Lusardi et al. (2009)		
Type of bias	Judgement	Support for judgement
Random sequence generation (selection bias)	High risk	Non‐random assignment to groups
Allocation concealment (selection bias)	Unclear risk	Allocation concealment unclear
Blinding of participants and personnel (performance bias)	Unclear risk	Not reported if participants/personnel were blinded
Blinding of outcome assessment (detection bias)	Low risk	Assessors were blinded to group assignment
Incomplete outcome data (attrition bias)	Unclear risk	Attrition rates not reported
Selective outcome reporting (reporting bias)	Unclear risk	Study protocol was not found
Other biases (research allegiance, funding, confounds)	High risk	Authors designed the treatment intervention

Study Name: Michigan SEED (MI SEED) (2 sub‐studies)		
Type of bias	Judgement	Support for judgement
Random sequence generation (selection bias)	Low risk	Sites randomly assignment to groups
Allocation concealment (selection bias)	Low risk	Allocation concealment via site assignment
Blinding of participants and personnel (performance bias)	Unclear risk	Not reported if participants/personnel were blinded
Blinding of outcome assessment (detection bias)	Low risk	Outcome assessors were blinded
Incomplete outcome data (attrition bias)	Low risk	Low attrition
Selective outcome reporting (reporting bias)	Unclear risk	Study protocol was not found
Other biases (research allegiance, funding, confounds)	Unclear risk	Unclear whether there were allegiance or funding confounds)

Study Name: Modestino et al. (2019)		
Type of bias	Judgement	Support for judgement
Random sequence generation (selection bias)	Low risk	Random assignment to groups
Allocation concealment (selection bias)	Unclear risk	Concealment not reported
Blinding of participants and personnel (performance bias)	Unclear risk	Not reported if participants/personnel were blinded
Blinding of outcome assessment (detection bias)	Low risk	Evaluator role independent of intervention
Incomplete outcome data (attrition bias)	High risk	Tx and comparison group had high attrition
Selective outcome reporting (reporting bias)	Unclear risk	Study protocol was not found
Other biases (research allegiance, funding confounds)	Unclear risk	Unclear whether there were allegiance or funding confounds

Study Name: Moulton et al. (2015)		
Type of bias	Judgement	Support for judgement
Random sequence generation (selection bias)	Low risk	Random assignment to groups
Allocation concealment (selection bias)	Unclear risk	Concealment not reported
Blinding of participants and personnel (performance bias)	Unclear risk	Not reported if participants/personnel were blinded
Blinding of outcome assessment (detection bias)	Low risk	Evaluator role independent of intervention
Incomplete outcome data (attrition bias)	High risk	Tx group had high attrition
Selective outcome reporting (reporting bias)	Unclear risk	Study protocol was not found
Other biases (research allegiance, funding, confounds)	Unclear risk	Unclear whether there were allegiance or funding confounds)

Study Name: Osborne et al. (2016)		
Type of bias	Judgement	Support for judgement
Random sequence generation (selection bias)	High risk	Non‐random assignment to groups
Allocation concealment (selection bias)	Unclear risk	Allocation concealment procedures not reported
Blinding of participants and personnel (performance bias)	Unclear risk	Not reported if participants/personnel were blinded
Blinding of outcome assessment (detection bias)	Low risk	Outcome assessors were blinded
Incomplete outcome data (attrition bias)	Low risk	Low attrition
Selective outcome reporting (reporting bias)	Unclear risk	Study protocol was not found
Other biases (research allegiance, funding, confounds)	Unclear risk	Unclear whether there were allegiance or funding confounds

Study Name: Parks Opportunity Program (a.k.a. Assessing Financial Capabilities Outcomes) (3 sub‐studies)		
Type of bias	Judgement	Support for judgement
Random sequence generation (selection bias)	Low risk	Participants were assigned to groups based on month of their hire and work site.
Allocation concealment (selection bias)	Unclear risk	Allocation concealment not described
Blinding of participants and personnel (performance bias)	Unclear risk	Not reported if participants/personnel were blinded
Blinding of outcome assessment (detection bias)	Low risk	Outcome assessors were blinded
Incomplete outcome data (attrition bias)	High risk	Two studies reported different sample sizes
Selective outcome reporting (reporting bias)	High risk	Two studies reported different outcomes
Other biases (research allegiance, funding, confounds)	Unclear risk	Unclear whether there were allegiance or funding confounds

Study Name: Roder (2016)		
Type of bias	Judgement	Support for judgement
Random sequence generation (selection bias)	High risk	Non‐random assignment to groups
Allocation concealment (selection bias)	Unclear risk	Concealment not reported
Blinding of participants and personnel (performance bias)	Unclear risk	Not reported if participants/personnel were blinded
Blinding of outcome assessment (detection bias)	Low risk	Evaluator role independent of intervention
Incomplete outcome data (attrition bias)	High risk	Tx and comparison group had high attrition
Selective outcome reporting (reporting bias)	Unclear risk	Study protocol was not found
Other biases (research allegiance, funding, confounds)	Unclear risk	Unclear whether there were allegiance or funding confounds

Study Name: Roll et al. (2018)		
Type of bias	Judgement	Support for judgement
Random sequence generation (selection bias)	Low risk	Random assignment to groups
Allocation concealment (selection bias)	Low risk	Concealment occurred
Blinding of participants and personnel (performance bias)	Low risk	Participants/personnel were blinded
Blinding of outcome assessment (detection bias)	Low risk	Evaluator role independent of intervention
Incomplete outcome data (attrition bias)	Low risk	Tx and comparison group had no attrition
Selective outcome reporting (reporting bias)	Unclear risk	Study protocol was not found
Other biases (research allegiance, funding, confounds)	Unclear risk	Unclear whether there were allegiance or funding confounds)

Study Name: SEED OK (9 sub‐studies)		
Type of bias	Judgement	Support for judgement
Random sequence generation (selection bias)	Low risk	Random assignment to groups
Allocation concealment (selection bias)	Low risk	Allocation sequence was concealed
Blinding of participants and personnel (performance bias)	Unclear risk	Not reported if participants/personnel were blinded
Blinding of outcome assessment (detection bias)	Low risk	Outcome assessors were blinded
Incomplete outcome data (attrition bias)	Unclear risk	High attrition
Selective outcome reporting (reporting bias)	Unclear risk	Study protocol was not found
Other biases (research allegiance, funding, confounds)	High risk	Authors designed the treatment intervention

Study Name: Smith et al. (2017)		
Type of bias	Judgement	Support for judgement
Random sequence generation (selection bias)	Low risk	Random assignment to groups
Allocation concealment (selection bias)	Low risk	Allocation sequence was concealed
Blinding of participants and personnel (performance bias)	Unclear risk	Not reported if participants/personnel were blinded
Blinding of outcome assessment (detection bias)	Low risk	Assessors were blinded to group assignment
Incomplete outcome data (attrition bias)	High risk	Tx and comparison group had high attrition rate
Selective outcome reporting (reporting bias)	Unclear risk	Study protocol was not found
Other biases (research allegiance, funding, confounds)	Unclear risk	Unclear whether there were allegiance or funding confounds.

Study Name: Theodoes et al. (2016)		
Type of bias	Judgement	Support for judgement
Random sequence generation (selection bias)	Low risk	Random assignment to condition
Allocation concealment (selection bias)	Low risk	Allocation sequence was concealed
Blinding of participants and personnel (performance bias)	Unclear risk	Not reported if participants/personnel were blinded
Blinding of outcome assessment (detection bias)	Low risk	Assessors were blinded to group assignment
Incomplete outcome data (attrition bias)	Low risk	Low attrition
Selective outcome reporting (reporting bias)	Unclear risk	Study protocol was not found
Other biases (research allegiance, funding, confounds)	Unclear risk	Unclear whether there were allegiance or funding confounds

Study Name: Tufano (2014)		
Type of bias	Judgement	Support for Judgement
Random sequence generation (selection bias)	High risk	Non‐random assignment to groups
Allocation concealment (selection bias)	High risk	Concealment not reported
Blinding of participants and personnel (performance bias)	Unclear risk	Not reported if participants/personnel were blinded
Blinding of outcome assessment (detection bias)	Low risk	Evaluator role independent of intervention
Incomplete outcome data (attrition bias)	Low risk	Tx and comparison group had no attrition
Selective outcome reporting (reporting bias)	Unclear risk	Study protocol was not found
Other biases (research allegiance, funding, confounds)	Unclear risk	Unclear whether there were allegiance or funding confounds)

*Note*: Studies are presented in the order discussed in the narrative, grouped by study/program name, and presented in yearly ascending order.

Because the included studies did not have pre‐registered protocols, it is difficult to assess reporting bias for incomplete outcome data for all outcomes or selective outcome reporting. None of the included studies employed blinding of participants or personnel. With a few exceptions, there is a general absence of information related to the study authors' role in the interventions or potential bias stemming from study funding, thus we could not assess potential bias related to researcher allegiance or funding. Few studies had information about allocation concealment or blinding of outcome assessors for all outcomes.

Many studies did not report adequate data for review authors to calculate effect sizes.

Generally, the studies were conducted with randomly selected samples or convenience samples, and large sample sizes, and thus findings were generalizable. All study authors recognized study limitations and recommended that research on intervention effectiveness continue and that more rigorous research be conducted.

### 5.3 Synthesis of results

#### 5.3.1 Study intervention, design and outcomes by financial capability intervention strategy

As seen in Table 10, effects sizes and author‐reported outcomes or effect sizes varied across studies. The studies are reported here grouped by type of program (e.g., Matched Savings Accounts) and reported in yearly ascending order.

**Table 10:** Summary of effects sizes and author‐reported outcomes or effect sizes

Sub‐Study first author (year)	Study/program name	Primary study first author (year)	Effect size outcome type	Effect sizes	Author‐reported outcomes type	Author‐reported outcomes or effect sizes
	*Matched Savings Accounts*					
Lombe (2004)	ADD		Asset purchase‐ homeownership only (4 years post)—highly vulnerable subsample used	*d* = 0.11; 95% CI = −0.05 to 0.27		
Mills et al. (2006)	ADD		Asset purchase (4 years post)—subsample used	*d* = 0.06; 95% CI = −0.09 to 0.21 (asset purchase)		
Grinstein‐Weiss et al. (2008)	ADD		Asset purchase—homeownership ownership (4 years post) —renter subsample used	*d* = 0.17; 95% CI = −0.04 to 0.38		
			Debt (4 years post)	*d* = 0.15; 95% CI = −0.06 to 0.36		
Han et al. (2009)	ADD		Asset purchase—homeownership only (4 years post)—subsample used	*d* = −0.10; 95% CI = −0.14 to 0.13	Asset Value (4 years post)	For asset value, the study author reported a mean of $179.5 for the treatment group (*n* = 537), and $0 (*n* = 566) for the control group
					Debt (4 years post)	For debt, the study author reported a mean of $34,305.91 (*n* = 537) for the treatment group, and a mean of $29,681.14 for the control group (*n* = 566)
Huang (2010)	ADD		Asset purchase—homeownership only (4 years post)—subsample used	*d* = ‐0.10; 95% CI = −0.14 to 0.13	Asset Value (4 years post)	For asset value, the study author reported a mean of $179.5 for the treatment group (*n* = 537), and $0 (*n* = 566) for the control group
					Debt (4 years post)	For debt, the study author reported a mean of $34,305.91 (*n* = 537) for the treatment group, and a mean of $29,681.14 for the control group (*n* = 566)
Grinstein‐Weiss et al. (2012)	ADD		Asset purchase—postsecondary education enrolment (10 years post)	*d* = 0.16; 95% CI = 0.01 to 0.31		
Grinstein‐Weiss et al. (2013a)	ADD		Asset purchase—homeownership (10 years post)—subsample used	*d* = 0.02; 95% CI = −0.16 to 0.20		
Grinstein‐Weiss et al. (2015)	ADD		Saving rate for retirement savings (10 years post)	*d* = 0.05; 95% CI = −0.12 to 0.22	Retirement Savings Amount (10 years post)	For retirement savings amount, study authors reported a mean of $4332 for the treatment group saving amount (*n* = 311) and a mean of $5756 (*n* = 340) for the control group
Huang et al. (2016)	ADD		Asset purchase—homeownership (10 years post)—subsample used	*d* = 0.20; 95% CI = −0.04 to 0.44		
Mills et al., 2016	Assets for Independence		Asset Purchase (1 year post)	Homeownership *d* = 0.11, 95% CI = −0.33 to 0.54; Business ownership	Saving Amount	The study authors report no statistically significant differences at the *p* < 0.05 level
				*d* = −0.16, 95% CI = −0.58 to 0.26l		
				Postsecondary education		
				*d* = 0.08, 95% CI = −0.24 to 0.40		
Ratcliffe et al., 2019	Assets for Independence		Saving Amount (3 years post)	*d* = 0.18, 95% CI = −0.05 to 0.41		
			Asset Purchase (3 year post)	*d* = 0.10, 95% CI = −0.12 to 0.33		
Birkenmaier et al. (2012)	Credit Building in IDA Programs		Credit score (1‐year post)	*d* = 0.59; 95% CI = 0.28 to 0.90		
Birkenmaier et al. (2014b)	Credit Building in IDA Programs		Credit score (2 years post)	*d* = 0.56; 95% CI = 0.277 to 0.902		
Birkenmaier et al. (2014a)	Credit Building in IDA Programs				Credit Score (3 years post)	The study authors reported that results of the Wilcoxon Signed Rank Test indicated that the treatment group had significant increases in their median credit score between the first and last wave (*z* = −3.05, *p* < 0.05). The median score increased 30 points. The control group also had statistically significant increases overall (*z* = −2.03, *p* < 0.05), with a median increase of 21 points
	LearnSave IDA Project	Leckie et al. (2010a)	Saving Amount (4 years post)	*d* = 0; 95% CI = −0.108 to 0.108		
			Asset Value (4 years post)	*d* = 0.188; 95% CI = 0.083 to 0.294		
	*Child Development Accounts*					
Marks et al. (2009)	Michigan SEED		Budgeting (4 years post)	*d* = 0.236; 95% CI = 0.04 to 0.432		
			Savings Amount (4 years post)	*d* = 0.11; CI = −0.06 to 0.28		
Engelhardt et al. (2012)	Michigan SEED		Account Opening (4 years post)	*d* = 1.708; 95% CI = 1.446 to 1.969	Savings Amount (4 years post)	The study authors report that the mean savings in the treatment group was $912 (*n* = 302), and $288 (*n* = 298) for the control group
Huang et al. (2013)	SEED OK		Account Opening (3 years post) – outcome included participant‐owned account only	a *d* = 1.7; 95% CI = 1.38 to 2.03		
Nam et al. (2013)	SEED OK		Account Opening (18 months)	a *d* = 6.029; 95% CI = 4.93 to 7.128		
			Saving Amount (18 months)	*d* = 2.37 95% CI = 2.27 to 2.46		
Beverly et al. (2014)	SEED OK				Saving Amount (30 months)	Study authors reported that the mean savings for the treatment group was $1130 (*n* = 1358) and the mean savings for the control group was $75.7 (*n* = 1346)
Beverly, Kim, et al. (2015)	SEED OK				Saving Amount (3 years post)	The study authors reported that the mean saving amount for the treatment group was $1129.85 (*n* = 1353) and the mean saving amount for the control group was $75.74 (*n* = 1345)
Wikoff et al. (2015)	SEED OK		Account Opening (5 years post)	a *d* = 1.71; 95% CI = 1.38 to 2.03		
Huang et al. (2015)	SEED OK		Account Opening (6 years post)	a *d* = 1.63; 95% CI = 1.33 to 1.93	Saving Amount (6 years post)	The study authors reported that the mean saving amount for the treatment group was $153.71 (*n* = 1358) and the mean saving amount for the control group was $31.96 (*n* = 1346)
					Asset Value (6 years post)	The study authors reported that the mean asset value for the treatment group was $1,605.07 (*n* = 1358), while the mean asset value for the control group was $50 (*n* = 1346)
Clancy et al. (2016)	SEED OK		Account Opening (7 years post)		Saving Amount (7 years post)	The study authors reported that the mean saving amount for the treatment group was $1851 (*n* = 1358) and the mean saving amount for the control group was $322.8 (*n* = 1346)
Huang et al. (2017)	SEED OK		Account Opening (5 years post)	a *d* = 1.62; 95% CI = 1.31 to 1.92	Saving Amount (5 years post)	The study authors reported that the mean saving amount for the treatment group was $152.93 (*n* = 1343) and the mean saving amount for the control group was $32.18 (*n* = 1334)
Huang et al. (2019)	SEED OK		Account Opening (7 years post)	a *d* = 1.524; 95% CI = 0.149 to 2.898		
			Asset Value (7 years post)	*d* = 3.153; 95% CI = 2.868 to 3.437		
	Osborne et al. (2016)	Osborne et al. (2016)			Account Opening (1 week post)	Study authors report that 13.8% of the treatment group opened accounts, and the mean savings for the treatment group was $135.06
					Saving Amount (1 week post)	
	*Youth Bank Accounts*					
	Loke et al. (2016)	Loke et al. (2016)			Saving Amount (post timing not reported)	All of the participants in the treatment group saved some portion of their income, with total savings ranging from $9 to $2268
	*Retirement Accounts*					
	Duflo et al. (2006)	Duflo et al. (2006)	Account Opening (end of tx)	OR = 2.79; CI = 2.28 to 3.41 (Treatment Group 1)		
				OR = 4.67; CI = 3.87 to 5.64 (Treatment Group 2)		
	Lusardi et al. (2009)	Lusardi et al. (2009)			Account Opening (end of tx)	The intervention resulted in a 56.2% increase in account opening within 30 days of the intervention compared to the comparison group
	Goda et al. (2012)	Goda et al. (2012)	Saving Amount (end of tx)	Treatment group 1, *d* = −0.01; 95% CI = −0.06 to 0.03; treatment group 2, *d* = −0.02; 95% CI = −0.02 to 0.07; and treatment group 3, *d* = −0.00; 95%, CI = −0.04 to 0.04		
			Retirement Saving Rate (end of tx)	Treatment group 1, *d* = −0.02; 95% CI = ‐0.06 to 0.02; treatment group 2, *d* = 0.02, 95% CI = −0.02 to 0.07; and treatment group 3, *d* = 0.03; 95%, CI = −0.01 to 0.07		
	Collins et al., (2016)	Collins et al., (2016)			Account Opening (4 to 11 months post)	The study authors reported that the intervention increased account opening by 6%, and the rate of creation of a budget by almost 6%. The intervention increased the retirement savings participation by 3.7%–3.8%, the retirement savings rate by 40.4%, and emergency savings by 3.8%
					Budgeting (4 to 11 months post)	
					Retirement Savings (4 to 11 months post)	
					Retirement Savings Rate (4 to 11 months post)	
	*Pre‐purchase Home Buying Education*					
	Smith et al. (2017)	Smith et al. (2017)			Debt (5 years post)	Treatment group participants who were homeowners saw a statistically significant decrease in debt compared to the control group homeowners. There were no statistically significant differences on debt or saving amount between non‐home owning treatment and control participants. Regarding credit scores, the study authors reported no significant difference between the treatment and control groups
					Saving Amount (5 years post)	
					Credit Score (5 years post)	
	*Adult Financial Education, Counselling and Coaching*					
Gons (2013)	Parks Opportunity Program (a.k.a. Assessing Financial Capabilities Outcome Pilot)				Credit Scores (9 months post)	The study author reports that study participants had 'better outcomes with respect to credit scores, lower levels of revolving debt and fewer accounts in collections than those who did not receive financial counselling'
					Debt (9 months post)	
Wiedrich et al. (2014)	Parks Opportunity Program (a.k.a. Assessing Financial Capabilities Outcome Pilot)		Credit Scores (6 and 12 months post)	*d* = 0.24; 95% CI = 0.06 to 0.42		
				*d* = 0.15; 95% CI = −0.40 to 0.33		
Collins et al. (2019)	Parks Opportunity Program (a.k.a. Assessing Financial Capabilities Outcome Pilot)				Credit Scores (6 and 12 months post)	From Collins 2014 (duplicate study): Study authors report that in the first six‐months post‐intervention, the treatment groups experience 'some increase' in credit scores. However, during the 6–12 month period, the control group also experienced increased in credit scores and caught up to the treatment group. At the 12‐month mark, there was no measurable effect of counselling on credit scores
					Debt (6 and 12 months post)	Study authors report that the intervention led to 13% reduction in past debt due over a 12‐month period for the treatment group
					Saving Amount (6 and 12 months post)	For the outcome of savings amount, the treatment group experienced reduced bank balances in the 6–12 month post‐intervention period, which was not statistically significant. Post‐intervention, the mean bank balance for the treatment group was $41 less
	Kim, Garman, Sorhaindo (2005)	Kim, Garman, Sorhaindo (2005)	Budgeting (18 months post)	OR = 3.14; CI = 1.55 to 6.39		
			Debt (18 months post)	OR = 6.4; CI = 2.13 to 19.25		
	Moulton et al. (2015)	Moulton et al. (2015)	Saving Amount (12 months post)	*d* = 0.127, 95% CI = −0.081 to 0.334		
			Credit Scores (12 months post)	*d* = −0.038, 95% CI = −0.245 to 0.17		
	Theodos et al. (2016)	Theodos et al. (2016)			Credit Score (6 months post)	For credit score outcomes, study authors report that the mean of the treatment group post‐intervention was 702.42 and 703.7 for the control group. For the debt outcome, study authors report that the mean debt for the treatment group was $4926, and $5118 for the control group
					Debt (6 months post)	
	Roder (2016)	Roder (2016)			Credit score (2 years post)	The study authors report a Hedges *g* effect size of 0.01. The study author did not report the confidence intervals or sufficient information for the review authors to calculate an effect size
	Modestino et al. (2019)	Modestino et al. (2019)			Credit Scores (6 and 18 months post)	Study authors reported significant increase in credit scores (18 points) during the first 6 months of the program among those who initially had a credit file at baseline compared to the control group
	*Tax Refund Saving and Investment*					
	Grinstein‐Weiss et al. (2015) Refund to Savings	Grinstein‐Weiss et al. (2015)			Saving Amount (end of tx)	Study authors report that the proportion of filers who deposited any tax refund into a savings vehicle was statistically higher in every treatment condition than in the control group
	Grinstein‐Weiss, Cryer, et al. (2017) Refund to Savings	Grinstein‐Weiss, Cryer, et al. (2017)			Saving Amount	Study authors reported that treatment participants in three groups saved more than the control group: (a) the Choice Architecture condition (*d* = 0.22, *p* = 0.009); (b) the Savings Emphasized Twice condition (*d* = 0.29, *p* < 0.001); and (c) the Savings‐Single Click condition (*d* = 0.36, *p* < 0.001)
					Saving Rate (end of tx)	Study authors report that 13% of treatment group saved compared to 8% of control participants
	Grinstein‐Weiss, Russell, et al. (2017) Refund to Savings	Grinstein‐Weiss, Russell, et al. (2017)			Saving Amount (end of tx)	On average, the control group saved $73 and the treatment group saved $93. Average savings by the eight treatment groups ranged from $86 to $107, and all of those groups saved significantly more than the control group did
	Roll et al. (2018) Refund to Savings	Roll et al. (2018)			Saving Amount (end of tx)	Study author reported statistically significant difference on rate of depositing any refund and dollar amounts
	Tufano (2011)	Tufano (2011)			Saving Amount (end of tx)	Study authors report that treatment group members saved an average of $28.21, compared to a control group mean of $12.95
					Purchase Asset (end of tx)	Study authors report that 7% of treatment participants purchased an asset compared to 0.74% of control group participants

*Note*: Studies are presented in the order discussed in the narrative, grouped by study/program name, and presented in yearly ascending order.

^a^ Participant owned account.

#### 5.3.2 Matched Savings Accounts

Study authors produced several reports/sub‐studies as a result of three large longitudinal studies focused on matched savings accounts, ‘The American Dream Demonstration Project’ (ADD), the ‘Credit Building in Individual Development Account (IDA) Programs’, and the ‘Assets for Independence’ program (also known as the ‘Building Savings for Success’ program), all of which are described below. In addition, an unrelated matched savings account study (Leckie et al., 2010a) is also discussed in this section.

##### ADD

ADD was the first large‐scale test of Individual Development Accounts (IDAs) in the US. IDAs are restricted matched savings accounts for specific asset‐building purposes, such as home purchase and renovation, postsecondary education, and microenterprise for low‐income employed populations. Participants were low‐income, and employed. Participants of IDA programs were offered a match (i.e., participant savings were matched with incentive funds (i.e., money from the program) of one‐to‐one or higher (i.e., 1 to 2, so that for every dollar they contributed to a special bank account set up for this purpose, they received $2 in incentive money at the end of the program) for their savings, in combination with high‐intensity (face‐to‐face) financial education classes and social support, such as peer group meetings, case management and counselling. The intervention was delivered in community‐based non‐profit agencies, and lasted 3–4r years, depending on participants completed their savings. At the end of the study period, participants could place remaining money in their IDA account into a retirement savings account (Roth IRA) and receive a match into the account. The ADD studies measured a variety of outcomes periodically over as long as 10 years.

ADD had 2350 participants in 14 IDA programs hosted by 13 community organizations around the US. One site in Tulsa Oklahoma (US) was a randomized control trial (*n* = 1103), while the other 13 used a quasi‐experimental design. The Tulsa Oklahoma ADD site used individual randomized assignment to condition and employed various data collection methods to evaluate the project, including surveys, administrative data, case studies and in‐depth interviews. The following nine sub‐studies (Grinstein‐Weiss et al., 2008, 2012, 2013a, 2013b, 2015; Han et al., 2009; Huang, 2010; Huang et al., 2016; Lombe, 2004; Mills et al., 2006) all used data from the Tulsa Oklahoma site, which was the only site that used a randomized control trial research design. The studies used various data sources, time frames, and sub‐samples from the total sample of *n* = 2350 to examine a variety of financial outcomes, including savings amounts and asset value, and behavioural outcomes including account opening, saving rates, and asset purchase.

For all studies/reports of the ADD project discussed below, the role of the evaluator was completely independent from the treatment; non‐profit staff members delivered the intervention after training through periodic contact with participants. The number of contacts and whether the intervention was manualized was not reported. Fidelity assessment was not reported. Unless noted otherwise, the control group received nothing during the intervention and agreed to abstain from participating in any other matched savings or homeownership program provided by the community non‐profit agency providing the intervention for the 4‐year intervention period. None of the studies reported matching the treatment and control groups, and few reported results of statistical pre‐test comparison on demographics between the treatment and control groups. Some of the studies reported results of statistical comparison on differences on outcomes. The predominant race for all of the studies was White. For all of these studies, the risk of bias is high for incomplete outcome data for all outcomes (because both the treatment and control groups had high attrition (over 20% reported) and other biases (researcher or funding bias) because an author designed the intervention. Risk of bias is unclear for sequence generation, blinding of participants and personnel, selective outcome reporting, and judged as low for blinding of outcome assessors, and allocation concealment. The studies are reported from the earliest to the most recent studies.

Lombe (2004) studied *n* = 736 of the *n* = 2350 total sample (treatment *n* = 361, control *n* = 375) at 4 years after enrolment in the IDA program. This subsample was created by inclusion of only participants that met at least three of five criteria for vulnerability (i.e., education level, children under age 17, marital status, race, and welfare status) and participated in three waves of data collection. Substantial attrition was observed between the time the original sample was recruited and the wave of data collected that was used for this study due to dropout or missing data at wave III. The mean age of participants was 36.5 years, with 17% of the participants being male. Using interview, survey, and administrative data, Lombe evaluated the extent to which treatment group participants purchased an asset and the value of the assets. There was a small but not a statistically significant effect of the intervention on purchase of an asset (i.e., homeownership) (*d* = 0.11, 95% CI = −0.05 to 0.27) at 4 years post‐enrolment. The study author measured asset value, but did not report sufficient data to calculate an effect size. The study is assessed as unclear for bias for substantial attrition.

Mills et al. (2006) examined effects of ADD at 18 months and 4 years post enrolment on asset purchase. Their analyses included 840 of the 2350 total sample (treatment *n* = 412, control *n* = 428). The mean age of participants was 36.3 years, with 20% of the participants being male. The smaller sample size at the 18 months and 4 years post enrolment was due to attrition (drop out and missing data). They found small, but non‐statistically significant effects on purchased assets (*d* = 0.06, 95% CI = −0.09 to 0.21). Of note is that the study authors examined demographic differences between groups and found statistically significant differences between the treatment and control groups on demographics. Given the substantial attrition and differences in demographics between groups, the review authors judged this study to be at high risk of bias.

Grinstein‐Weiss et al. (2008) examined effects of ADD 4 years post enrolment on homeownership and debt. Their sample included 475 of the original 2350 sample (treatment *n* = 228, control *n* = 247). The sample included only subjects that completed wave III data and were renters at baseline. Control group members received nothing, but were also not barred from receiving homeownership counselling from other area providers besides the intervention provider. The mean age of participants was 34.83 years, and 17% of the sample was male. The study authors reported statistically significant differences between groups on demographic variables (number of children and income). The effect sizes for homeownership (*d* = 0.17, 95% CI = −0.04 to 0.38) and lowered debt (*d* = 0.15, 95% CI = −0.06 to 0.36) were small and not statistically significant. Given the high attrition and differences between the treatment and comparison groups on demographic variables, we judged this study to be at high risk of bias.

Han et al. (2009) examined effects of ADD at 4 years after enrolment to assess whether participants accumulated assets beyond matched savings. The analysis only included 840 participants of the original 2350 total sample (treatment *n* = 412, control *n* = 428) due to attrition. Study authors reported statistically significant differences between groups on demographic variables, indicating that those who left the study were statistically significantly different than those who remained in the study. The mean age of participants in this study was 40.67 years, and 20.22% of the participants were male. Using interview and survey data, they evaluated the value of total participant assets beyond just the assets purchased through the IDA program, including liquid assets, other financial assets, retirement savings value, and value of real estate and nonfinancial assets (i.e., car). The effect (*d* = 0.06, 95% CI = −0.07 to 0.20) was small and not statistically significant. Given the substantial attrition and differences in demographics between groups, we judged this study to be at high risk of bias.

Huang (2010) studied *n* = 1103 of the *n* = 2350 total sample (treatment *n* = 537, control *n* = 566) at 18 months and 48 months after enrolment in the IDA program to examine the effects of IDA accounts on household wealth of participants. Only participants that participated in wave III data were included. No statistical results comparing pre‐test differences on outcomes were reported; however, statistically significant pre‐test differences on demographics of the treatment and control group were found. The mean age of participants in this study was 35.82 years, and 21.58% of the participants were male. Using interview and survey data, Huang evaluated the extent to which treatment group participants purchased an asset (homeownership), value of the asset and debt. The effect of purchasing an asset (homeownership) at 48 months (wave III) was small and not statistically significant (*d* = −0.1, 95% CI = −0.14 to 0.13). The study author measured asset value and debt, but did not report sufficient data to calculate an effect size. For asset value, the study author reported a mean of $179.5 for the treatment group (*n* = 537), and $0 (*n* = 566) for the control group. For debt, the study author reported a mean of $34,305.91 (*n* = 537) for the treatment group, and a mean of $29,681.14 for the control group (*n* = 566).

Grinstein‐Weiss et al., (2012, 2013a, 2015), using interview and survey data, reported outcomes of IDA at 10 years post enrolment in three different reports for three outcomes: enroled in post‐secondary education, baseline renters became homeowners, and retirement savings of participants. Samples used for this study were those who participated in wave IV (since baseline) and did not have missing data on the analysis covariates. For post‐secondary enrolment (Grinstein‐Weiss et al., 2012), 824 (treatment *n* = 392, control *n* = 432) of the original 2350 participants were included in the analysis. The mean age of participants was 24–45 years, and 20% of the sample was male. They found small positive statistically significant effects on post‐secondary education enrolment (*d* = 0.16, 95% CI = 0.01 to 0.31). In Grinstein‐Weiss et al. (2013a), the effects of the IDA on home purchase for baseline renters (*n* = 604 of the original 2,350 sample, treatment n = 298, control n = 306) was small and not statistically significant (*d* = 0.02, 95% CI = −0.16 to 0.20). The mean age of participants was 34.25 years, and gender distribution of the sample was not reported. The sample for Grinstein et al. (2015) also excluded participants who were over age 55 at baseline (and past retirement age at wave IV). The mean age of their participants was 45 years, and 20% of the participants were male. The effects of the IDA on the amount of savings rate for retirement savings (*n* = 660 of the original 2350 sample) was small and not statistically significant (*d* = 0.05, 95% CI = −0.12 to 0.22). We could not calculate the effect size for savings amount for retirement savings, as the study authors did not report sufficient information. Study authors reported a mean of $4332 for the treatment group saving amount (*n* = 311) and a mean of $5756 (*n* = 340) for the control group. It must be noted that the study authors did examine demographic differences between groups and found statistically significant differences on demographics between the treatment and control groups. Given the differences between groups on demographics and the high attrition between the original study enrolment and the sample sizes used for analysis of these outcomes at 10 years post‐enrolment, risk of bias was judged as high for these studies/outcomes.

Huang et al. (2016) examined effects of ADD on homeownership with 336 participants with disabilities from the original sample of 2350 (treatment *n* = 174, control n = 162) at 10 years after enrolment in the IDA program. The mean age of participants was 38 years, and 20% of the sample was male. The effect of the intervention on asset purchase (homeownership) for participants with disabilities was small and not statistically significant (*d* = 0.20, 95% CI = −0.04 to 0.44).

##### Assets for Independence (also known as ‘Building Saving for Success’)

Assets for Independence is a longitudinal study of the Individual Development Accounts (IDA) intervention implemented in two community‐based sites: at a community college in Albuquerque New Mexico and a community‐based nonprofit organization in Los Angles California. Participants received high‐intensity (face‐to‐face) financial education classes and a matched savings account to help build financial assets. Participants could use their matched savings to purchase an asset, such as a home, a vehicle, or post‐secondary education. Treatment group participants were offered a match (i.e., participant savings were matched with incentive funds (i.e., money from the program). One site offered a match of 4‐to‐1, and the second offered a 2.5‐to‐1 match.

Researchers used an experimental design to study financial outcomes. Participants were assigned individually to their condition. The treatment and control groups were not matched. Study participants were predominantly under age 40. Like other IDA programs, participants were low‐ and moderate‐income and employed. The predominant race was Hispanic. The role of the evaluator was independent of treatment. Instead, college and non‐profit staff delivered the intervention, and had periodic contact with study participants, although the exact number is unreported. Sub‐study authors also did not report whether the intervention was manualized or fidelity measured. At one site, control group members were those participants that learned about the program, and had access to the same financial education course and credit counselling, but did not have access to the matched accounts. At the second site, control group members received referrals to relevant financial services only. Both sub‐studies measured the financial outcomes from baseline survey, administrative data, and follow‐up surveys. Both sub‐studies reported saving amount, and purchase asset outcomes at different times. The intervention lasted a minimum of 6 months and as long as 2 years, depending on how quickly participants completed their savings goals. The review authors found that the risk of bias is high for include outcome data. The risk of bias is low for sequence generation, other (research allegiance), blinding of participants and personnel, and blinding of outcome assessors. The risk of bias is unknown for selective outcome reporting and allocation concealment.

Mills et al. (2016) studied outcomes after 1 year using baseline survey data. For their study, there were pre‐test differences in the groups on an outcome (liquid assets), but no statistical comparisons were made for pre‐test different on demographics. 30% of their sample was male. The treatment and control groups had high attrition (>20%). There was a total of *n* = 807 subjects (treatment group *n* = 407, and control group *n* = 400). Study authors reported saving amount and purchase asset outcomes 12 months post‐baseline. For saving amount, authors did not report sufficient data to calculate effect sizes on liquid assets amount, but reported no statistically significant differences at the *p* < 0.05 level on those outcomes. For homeownership, business ownership and education, no statistically significant effects were found (*d* = 0.11, 95% CI = −0.33 to −0.54; *d* = −0.16, 95% CI = −0.58 to 0.261; *d* = 0.08, 95% CI = −0.24 to 0.40, respectively). At the third‐year follow‐up, a small but not statistically significant effect was found on asset ownership (*d* = 0.10, 95% CI = −0.12 to 0.33) (Ratcliffe et al., 2019).

Ratcliffe et al. (2019) studied outcomes after 3 years using data from the baseline survey and a follow‐up survey 3 years after study enrolment. They found pre‐test statistical differences on most demographics, such as age, race, and education level, however, statistical controls were used in the analysis. 28.2% of their sample was male. Whether treatment or control group had high attrition was unreported. The total sample size was *n* = 621 (treatment *n* = 326; control *n* = 295). Study authors reported saving amount and purchase asset outcomes 3 years post‐baseline. For saving amount, a small but not statistically significant effect was found for liquid assets amount (*d* = 0.18, 95% CI = −0.05 to 0.41). A small but not statistically significant effect was found on asset ownership (*d* = 0.10, 95% CI = −0.12 to 0.33).

##### Credit Building in Individual Development Account (IDA) Programs

The second large study about matched saving accounts is the ‘Credit Building in Individual Development Account (IDA) Programs’, for which three sub‐studies were produced. Researchers used a quasi‐experimental design to study credit outcomes of the Individual Development Account (IDA) (matched savings account) intervention, identical to the ADD program described earlier, at three community‐based agencies. Like the ADD study, participants were low‐income and employed. Participants (*n* = 165) could use their matched savings to purchase a home, a vehicle, post‐secondary education or invest in home improvements or a microenterprise. The comparison group were those participants that learned about the program, but did not enrol in financial education or subsequent program steps. The treatment and comparison groups were not matched. All three sub‐studies measured the financial outcome of credit scores from baseline survey and administrative data.

For all studies discussed below, the unit of assignment to condition was individual. The treatment and comparison groups were not matched. High‐intensity (face‐to‐face, classroom‐based, multiple session) financial education was delivered in the intervention. The predominant race of participants was African American, and the mean age was 31–40 years old. The role of the evaluator was independent from the treatment; the non‐profit staff delivered the intervention through periodic contact with participants. Whether the intervention was manualized or fidelity was assessed was not reported. Neither the treatment nor the control group had high attrition (under 20% reported). The control group received nothing during the intervention. The three studies (Birkenmaier et al., 2012, 2014a, 2014b) used the same sample and studied the same outcome (financial credit score) using the same measure (the individual consumer credit report) at three different time periods.

Overall, the risk of bias is high for these studies for sequence generation and unclear for allocation concealment, and blinding of participants and personnel. The risk of bias is low for blinding of outcome assessors, incomplete outcome data, selective outcome reporting, and other (i.e., researcher or funding allegiance bias).

The first sub‐study, Birkenmaier et al. (2012), measured outcomes 1 year after baseline measure on the treatment (*n* = 78) and comparison group (*n* = 87), for a total sample size of *n* = 165. The study authors did not compare pre‐test differences on outcomes and found no statistically significant differences on demographics. The percentage of males in the study was not reported. The effect of the intervention on credit scores was moderate and statistically significant (*d* = 0.591, 95% CI = 0.279 to 0.903).

The second sub‐study, Birkenmaier et al. (2014b), measured outcomes 2 years after baseline with a treatment (*n* = 79) and comparison group (*n* = 85), for a total sample size of n = 164. The study authors found statistically significant pre‐test differences on outcomes, favouring the treatment group, but did not make comparisons on pre‐test demographics. Thirteen percent of the sample was reported as male. The effect of the intervention on credit scores was moderate and statistically significant (*d* = 0.56, 95% CI = 0.277 to 0.902).

In the third sub‐study, Birkenmaier et al. (2014a) measured outcomes 3 years after baseline measure on the treatment (*n* = 79) and comparison groups (*n* = 85), for a total sample of *n* = 164. The study authors did not compare pre‐test differences on outcomes or demographics. Thirteen percent of the sample was reported as male. We could not calculate the effect size for savings amount or credit score, as the study authors did not report sufficient information. The study authors reported that results of the Wilcoxon Signed Rank Test indicated that the treatment group had significant increases in their median credit score between the first and last wave (*z* = −3.05, *p* < 0.05). The median score increased 30 points. The control group also had statistically significant increases overall (*z* = −2.03, *p* < 0.05), with a median increase of 21 points.

Leckie et al. (2010a) used a randomized control trial to measure outcomes from ‘LearnSave Individual Development Accounts Project’, a Canadian matched savings account program. Study authors used a randomized control trial to explore two different methods of delivering IDAs to low‐ and moderate‐income population over a 4‐year period in 10 communities (large and medium‐size urban and rural communities with varied economic and educational environments across seven Canadian provinces). Three of the 10 sites used experimental design, and project effects are only reported from those three sites. Participants were individually randomly assigned to one of three groups. The first treatment group had high‐intensity financial education (face‐to‐face multi‐session classes), case management, and a restricted savings account, and the second treatment group was provided a matched savings account without financial education or case management. The comparison group received nothing during the intervention. Leckie et al. (2010a) studied a total of *n* = 3583 (treatment group 1 *n* = 1195, treatment group 2 *n* = 1193, control group *n* = 1195) at 48–52 months after baseline. Using survey data, the study authors measured the outcomes of saving amount and asset value. Using only outcome data from the control group and the first treatment group that received both financial education and a matched savings account, the effects were small, but not statistically significant on saving amount (*d* = 0, 95% CI = −0.108 to 0.108), and asset value (*d* = 0.188, 95% CI = 0.083 to 0.294). In this study, no statistical pre‐test comparisons were made on outcomes, but statistically significant differences were found on demographics between the two groups. The mean age of participants was 33–34, and the predominant race of participants was not reported. Forty‐four percent of the participants were male. Participants were low‐ and moderate‐income. The role of the evaluator was completely independent from the treatment; the non‐profit staff delivered the intervention through periodic contact with participants. Whether the intervention was manualized or fidelity was assessed was not reported. Both the treatment and the comparison group had high attrition (over 20% reported); thus, the risk of bias was assessed to be high for incomplete outcome data. Risk of bias was assessed to be low for the bias domains of sequence generation, blinding of outcome assessors and allocation concealment. The risk of bias was assessed to be unclear for blinding of participants and personnel, selective outcome reporting and other.

#### 5.3.3 CDAs

##### Michigan SEED (MI SEED)

One of the first Children's Development Account programs occurred in the (US) Detroit Michigan area, called ‘Michigan Saving for Education, Entrepreneurship and Down payment (SEED)’ experiment (or MI SEED). For this randomized control study, low‐income parents of children enroled in 14 Head Start programs, a national preschool program for children, were encouraged to open a CDA for their children's post‐secondary education (i.e., a Michigan 529 College Savings Account). These accounts were parent, rather than state, owned. For the treatment group, there was a $1000 automatic deposit into the account, and parent contributions were matched one‐to‐one on the first $1200 of contributions. Treatment participants were also offered low‐intensity financial education (written materials about education savings) and case management. The intervention lasted three‐4 years.

To create the treatment and control groups, Head Start centres were matched in pairs based on enrolment and key demographic variables. One Head Start center of each of the pairs was randomly assigned to the treatment group, so there were seven centres in each of the treatment and control groups. (Note that Engelhardt et al. (2012) describes the study as a randomized control trial, and Marks et al. (2012) describe the study as a quasi‐experimental design. Based on the description, this review codes this large longitudinal study project as a randomized control trial). Control group members received nothing, but were not barred from opening a 529 account on their own. Participants completed a baseline survey and follow‐up survey 4 years post‐baseline. Sub‐study authors measured the financial outcome of savings amounts and behavioural outcome of account opening and budgeting. Study participants were low‐income. Non‐profit staff delivered the intervention. The sub‐study authors did not report the number of sessions, whether the intervention was manualized, or whether fidelity was assessed.

The following two sub‐studies, Marks et al. (2009) and Engelhardt et al. (2012) examined the financial outcome of savings amounts and behavioural outcome of account opening and budgeting. Overall, risk of bias was assessed as low for both of these studies in bias domains of sequence generation, allocation concealment, and blinding of outcome assessors. The risk of bias was assessed to be unclear for blinding of participants and personnel, incomplete outcome data, selective outcome reporting and other.

Marks et al. (2009) studied a total sample n = 686, (treatment group *n* = 338, control group *n* = 348), also at 4 years post baseline. In this study, statistical pre‐test differences were found for the outcomes and demographics. The treatment and control group participants were matched based on demographics. The mean age of the parent participants was 34.4 years old, and 9.2% of the sample was reported as male. The predominant race of the participants was White. The role of the evaluator was independent of the treatment. The frequency of contact was reported as periodic. The attrition for both the treatment and control group was under 20%. Using baseline and follow‐up survey data, they analysed savings amounts and budgeting outcomes. The effects of the intervention on budgeting were small, but statistically significant (*d* = 0.236, 95% CI = 0.04 to 0.432). Effects on savings was also small and not statistically significant (*d* = 0.11, 95% CI = −0.06 to 0.28).

Engelhardt et al. (2012) studied a total sample n = 600 (treatment group *n* = 302, and control group *n* = 298) at 4 years post treatment. In this study, the full sample was 790, and they experienced 14% attrition from baseline. The sample was further reduced by respondents who did not provide outcomes measure data. The separate attrition rates of the treatment and control group were not reported. No statistical pre‐test comparisons were made on differences in outcomes, and no statistically significant pre‐test differences on demographics were found. The mean age of the parent participants was 30 years old. The predominant race of the participants was not reported, nor was the gender distribution. The role of the evaluator was not reported. The frequency of contact was not reported. Using baseline and follow‐up survey data, they analysed account opening and saving amounts of participants. The effects of the intervention on account opening were large and statistically significant (*d* = 1.708, 95% CI = 1.446 to 1.969); treatment participants opened more savings accounts than the control group. We could not calculate the effect size for savings amount, as the study authors did not report sufficient information. The study authors report that the mean savings in the treatment group was $912 (*n* = 302), and $288 (*n* = 298) for the control group.

##### SEED OK

The Saving for Education, Entrepreneurship and Down payment (SEED) OK experiment is a large randomized test of universal, automatic child savings accounts conducted in Oklahoma (OK). Using birth records, the experiment randomly selected 7115 mothers and their children from all infants born in the US state of Oklahoma during certain periods in 2007 (regardless of family income). Infants and their mothers were individually randomly assigned to the treatment group or the control group after mothers completed baseline interviews (*n* = 2704). Treatment group children (*n* = 1358) received a *state‐owned* CDA, an Oklahoma 529 College Savings Plan account (OK 529) with an initial deposit of $1000, which was automatically opened for them. When the treatment children are ready, the funds that accumulate in the account will be sent directly to the postsecondary educational institution of their choosing by the state. The intervention also includes low‐intensity financial education (i.e., mailed materials), a progressive savings match, and a time‐limited (4 years) $100 account‐opening incentive for treatment mothers to open a second, *participant‐owned* Oklahoma 529 College Savings account in which they could save directly for their children's education. Low‐ and moderate‐income treatment group mothers were also eligible for savings *matches on deposits into their participant‐owned* 529 accounts. After a baseline telephone survey, all intervention contact with treatment group occurred through the mail. Sixty mothers completed extended interviews 2–3 years later, and 2272 mothers completed a telephone survey 4 years after baseline. Administrative data (state birth records and OK 529 account records) were also used. Control group infants (*n* = 1346) and mothers received nothing, but were not barred from opening an individual OK 529 account.

The predominant race of participants was White. Participants were all income levels. The sub‐study authors also served as the evaluator because the study authors delivered the mailed financial education materials and arranged for the state to provide birth records and open accounts. Periodic contact was made with the participants. Whether the intervention was manualized or fidelity was assessed was not reported. Neither the treatment nor the control group had high attrition (under 20% reported). Unless otherwise noted, the treatment and comparison group were not matched.

The following nine sub‐studies (Beverly et al., 2014; Beverly, Kim, et al., 2015; Beverly, Clancy, et al., 2015; Huang et al., 2013; Nam et al., 2013; Clancy et al., 2016; Huang et al., 2015; Huang et al., 2017; Huang et al., 2019; Wikoff et al., 2015) used various research methods, time frames, types of 529 accounts, and populations within the *n* = 2704 total sample to study the financial outcome of savings amounts and behavioural outcome of account opening from participants participating in this study. Overall, risk of bias was assessed as low for these studies in bias domains of sequence generation, allocation concealment, and blinding of outcome assessors. The risk of bias was assessed to be unclear for blinding of participants and personnel, incomplete outcome data, selective outcome reporting and other. Because some sub‐studies selected sub‐samples, some bias may be introduced, depending on how those subsamples were selected.

Huang et al. (2013) examined effects at 3 years post‐baseline for participant‐owned accounts. Their sample included *n* = 2675 (*n* = 1341 treatment group, and *n* = 1334 control group) participants. In this study, no statistical pre‐test differences were found on outcomes or demographics. The mean age of the parent participants was 26, and the mean age of the infants was not reported, although the infants would have been around the age of three at the time of this study. About half (53.13%) of the children were male. Using administrative data and baseline telephone survey, they analysed the extent to which participants opened participant‐owned accounts. The effects of the intervention on participant account opening were large (*d* = 1.70, 95% CI = 1.38 to 2.03).

Nam et al. (2013) examined effects at 18 months post‐baseline for participant‐ and state‐owned accounts. Their sample included *n* = 2670 (*n* = 1340 treatment group, and *n* = 1330 control group) participants. In this study, no statistical pre‐test differences were found on outcomes or demographics. The mean age of the parent participants was 25–34 years old, and the mean age of the infants was not reported, although the infants would have been around the age of 18 months. About half (53.09%) of the children were male. Using administrative data and baseline telephone survey, they analysed the extent to which participants opened participant and state‐owned accounts and saving accounts. The effect of the intervention on participant account opening was large (*d* = 6.029, 95% CI = 4.93 to 7.128). The effect of the intervention on saving amount was large (*d* = 2.37, 95% CI = 2.27 to 2.46).

Beverly et al. (2014) studied treatment (*n* = 1358) and control (*n* = 1346, total *n* = 2704) at 30 months after the intervention began. The percentage of the participants who were male was not reported. In this study, no statistical pre‐test differences were found on outcomes or demographics. The mean age of participants was not reported, although the infant participants would have been around the age of 2–3 years old. Using interview and telephone survey data, they analysed data on participant‐owned and state‐owned account opening and saving amount. We could not calculate the effect size of the intervention on participant‐owned account opening because they were combined with state‐owned account opening, which was part of the intervention. We could not calculate the effect size for savings amount, as the study authors did not report sufficient information. Study authors reported that the mean savings for the treatment group was $1130 (*n* = 1358) and the mean savings for the control group was $75.7 (*n* = 1346).

Beverly, Kim, et al. (2015) studied *n* = 2698 (*n* = 1353 treatment group, and *n* = 1345 control group) at 3 years post‐baseline for participant‐owned and state‐owned accounts. In this study, no statistical pre‐test comparison was made on outcomes, and no statistically significant differences on pre‐test demographics were found. The mean age and gender of participants were not reported, although the infant participants would have been between 2 and 3 years old at the time of the study. Using telephone survey and interview data, they analysed account opening and savings amounts for all accounts. We could not calculate the effect size of the intervention on participant‐owned account opening because they were combined with state‐owned account opening, which was part of the intervention. We could not calculate the effect size for savings amount, as the study authors did not report sufficient information. The study authors reported that the mean saving amount for the treatment group was $1129.85 (*n* = 1353) and the mean saving amount for the control group was $75.74 (*n* = 1345).

Huang et al. (2015) examined effects at 6 years post‐baseline for participant‐owned and state‐owned accounts. Their sample included *n* = 2704 (*n* = 1358 treatment group, and *n* = 1346 control group) participants. In this study, no statistical pre‐test comparison was made on outcomes, and no statistically significant pre‐test differences on demographics were found. The mean age of the parent participants was 25.5 years old, and the mean age of the infants was not reported, although the infants would have been around the age of six. About half (52.48%) of the children were male. Using baseline telephone survey and administrative data, they analysed account opening for participant‐owned accounts, savings amount for participant‐owned accounts, and asset value. The effects of the intervention on account opening were large (*d* = 1.63, 95% CI = 1.33 to 1.93). We could not calculate the effect size for savings amount or asset value, as the study authors did not report sufficient information. The study authors reported that the mean saving amount for the treatment group was $153.71 (*n* = 1358) and the mean saving amount for the control group was $31.96 (*n* = 1346). The study authors reported that the mean asset value for the treatment group was $1605.07 (*n* = 1358), while the mean asset value for the control group was $50 (*n* = 1346).

Wikoff et al. (2015) examined effects at 5 years post‐baseline for participant‐owned accounts. Their sample included *n* = 2626 (*n* = 1318 treatment group, and *n* = 1308 control group) participants at 4–5 years after baseline telephone interview survey. In this study, no statistical pre‐test comparison was made on outcomes and statistically significant pre‐test differences on demographics were found. The mean age of the parent participants was 25.57 years old, and the mean age of the infants was 39 months. About half (53.1%) of the children were male. Using administrative data and baseline telephone survey data, they analysed the extent to which participants opened participant‐owned accounts. The effect of the intervention on participant account opening was large (*d* = 1.71, 95% CI = 1.38 to 2.03).

Clancy et al. (2016) examined effects at 7 years post‐baseline for participant‐owned and state‐owned accounts. Their sample included *n* = 2703 (*n* = 1357 treatment group, and *n* = 1346 control group) participants. In this study, no statistical pre‐test comparison was made on outcomes, and statistically significant differences on pre‐test demographics were found. The mean age of the parent participants was 25.6 years old, and the mean age of the infants was not reported, although the infants would have been around the age of seven. The gender of participants was not reported either. Using telephone interview, survey, and administrative data, they analysed account opening and savings amount for state‐owned and participant‐owned accounts. We could not calculate the effect size of the intervention on participant‐owned account opening because they were combined with state‐owned account opening, which was part of the intervention. We could not calculate the effect size for savings amount, as the study authors did not report sufficient information. The study authors reported that the mean saving amount for the treatment group was $1,851 (*n* = 1358) and the mean saving amount for the control group was $322.8 (*n* = 1346).

Huang et al. (2017) examined effects at 5 years post‐baseline for participant‐owned accounts. Their sample included *n* = 2677 (*n* = 1343 treatment group, and *n* = 1334 control group) participants. In this study, no statistical pre‐test comparisons were made on outcomes or found on demographics. In this study, the treatment and control group were matched based on demographics. The mean age of the parent participants was 20–29 years old, and the mean age of the infants was not reported, although the infants would have been around the age of five at the time of this study. The percentage of the children that were male was not reported. Using administrative data and baseline telephone survey, they analysed savings amounts and the extent to which participants opened participant. The effects of the intervention on participant account opening were large (*d* = 1.62, 95% CI = 1.31 to 1.92). We could not calculate the effect size for savings amount, as the study authors did not report sufficient information. The study authors reported that the mean saving amount for the treatment group was $152.93 (*n* = 1343) and the mean saving amount for the control group was $32.18 (*n* = 1334).

Huang et al. (2019) studied the effects of the SEED OK intervention on a subsample of low‐income families (total *n* = 426, treatment *n* = 201 and control *n* = 225) enroled in either of two public welfare programs: Temporary Assistance for Needy Families (TANF) and/or Head Start. They studied participant‐owned accounts in the study. Study authors used a follow‐up survey and administrative records to study outcomes at 4 years post‐intervention, which was 7 years after baseline. In this study, no statistical pre‐test comparison was made on outcomes, and no statistically significant differences on pre‐test demographics were found. The mean age of the parent participants was 24 years old, and the mean age of the infants was not reported, although the infants would have been around the age of seven. The predominant race was White. 55% of the children were male, and all of the parents were female. Neither the treatment nor the control group had high attrition. Study authors reported opening account and asset value outcomes at 4 years post‐intervention, which was also 7 years after baseline. For participant‐owned accounts, a small significant effect size was found (*d* = 1.524, 95% CI = 0.149 to 2.898). A small significant effect size was also found for asset value (*d* = 3.153, 95% CI = 2.868 to 3.437).

##### Osborne et al. (2016)

Using a quasi‐experimental design, Osborne et al. (2016) studied the effects of a school‐based Children's Development Account program with 9–10 years old school children in central Texas (US). For the treatment group, fourth‐grade students in five elementary schools received high‐intensity financial education (face‐to‐face, classroom) paired with the opportunity to open a Children's Development Account. For the comparison group, students in two other schools were offered financial education but not offered the opportunity to open an account until the treatment was over. Treatment was 10 weeks. The majority of the treatment group were from ‘economically disadvantaged families’, but the income level of treatment or comparison group participants was not specified. Students in both groups took a pre‐test before beginning financial education and a post‐test 1 week after financial education concluded. Deposits up to $50 were matched one‐to‐one for the treatment group. Researchers collected pre‐test and post‐test data on financial education, administrative data from the financial institution and school, and conducted focus groups and interviews. The method of assignment of schools (and participants within the schools) to condition was not reported. In this study, statistical pre‐test comparisons were not made on differences in outcomes or demographics. The treatment and control groups were not matched. The mean age of the participants was 9–10 years old. The percent of the sample that was male was not reported. The predominant race of the participants was Hispanic. School officials and bank representatives delivered the intervention. The role of the evaluator was not reported. The frequency of contact was three times per week for 10 weeks (for the financial education). The attrition for both the treatment and control group was not high (under 20%). The study authors did not report whether the intervention was manualized, or whether fidelity was assessed.

Overall, the risk of bias is high for this study for sequence generation. The risk of bias is unclear for allocation concealment, blinding of participants and personnel, selective outcome reporting, and other. The risk of bias is low for blinding of outcome assessors and incomplete outcome data. Using administrative data, study authors analysed account opening and savings amounts. The total sample was *n* = 393 (treatment group *n* = 196, control group *n* = 197). Study authors did not report account opening or saving amounts for the control group, thus, effect sizes could not be calculated. Study authors report that 13.8% of the treatment group opened accounts, and the mean savings for the treatment group was $135.06.

#### 5.3.4 Youth Bank Accounts (Loke et al., 2016)

Loke et al. (2016) used a quasi‐experimental design to test a youth financial capability initiative within a youth workforce development program in San Francisco, California (US). The 12–30‐week intervention targeted low‐income working youth for financial education and non‐custodial bank accounts. The intervention was tested on *n* = 375 (total sample size) low‐income 16–21‐year‐old youth who participated in a youth workforce and employment programs operated by 10 non‐profits in partnership with San Francisco's Department of Children, Youth and their Families. The study individually assigned to one of two treatment groups or a comparison group, although the size of the treatment and comparison groups is not reported. Both treatment groups received high‐intensity financial education (multi‐session, face‐to‐face) and supported enrolment into bank accounts, among other related features. One treatment group also received two hours of peer‐led group coaching. The comparison group only received financial education about alternative (non‐bank) financial services.

In this study, statistical pre‐test comparisons were not made on differences in outcomes, but statistically significant pre‐test differences on demographics were found. The treatment and control groups were not matched. The mean age of the participants was 16–21 years old. 44.5% of the sample was male. The predominant race of the participants was Asian, and 66.5% of the subjects had received financial education previous to the intervention. Non‐profit staff delivered the intervention. The role of the evaluator was separate from the delivery of the intervention. The frequency of contact was periodic. The attrition rate for both the treatment and control group, the number of sessions, whether the treatment was manualized and whether fidelity was assessed were not reported.

Using pre‐test and post‐test survey data and administrative data, the study authors evaluated saving amount at the end of treatment, but reported for the treatment groups only, thus we cannot calculate an effect size. Study authors reported that 97% of the youth in the treatment groups established a savings account and 96% met their savings goal. All of the participants in the treatment group saved some portion of their income, with total savings ranging from $9 to $2,268. Overall, the risk of bias is high for this study for sequence generation and other (research allegiance confounds). The risk of bias is unclear for allocation concealment, blinding of participants and personnel, incomplete outcome data, and selective outcome reporting. The risk of bias is low for blinding of outcome assessors. The risk of bias is also high because only outcomes for treatment groups are reported.

#### 5.3.5 Retirement Accounts

Duflo et al. (2006) analysed a randomized control trial of a retirement account intervention with tax filers in St Louis, Missouri (US) who used the private company H&R Block as paid tax preparers during 1 month of tax‐season. Participants were all tax filers of any income using the service during the 1‐month intervention period, and were individually randomly assigned to the treatment or control group. While participants were physically at the tax preparation office, tax preparers delivered low‐intensity financial education (brief, face‐to‐face, only related to their taxes and retirement savings), along with matches of 20% (treatment group 1) and 50% (treatment group 2) of participant contributions into the self‐retirement funds (IRAs) from the amount of money refunded to them through their tax returns. The control group received no financial education and zero match. The total sample was *n* = 13,952 [treatment group 1 *n* = 4521 (20% match), treatment group 2 *n* = 4722 (50% match), and control group *n* = 4,719 (zero match and no financial education)].

In this study, statistical pre‐test comparisons were not made on differences in outcomes or demographics. The treatment and control groups were not matched. The mean age of the participants was not reported. The predominant race of the participants or gender of participants was not reported. The role of the evaluator was separate from the treatment. The frequency of contact was once, for just one session. The attrition for both the treatment and control group was less than 20%. The study authors did not report whether the treatment was manualized or fidelity assessed. The review authors assessed the risk of bias to be low for bias domains of sequence generation, incomplete outcome data, and blinding of outcome assessors. The risk of bias was assessed to be unclear for allocation concealment, blinding of participants and personnel, and selective outcome reporting. The risk of bias was assessed to be high for other (research allegiance). Using administrative data, study authors analysed savings amounts and account opening at the end of treatment. Study authors did not provide sufficient information to calculate an effect size for savings amount. The effects of the intervention on account opening for the treatment group 1 that received a 20% match compared to the control group was large (odds ratio [OR] = 2.79; CI = 2.28 to 3.41). For treatment group 2 that received a 50% match compared to the control group, the effect of the intervention was also large (OR = 4.67; CI = 3.87 to 5.64).

Lusardi et al. (2009) used a quasi‐experimental design to study a retirement‐focused intervention geared towards female and low‐income employees of Dartmouth College (US), because these populations traditionally save little for retirement. They had a total sample of *n* = 124 (treatment group *n* = 64, comparison group *n* = 60). The study authors measured outcomes at the end of intervention period of 6 months. The treatment was delivered during new employee orientation, the length of which is not reported. The study authors scheduled the intervention during the first 6 months of the year (Jan‐June) within which females and low‐income employees are more likely to attend new employee orientation, as compared to higher‐income faculty members (July–‐December). During the one‐time new employee orientation, the treatment group received low‐intensity financial education (flyer inserted in the employee benefits packet, a visual presentation, and an instructional video) and supported access to retirement accounts. The comparison group received the usual employee orientation that did not include financial education. The comparison group consisted of employees who had completed new employee orientation a year before the intervention during the same season (Jan‐June), when they had not received the low‐intensity financial education, but could have signed up for the retirement plan.

Statistical pre‐test comparisons were not made on differences in outcomes or demographics. The treatment and comparison groups were not matched. The mean age of the parent participants was not reported. The predominant race of the participants and gender of participants was not reported. University staff delivered the intervention. The role of the evaluator was not independent of the treatment. The attrition rate for both the treatment and control group was not reported. Study authors did not report whether the treatment was manualized or fidelity was assessed.

Using posttest survey data, study authors studied the outcomes of account opening.

The study authors did not include data needed to calculate an effect size for account opening. Study authors reported that the intervention resulted in a 56.2% increase in account opening within 30 days of the intervention compared to the comparison group. The review authors noted that the risk of bias is high for this study for sequence generation, and other (research allegiance), and unclear for allocation concealment. The risk of bias is also unclear for blinding of participants and personnel, incomplete outcome data, and selective outcome reporting. The risk of bias is low for blinding of outcome assessors.

Goda et al. (2012) used a randomized control trial with employees of the University of Minnesota (US) to test an intervention using low‐intensity financial education (direct‐mail) and supported signup for retirement accounts. The study authors randomly assigned employees by departments (*n* = 16,881) to four groups, a control group (*n* = 4063) and three treatment groups (n = 12,828; group 1 *n* = 4434, group 2 *n* = 4353, and group 3 *n* = 4131). The control group received nothing, and the three treatment groups received varying levels of detailed financial education about retirement. The intervention lasted for 7 months, and the intervention was evaluated at the end of treatment.

Subjects were individually randomly assigned to condition based on their department and matched‐quads, which were formed based on retirement participation rate, age and salary. Statistical pre‐test comparisons were not made on differences in outcomes or demographics. The treatment and comparison groups were matched based on demographics. The mean age of the participants was 44.89 years old. The predominant race of the participants was not reported, and 44.3% of the sample was male. All income levels were included. University staff delivered the intervention. The role of the evaluator was independent of the treatment. The frequency of contact, number of sessions, whether the treatment was manualized or fidelity assessed, and there was no attrition for the treatment and control group. The review authors assessed the risk of bias to be low for the bias domains of sequence generation, allocation concealment and blinding of outcome assessors. The risk of bias was assessed to be unclear for blinding of participants and personnel, incomplete outcome data, selective outcome reporting and other. Study authors used administrative data to study savings amount and retirement savings rate. The effect of the intervention on the saving amount, compared to the control group, was trivial and not statistically significant for all treatment groups (treatment group 1, —*d* = −0.014, 95% CI = −0.06 to 0.03; treatment group 2, *d* = −0.02, 95% CI = −0.02 to 0.07; and treatment group 3, *d* = −0.001, 95%, CI = −0.04 to 0.04). The effect of the intervention on the retirement saving rate was also trivial and not statistically significant for each of the treatment groups compared to the control group (treatment group 1, *d* = −0.02, 95% CI = −0.06 to 0.02; treatment group 2, *d* = 0.0236, 95% CI = −0.02 to 0.07; and treatment group 3, *d* = 0.03, 95%, CI = −0.01 to 0.07).

Collins and Urban (2016) (retirement savings) used a quasi‐experimental design to study an intervention that paired financial education with supported signup or changes to an employer‐provided retirement plan for the employee participants (n = 1001) of 45 Wisconsin (US) credit unions. Treatment participants completed a 10‐module online financial education program (high‐intensity) and could sign‐up for a retirement plan or, if previously enroled, continue or alter their retirement savings behaviour. The length of the intervention time period is about 4 months. Participants were assigned to condition based on their credit union. Comparison group members received nothing.

Using pre‐test and posttest survey data and administrative data starting 4 months post‐baseline for 11 months after treatment ended, study authors studied the financial behaviour outcomes of account opening, retirement plan rate, budgeting, as well as the financial outcome of savings (emergency) amount. The total sample was *n* = 1001 (treatment group *n* = 301, comparison group *n* = 700).

Statistical pre‐test comparisons were not made on differences in outcomes, but statistically significant differences were found on demographics. The treatment and comparison groups were not matched. The mean age of the parent participants was not reported. The predominant race of the participants was white. 19% of the participants were male. All income levels were included in the groups. The intervention setting was the credit union, and the staff delivered the intervention. The role of the evaluator was separate from the treatment. Neither the frequency of contact nor the length of sessions was reported. The attrition for both the treatment and control group was not reported and could not be calculated. It was not reported whether the treatment was manualized or fidelity was assessed. We were unable to report effect sizes with the data provided. The study authors reported that the intervention increased account opening by 6%, and the rate of creation of a budget by almost 6% more than the comparison group. The intervention increased the retirement savings participation by 3.7%–3.8%, the retirement savings rate by 40.4%, and emergency savings by 3.8% as compared to the comparison group. The risk of bias is high for this study for sequence generation and other (research allegiance), and unclear for allocation concealment, blinding of participants and personnel, incomplete outcome data, and selective outcome reporting. The risk of bias is low for blinding of outcome assessors.

#### 5.3.6 Pre‐Purchase Home Buying Education (Smith et al., 2017)

Smith et al. (2017) used a randomized control trial in Philadelphia, Pennsylvania (US) to test the effects of pre‐purchase homeownership financial education and supported access to a home mortgage loan. Study subjects, who were renters attempting to become first‐time homebuyers, were individually randomly assigned to a treatment group (*n* = 202) or the control group (*n* = 202), for a total sample size of *n* = 404. The treatment group received high‐intensity financial education and one‐on‐one counselling (face‐to‐face, individualized) that included individualized supported access to home mortgage products. The control group received the routine pre‐purchase workshop only.

In this study, the length of treatment was not reported. Non‐profit staff members delivered the intervention at a community based non‐profit organization. Staff had periodic contact with treatment subjects, but the number of sessions was not reported. The intervention was evaluated 5 years after baseline measurement. Statistical pre‐test comparisons were not made on differences in outcomes, and no statistically significant differences were found on demographics. The treatment and comparison groups were matched using propensity score matching. The mean age of the parent participants was 25–34 years old. The predominant race of the participants was African American. 32.3% of the sample was male. All income levels were included. The role of the evaluator was independent of the treatment. The intervention was manualized, and fidelity was assessed. However, the method by which fidelity was assessed was not reported. Both the treatment and control group had high attrition (more than 20%).

Using baseline and annual surveys, paired with administrative data, the study authors studied credit scores, saving amount and debt 5 years after the baseline survey. The study authors did not provide sufficient information to calculate effect sizes. The saving and debt amounts were only reported by homeownership status. Treatment group participants who were homeowners saw a statistically significant decrease in debt compared to the control group homeowners. There were no statistically significant differences on debt or saving amount between non‐home owning treatment and control participants. Regarding credit scores, the study authors reported no significant difference between the treatment and control groups. The review authors assessed the risk of bias to be low for the bias domains of sequence generation, blinding of outcome assessors, and allocation concealment. The risk of bias was assessed to be unclear for blinding of participants and personnel, incomplete outcome data, selective outcome reporting and other.

#### 5.3.7 Adult financial education, counseling and/or coaching paired with financial access

##### Parks Opportunity Program (also called the ‘Assessing Financial Capability Outcomes Adult Pilot’)

The New York Parks Opportunity Program used a randomized control trial design to test the financial capability outcomes of pairing a low‐cost transaction account with financial counselling and education. Participants in the study were low‐income adult employees of the New York City Parks Opportunity Program within the Department of Parks and Recreation. The program was a 6‐month workforce development program for adults moving off public assistance. The program offers 6 months of full‐time employment coupled with job search counselling and other services. Staff of the New York City Department of Consumer Affairs Office of Financial Empowerment managed the implementation of the pilot program and research and delivered the financial education. A local bank partnered with the effort to offer bank accounts.

Participants were randomly assigned to treatment or control group, based on month of their hire and work site. Randomly selected employees (treatment group) were offered one‐on‐one, face‐to‐face, individualized (high‐intensity) financial education and counselling, regardless of whether they had previously signed up for an account or not. Both the treatment and control groups were offered the option to open a bank account. The intervention occurred for about 1 year, including a counselling session and two follow‐up surveys. The treatment (financial education and account sign‐up) lasted from 30 min to 1 h. Participants had the option to return for a second session. The sample was low‐income. The setting of the intervention was not reported. The role of the evaluator was separate from the intervention. The intervention included about three contacts with the treatment group members. Whether fidelity was measured or the intervention was manualized was not reported, and attrition of the treatment and control groups was not reported. The treatment and control groups were not matched. Sub‐study authors used survey and administrative data, as well as credit reports and scores and bank transaction data from financial institutions, employers and non‐profits.

For the three sub‐studies, risk of bias was assessed as low for the bias domain of blinding of outcome assessors. The risk of bias was assessed to be unclear for the domains of blinding of participants and personnel and other. Because the studies reported different outcomes, the studies are at high risk of selective outcome reporting. Due to the fact that the studies used different sample sizes, the studies are at high risk for incomplete outcome data. The risk of bias from allocation concealment is low.

Gons (2013) used a total sample size of *n* = 1034 (no treatment group or control group n was reported) and assessed outcomes at 9 months post‐intervention. 21% of the sample was male. The predominant race of the participants was African American. About 40% of all of the participants who applied for a checking account were unable to open one, often due to their name appearing on a consumer debt registry. In this study, statistical pre‐test comparisons were not made on differences in outcomes or demographics. Gons and colleagues evaluated the outcomes of debt and credit scores at 9 months post‐treatment. The study author did not report sufficient information to calculate effect sizes for any of the outcomes of interest for this review. The study author reports that study participants had ‘better outcomes with respect to credit scores, lower levels of revolving debt and fewer accounts in collections than those who did not receive financial counselling’. The risk of bias from sequence generation is low.

Wiedrich et al. (2014) reported results of participants that took part in the study from four sites in New York. They reported 1034 participants (treatment group = 505, control group *n* = 529) took part in the study, but about half did not have credit scores, so thus a portion of their total participants are included in the credit score analysis. The predominant race was African American, and 22% of the sample was male. Using survey and administrative data collected at baseline, 6 and 12 months after enrolment, study authors analysed the outcome of credit score. Study authors note 55% overall sample attrition, although not broken down by control and treatment group. Study authors reported credit scores at (*d* = 0.24, 95% CI = 0.06 to 0.42) and 12 months (*d* = 0.15, 95% CI = −0.4 to 0.33) post‐intervention. As noted by the study authors, the positive results in the first 6 months were reversed as the control group caught up to the treatment group by 12 months.

Collins and Nafzinger (2019) included eight sites (four sites reported in Wiedrich et al. (2014) and an additional four sites), with a total sample size of *n* = 865 (treatment group *n* = 509, control group *n* = 356). The authors assessed outcomes at 6 and 12 months post‐intervention. The predominant race was African American, and 22% of the sample was male. About 23% of all of the participants who applied for a checking account were unable to open one, often due to their name appearing on a consumer debt registry. Using survey and administrative data collected at baseline, 6 and 12 months after enrolment, study authors analysed the outcome of credit score. While the control group did not experience high attrition, study authors reported high attrition for the treatment group. Study authors reported credit scores at 6 and 12 months post‐intervention. Study authors report that in the first 6 months post‐intervention, the treatment groups experience ‘some increase’ in credit scores. However, during the 6–12‐month period, the control group also experienced increased in credit scores and caught up to the treatment group. At the 12‐month mark, there was no measurable effect of counselling on credit scores. The study authors also reported Study authors report that the intervention led to 13% reduction in past debt due over a 12‐month period for the treatment group. For the outcome of savings amount, the treatment group experienced reduced bank balances in the 6–12‐month post‐intervention period, which was not statistically significant. Post‐intervention, the mean bank balance for the treatment group was $41 less.

###### 5.3.7.1 Kim et al. (2005)

Kim et al. (2005) used a quasi‐experimental design to examine the effects of face‐to‐face financial education and counselling focused on credit and debt (high‐intensity financial education) on financial behaviours and financial outcomes of people who had debt challenges. 1800 participants were randomly selected from the population of 4000 new clients who committed to a debt management plan with a non‐profit credit counselling organization. Participants were clients of a large non‐profit credit counselling agency that provides telephone counselling services. Participants in the treatment group had debt management plans approved by their creditors and remained in the program for 18 months, which is the length of the treatment. The comparison group contacted the credit counselling agency, then did not complete the 18 months intervention period. Both groups received one financial education session with staff, and the treatment group received additional phone financial education during the 18 months, as well as access to online financial educational materials (high intensity). The total sample was *n* = 166, (treatment group was *n* = 68 and comparison group was *n* = 98). The measurement of outcomes was 18 months post‐baseline.

Staff in the non‐profit setting delivered the intervention. Statistical pre‐test comparisons found differences on outcomes and demographics. The treatment and comparison groups were not matched. The mean age of the participants was not reported. The predominant race of the participants was White, and 32% of the sample was male. Participant income level was not reported. The role of the evaluator was not reported. The frequency of contact, number of sessions, length of the treatment and whether the intervention was manualized was not reported. Fidelity was not assessed. Both the treatment and control group had a high attrition rate (more than 20%).

Using pre‐and post‐intervention survey data, study authors studied the outcomes of budgeting and debt. The effects of the intervention were large on budgeting (OR = 3.14; CI = 1.55 to 6.39) and lowering debt (OR = 6.4; CI = 2.13 to 19.25). The review authors noted that the risk of bias is high for this study for sequence generation, incomplete outcome data and blinding of participants and personnel. The risk of bias is unclear for blinding of outcome assessors, allocation concealment, selective outcome reporting, and other.

###### 5.3.7.2 Moulton et al. (2015)

Moulton et al. (2015) used a randomized control experimental design to test the impact of financial education and financial coaching on mortgage delinquency for first‐time low‐income homeowners. In partnership with the Ohio Housing Financial Agency (OHFA), a state agency that issues bonds to fund mortgages for qualified borrowers, researchers created and studied the ‘MyMoneyPath’ program implementation, which was designed to reduce mortgage delinquency and default. The treatment group (*n* = 295) received an online individualized financial assessment, low‐touch (online) financial education that involved budgeting and goal‐setting before their mortgage loan closure, and individualized post‐purchase financial coaching delivered quarterly by a non‐profit organization. Participants were individually randomly assigned to groups. No pre‐test statistically significant differences were found on outcomes or demographics. The groups were not matched. Control group participants (*n* = 130; total *n* = 425) received the online financial education only. Researchers and OHFA staff designed the intervention, and researchers trained the non‐profit staff who delivered the financial coaching. The intervention lasted 12 months, and was manualized. The number of sessions or whether fidelity was measured was not reported. Only the treatment group had high attrition (more than 20%).

Using administrative records, a follow‐up survey and credit report records, study authors found that the mean age of the participants was 33 years old. The participant predominant race was White, and 54% of their sample were male. The study authors measured saving amount and credit scores at 12 months post‐intervention. Effects sizes were small and not significant for savings amount (*d* = 0.127, 95% CI = −0.081 to 0.334) and credit score (*d* = −0.038, 95% CI = −0.245 to 0.17). The review authors found that the risk of bias is high for incomplete outcome data. The risk of bias is low for sequence generation, and blinding of outcome assessors. The risk of bias is unknown for other (research allegiance, funding), blinding of participants and personnel, allocation concealment, and selective outcome reporting.

###### 5.3.7.3 Theodos et al. (2016)

Theodos et al. (2016) used a randomized control trial to test the effects of financial education and consumer credit card access. Subjects were members of the Arizona Federal Credit Union in Phoenix, Arizona (US) who were considered ‘credit card revolvers’, described as people who carry debt on their credit card from month to month for at least 2 of the 6 pre‐intervention months, not necessarily consecutively. Study authors used a sample of individuals from their membership who were credit revolvers and randomly assigned them to be either in the treatment or control group. The total sample was *n* = 13,957 (treatment group *n* = 12,213, control group *n* = 1744). The control group received nothing, but had continued access to their credit card. The treatment group received low‐intensity financial education (i.e., email, online, and a direct mailer) related to use of credit and continued access to their credit card.

In this study, the length of treatment was 6 months. Non‐profit staff delivered the intervention. Staff had periodic contact with treatment subjects. The intervention was evaluated 6 months before and after treatment. Statistical pre‐test comparisons found differences on outcomes, but not demographics. The treatment and comparison groups were not matched. The mean age of the parent participants was 46 years old. The predominant race of the participants was not reported, and 55% of the sample was male. All income levels were included. The role of the evaluator was independent of the treatment. The number of sessions was not reported. Whether the intervention was manualized was not reported, and fidelity was not assessed. Neither the treatment and control group had a high attrition rate (less than 20%).

Using administrative data, study authors studied credit score and debt before and during the 6‐month treatment period. The study authors did not provide sufficient data to calculate an effect size for either credit score or debt. For credit score outcomes, study authors report that the mean of the treatment group post‐intervention was 702.42 and 703.7 for the control group. For the debt outcome, study authors report that the mean debt for the treatment group was $4926, and $5118 for the control group. The review authors assessed the risk of bias to be low for the bias domains of sequence generation, blinding of outcome assessors, incomplete outcome data, and allocation concealment. The risk of bias was assessed as unclear for blinding of participants and personnel, selective outcome reporting and other.

###### 5.3.7.4 Roder (2016)

Roder (2016) used a quasi‐experimental design to examine a program that combines individualized financial counselling focused on credit, financial education and access to financial products/services for saving and building credit. Participants were unemployed adults primarily seeking assistance from five community‐based non‐profit agencies in Chicago, Illinois (US) for employment and financial and income services, including assistance in obtaining public benefits. As part of their service delivery model, individualized one‐on‐one (high‐intensity) financial education and coaching, and financial access was also offered to participants. The treatment group (*n* = 500) were those participants who received financial education and supported access to financial products/services from the non‐profit agencies. The comparison group (*n* = 649) were subjects who received employment and training only from public (city) workforce centres (total sample *n* = 1149). The length of treatment was 6 months to 3 years.

Non‐profit staff in the non‐profit setting delivered the intervention. The treatment and comparison groups were matched using propensity score matching based on employment experience, demographics and financial situation. After matching, statistical pre‐test comparisons did not find differences on outcomes or demographics. The mean age of the participants was 38 years old. The predominant race of the participants was African American and 45% of the sample was male. Participants were low‐ and moderate‐income. The role of the evaluator was independent of the intervention. The frequency of contact was periodic, with a mean of nine sessions. The study authors did not report whether the intervention was manualized, and fidelity was not assessed. Both the treatment and control group had high attrition (more than 20%).

Outcomes were measured at 2 years post treatment. Using phone surveys, credit reports, administrative data, program observations and staff interviews, study author studied the outcome of credit scores. The effect of the intervention on credit scores is trivial. The study authors report a Hedges *g* effect size of 0.01. The study author did not report the confidence intervals or sufficient information for the review authors to calculate an effect size. The review authors noted that the risk of bias is high for this study for sequence generation and incomplete outcome data. The risk of bias is low for blinding of outcome assessors. The risk of bias is unclear for allocation concealment, blinding of participants and personnel, selective outcome reporting, and other.

###### 5.3.7.5 Modestino et al. (2019)

Modestino et al. (2019) used a randomized control experimental design to study an intervention for low‐income young adult (age 18–19) participants in a workforce development program that combined financial education, individualized financial coaching, and a credit building product. Working with a national non‐profit, the Boston Youth Credit Building Initiative was developed by the Boston Mayor's Office of Financial Empowerment. The treatment group (*n* = 150) intervention consisted of a one‐hour financial education workshop, a 1‐h individualized financial coaching session with individualized follow‐up services, and a credit‐building loan product, whereby participants' loan payments are reported to the credit bureaus. Participants were individually randomly assigned to groups using stratification by age, race and gender. No pre‐test statistically significant difference was found on outcomes, but statistical controls were used to control for pre‐test statistically significant differences on demographics (i.e., education and race). The groups were not matched. Control group participants (*n* = 150; total *n* = 300) received no services. Both the treatment and control group received financial incentives to participate in the study for 1 year. Non‐profit staff delivered the intervention on the work site, and the evaluators were independent of the intervention. The intervention lasted 18 months. The frequency of the contact, number of sessions, whether the intervention was manualized, or whether fidelity was measured was not reported. Both the treatment and control group had high attrition (more than 20%).

Using credit report and pre‐ and post‐intervention survey data, study authors found that the mean age of the participants was 23 years old. The participant predominant race was African American, and the gender distribution was not reported. The study authors measured credit scores at 18 months post‐intervention. Authors did not provide sufficient data to calculate effect sizes. Study authors reported significant increase in credit scores among those who initially had a credit file at baseline compared to the control group. The review authors found that the risk of bias is high for incomplete outcome data. The risk of bias is low for sequence generation and blinding of outcome assessors. The risk of bias is unknown for other (research allegiance), allocation concealment, selective outcome reporting, and blinding of participants and personnel.

#### 5.3.8 Tax refund saving and investment

##### Refund to savings

Four of the five studies described below emanate from the same project, Refund to Savings (R25) initiative. There are many similarities among the studies. However, they are not described here as sub‐studies from a large longitudinal study because they each have a unique sample of the same experiment. Thus, *each of the four R2S studies is a separate study* for the analysis. However, the similarities are noted below among the four.

The R2S initiative is a collaboration among the Center for Social Policy at Washington University in St Louis, Duke University, and Intuit, Inc., the maker of TurboTax software. Together they designed and tested an intervention that encourages low‐ and moderate‐income tax filers using specific tax filing software to save a portion of their federal tax refund and streamline the process of depositing refunds directly into saving vehicles while filing their taxes. Messages are built directly into the software edition designed to serve low‐ and moderate‐income households for free, in partnership with the Internal Revenue Services' Free File Alliance. Each of the R2S studies uses a randomized controlled design. Each of the studies discussed below has gathered data from a different year. Each has tested different messages and message combinations related to finances (low‐touch financial education) to encourage filers to deposit a portion of their tax return into savings, although this review does not incorporate the differential effects of the various messages or combinations. Filers were individually randomly assigned to treatment and control groups through the software program after learning that they would receive a federal tax return. The program automatically and randomly created several treatment group arms by providing a variety of messages to the (larger) treatment group. After reading the messages, filers could navigate the choice options in seconds or minutes about whether to save, how much to save of their federal refund, and where those savings would be saved (i.e., direct deposit into an account, savings bond purchase, etc.). Administrative data from the software is used for each of the four studies. With the exception of the 2012 data (reported in Grinstein‐Weiss, Russell, et al., 2017), follow‐up survey data, one immediately after filing the tax return, and a second 6 months later, were a secondary data source. The groups were not matched. Whether the intervention was manualized, or whether fidelity was measured was not reported. Neither the treatment nor control groups had high attrition. The review authors noted that the risk of bias is low for this study for sequence generation, blinding of outcome assessors, allocation concealment, incomplete outcome data, and blinding of participants and personnel. The risk of bias is unclear for selective outcome reporting and other (research allegiance, funding, etc.).

Grinstein‐Weiss et al. (2015) used R2S 2013 data. Data were collected for approximately 2.5 months between February and mid‐April, 2013. The size of the treatment and control groups is not reported, but the total *n* = 680,545. No pre‐test statistically significant differences were measured on outcomes or found on demographics. The mean age of the sample was 34, and the predominant race was White. 40% of the sample were male. Study authors reported saving amount outcome. Authors did not provide sufficient data to calculate effect sizes. Study authors reported that, in periods 1 and 2, the proportion of tax filers in every treatment condition depositing any tax refund into a savings vehicle was statistically higher than the control group.

Grinstein‐Weiss, Cryer, et al. (2017) used R2S 2015 data, which were collected from mid‐January through early June, 2015 (approximately 5 months). The treatment group was *n* = 484,164, and the control group was *n* = 161,952 (total *n* = 646,116). No pre‐test statistically significant differences were measured on outcomes or found on demographics. The mean age of the sample was 35.25. The predominant race and gender balance of the sample were not reported. Study authors reported saving amount and saving rate outcome data, generated upon completion of filing tax return. For saving amount, study authors reported that treatment participants in three conditions saved more than the control group: (a) the Choice Architecture condition (*d* = 0.22, *p* = 0.009); (b) the Savings Emphasized Twice condition (*d* = 0.29, *p* < 0.001); and (c) the Savings‐Single Click condition (*d* = 0.36, *p* < 0.001). For saving rate outcome data, study authors report that 13% of treatment group saved compared to 8% of control participants.

Grinstein‐Weiss, Russell, et al. (2017) used 1 month of data collected between March, 2012 and April 2012. The treatment group was *n* = 95,669, and the control group was *n* = 11,963 (total *n* = 107,632). No pre‐test statistically significant differences were measured on outcomes, but differences were found on demographics (i.e., marital status) that were controlled for in the study. The mean age of the sample, predominant race, nor the gender balance were reported. Study authors reported saving amount outcome. Authors did not provide sufficient data to calculate effect sizes. Authors reported that the treatment groups saved significantly more than the control group.

Roll et al. (2018) report on 2016 data, which were collected from mid‐January through early June, 2016 (approximately 5 months). The treatment group was *n* = 70,978, and the control group was *n* = 213,147, for a total *n* = 284,125. No pre‐test statistically significant differences were measured on outcomes or demographics. The mean age of the sample was 35.3 and the predominant race was White. The gender balance was not reported. Study authors reported saving amount outcome. Authors did not provide sufficient data to calculate effect sizes. Study authors reported finding statistically significant differences between treatment and control groups on rate of depositing any refund and savings dollar amounts.

##### Other tax refund study (Tufano, 2011)

Tufano (2011) reports on a 2007 experiment that used a quasi‐experimental design with parallel cohorts to test whether a financial education at the point of tax filing can impact filer saving and investment for low‐ and moderate‐income filers. One large commercial tax filing company, a non‐profit and an academic research team collaboratively created and implemented the intervention. Thirty‐one tax preparation sites of one commercial company in Massachusetts and in Illinois were used for the intervention. Participants were assigned to the treatment and control groups based on the site where they received their tax filing services. 16 offices in Boston and 11 offices in Illinois were treatment sites, and four offices in Boston were control group sites. Commercial tax preparation staff provided the intervention, and the evaluator was independent of delivering the intervention. There was only one session in the intervention, which was manualized and fidelity was measured through administrative records.

The treatment group (*n* = 3730) were tax filer customers who learned that they were receiving a tax refund, and were educated about (i.e., low‐touch financial education) and provided an opportunity to purchase a US Saving Bond with their tax refund funds. The control group (*n* = 1484; total *n* = 5214) received the typical tax filing services without an offer to purchase a savings bond with their refund funds. The groups were not matched. Pre‐test statistically significant differences were found on outcomes (i.e., plan to save their refund) and on demographics (e.g., gender and homeownership). Statistical controls were used to control for these differences. Neither the treatment nor the control group had high attrition.

Using survey data for both the treatment and control group and administrative data, study authors reported that the sample consisted of all incomes. Mean age, predominant race, and gender balance was not reported. The study author reported saving amount and purchase asset outcomes, generated upon completion of filing the tax return. The study author did not provide sufficient data to calculate effect sizes. For saving amount, study authors report that treatment group members saved an average of $28.21, compared to a control group mean of $12.95. For purchase asset outcomes, study author report that 7% of treatment participants purchased an asset compared to 0.74% of control group participants. The review authors found that the risk of bias is high for this study for sequence generation (quasi‐experimental design), and allocation concealment (group assignment was based on site). The risk of bias is low for blinding of outcome assessors, incomplete outcome data, and other (research allegiance, funding, etc.). The risk of bias for blinding of participants and personnel and selective outcome reporting is unknown.

## 6 DISCUSSION

### 6.1 Summary of main results

The societal increase in individual choice and responsibility for selecting appropriate and beneficial financial products and services includes increased risk of making poor choices. Within this context, financial capability interventions are taking on increasing importance. The purpose of this systematic review was to determine the state of research and examine effects of financial capability interventions on selected populations to increase beneficial financial behaviours and financial outcomes.

This review included a total of 24 unique studies. The studies reported on a wide range of types of financial capability interventions that all included financial education and access to a financial product or service.

The largest category of studies related to financial education, counselling and coaching, followed closely by tax refund saving and investment, retirement, and matched savings accounts for adults. The studies also included CDAs, youth bank accounts, and homeownership education.

Overall, the evidence of the effects of financial capability interventions on financial behaviour and financial outcomes is sparse, given the lack of standardization of outcomes studied, measurement of the outcomes, or timing of measurement. Moreover, there were either only one or very few studies that examined effects of similar types of interventions on similar outcomes, thus we were not able to pool effects of studies to conduct a meta‐analysis. We were unable to calculate the effect sizes for the majority of the outcomes in the studies. For those we were able to calculate, effect sizes varied from small to moderate to large. The vast majority of large effect sizes were on account opening outcomes, with a few studies able to demonstrate large effects for saving amount, budgeting, and lowering debt. The studies with large effect sizes for account opening and saving amounts were CDAs and retirement account studies, while one debt management intervention resulted in large effect sizes for budgeting and lowering debt.

The studies that are available have important strengths. The majority of the studies are RCT's (*k* = 17) and many examine outcomes across several years. While many of the primary studies were RCTs, several of the sub‐studies used subsamples, thus those sub‐studies may introduce additional bias depending on how those subsamples were selected.

The risk of bias varies across studies. In addition, a number of the studies were assessed as high risk of bias for attrition and comparison groups differing on important demographic characteristics. Because the included studies did not have pre‐registered protocols, it is difficult to assess reporting bias for incomplete outcome data for all outcomes or selective outcome reporting. There is a general absence of information related to the blinding of participants or personnel, as well as other indicators of bias.

While the number and quality of studies included in this review precludes providing a clear answer on the effects of financial capability interventions, this review provides a good indicator of the state of the evidence for financial capability interventions and identified several important gaps to inform future research.

### 6.2 Overall completeness and applicability of evidence

While the evidence for each type of financial capability intervention indicates effectiveness of the intervention, the evidence across the studies on the effectiveness of financial capability interventions is incomplete.

While the majority of the studies used random assignment (72%), many of the studies had some important methodological weakness. For example, the majority of studies did not use a manualized intervention or study fidelity. Many of the studies experienced high attrition (more than 20%) in the control and treatment groups.

Many of the studies had a high risk of bias on most items. Because the included studies did not have pre‐registered protocols, it is difficult to assess reporting bias for incomplete outcome data for all outcomes or selective outcome reporting. Few of the included studies reported employed blinding of participants or personnel. With a few exceptions, there is a general absence of information related to the study authors' role in the interventions or potential bias stemming from study funding, thus we could not assess potential bias related to researcher allegiance or funding. Few studies had information about allocation concealment, or blinding of outcome assessors for all outcomes.

This review identified several different types of previously evaluated financial capability interventions. Unfortunately, few interventions were evaluated by more than one study that measured the same or similar outcomes, thus there were not a sufficient number of studies of any of the included intervention types that could be pooled to conduct a meta‐analysis. Therefore, evidence is sparse about whether participants' financial behaviours and/or financial outcomes are improved after receiving an intervention that met criteria for this review.

#### 6.2.1 Quality of the evidence

While the majority of the studies used random assignment (71%), many of the studies had some important methodological weakness. For example, the majority of studies did not use a manualized intervention or examine fidelity. We did not find protocols for the included studies, few of the included studies reported using blinding, and the majority did not report on allocation concealment nor on the researchers' roles in intervention development or implementation. Several of the studies experienced high attrition (more than 20%) in the control and treatment groups. Few assessed whether treatment or control group participants previously had financial education. Several large longitudinal studies also assessed outcomes on subgroups taken post‐hoc from the full sample, thus compromising random assignment.

### 6.3 Limitations and potential biases in the review process

The conduct and reporting of this review were guided by Campbell's standards and policies for the conduct and reporting of systematic reviews to ensure a rigorous and transparent review designed to minimize bias and error. This review is not without its limitations, however, and the findings must be interpreted in light of the study's limitations. We made every attempt to search for published and unpublished studies; however, the majority of the studies included in this review were published journal articles, although there were several white papers, research reports and dissertations included. The included studies presented with various risks of bias and thus caution must be used when interpreting findings. Moreover, a significant proportion of included studies were derived from just six large longitudinal experimental studies, thus while there are a relatively large number of studies in this review, many of them explore effects of a handful of interventions at different time points on different outcomes. Also, because the included studies were assessing effects of various types of interventions measuring conceptually different outcomes, and at different time points, there were not a sufficient number of studies to conduct meta‐analysis. Despite these limitations, this review provides a comprehensive summary of financial capability interventions that combine financial education and financial products and services from mainstream financial institutions (i.e., financial capability), shedding light on the promise and limitations of the body of evidence of these interventions, as well as the gaps in this area to inform future research.

### 6.4 Agreements and disagreements with other studies or reviews

This is the first systematic review of financial capability interventions that include both financial education and access to a financial product or service. Therefore, we are unable to comment on the degree to which this review agrees with similar reviews or studies.

## 7 AUTHORS' CONCLUSIONS

### 7.1 Implications for practice and policy

The lack of strong evidence about the effectiveness of financial capability intervention has implications for practice and policy. The diversity of financial capability interventions results in these types of interventions being delivered by practitioners from various professional backgrounds and in many different types of settings with various target audiences and goals. However, practitioners seek evidence on the effectiveness of their choice of interventions so they can deliver the most effective and efficient intervention towards their goals. While each type of financial capability intervention has a unique evidence base, this lack of evidence across financial capability intervention points to the need to develop a more definitive evidence base about whether the combination of financial education and financial access provides benefits to clients on specific outcomes.

In addition, policy actors that seek to facilitate increased financial capability through the interventions included in this review can seek a stronger evidence foundation for the effectiveness of financial capability interventions. Policy related to each of the interventions, including matched savings account, children's savings account, and the others, resides in various federal, state and local governmental bodies and nonprofit organizations. Organization among these policy actors to promote standardization of outcome measures, measurement time points post‐intervention, and other aspects of the service delivery and research methods across financial capability interventions would assist in the development of an evidence base. Policy work can facilitate additional research on financial capability interventions using strong methodology, manualized interventions and common outcomes would assist to build strong evidence.

### 7.2 Implications for research

While these interventions, the combination of providing a financial product along with education, have good intentions and would seem to be more effective than providing either individually, the evidence to date on the effectiveness of financial capability interventions is sparse and relatively weak. While many of these studies are large longitudinal studies with larger sample sizes, several of them lack the rigour and reporting of necessary information and data to be useful for informing the evidence base. Moreover, many of these large longitudinal studies are reported in multiple reports or sub‐studies, thus giving the impression that the evidence‐base is larger than it actually is. This body of research could be improved and more useful for informing practitioners and policy makers with some additional methodological and reporting rigour. Specifically, the publishing of protocols would provide additional accountability and rigor to the studies, and reporting all outcomes with sufficient data would allow for greater transparency and greater likelihood in results being able to be pooled in meta‐analysis. Developing a common set of outcomes that could be measured and reported across all studies examining effects of financial capability interventions could also assist in building and synthesizing the evidence on financial capability interventions.

## ROLES AND RESPONSIBILITIES

Please give brief description of content and methodological expertise within the review team. The recommended optimal review team composition includes at least one person on the review team who has content expertise, at least one person who has methodological expertise and at least one person who has statistical expertise. It is also recommended to have one person with information retrieval expertise:

- Content: Julie Birkenmaier and Youngmi Kim
- Systematic review methods: Brandy Maynard
- Statistical analysis: Brandy Maynard
- Information retrieval: Brandy Maynard

### SOURCES OF SUPPORT

This review had no source of financial support.

### DECLARATIONS OF INTEREST

Two of the review authors (Julie Birkenmaier and Youmgi Kim) are also co‐authors of some included studies. These authors did not extract data, code data, or critically appraise data from any study which they co‐authored. Further, the authors have no vested interest in the interventions or outcomes of this review, nor any incentive to represent findings in a biased manner.

### PLANS FOR UPDATING THE REVIEW

The review authors will examine the financial capability literature every 5 years to determine whether this review needs updating.

### DATA AND ANALYSES

None.

## Supporting information

Supporting information.
