# Supplementary material for: Interventions designed to improve financial capability: A systematic review
Source: Campbell Syst Rev. 2022 Mar 14;18(1):e1225. doi: 10.1002/cl2.1225 (PMC8919701; doi:10.1002/cl2.1225)
Supplement: Supplementary file 1 — Supporting information. [file CL2-18-e1225-s001.docx]

# Appendices

# Appendix A

## Documentation of search strategies in electronic databases

| **Database**  **(host)** | **Dates of Coverage** | **Search Fields** | **Country** | **Search Terms** |
| --- | --- | --- | --- | --- |
| ABI/INFORM  (ProQuest) | Round 1 -1900-May 2017  Round 2 May 2017-May 2020 | Abstracts  Abstracts | US | (financial OR economic OR bank) AND (education OR knowledge OR literacy) AND (capability OR access OR inclusion OR exclusion OR attachment) AND (evaluation OR intervention OR treatment OR outcome OR program OR trial OR experiment OR “control group” OR “controlled trial” OR “quasi-experiment” OR random*)  “Individual Development Accounts” OR “Child Development Accounts” OR “credit counseling” OR (“pre-purchase” AND home) OR (“pre-purchase” AND house) OR (“second chance” AND accounts) OR (“2^nd^ chance” AND accounts) OR (workplace OR “employer-sponsored” AND retirement OR savings) OR (“financial education” OR “financial counseling” OR “financial coaching” AND account) AND (evaluation OR intervention OR treatment OR outcome OR program OR trial OR experiment OR “control group” OR “controlled trial” OR “quasi-experiment” OR random*) |
| Academic Search Complete  (EBSCO) | Round 1 -1887-May 2017  Round 2 May 2017-May 2020 | Abstract or Author-Supplied Abstract  All text  Abstracts | US | (financial OR economic OR bank) AND (capability OR access OR inclusion OR exclusion OR attachment) AND (evaluation OR intervention OR treatment OR outcome OR program OR trial OR experiment OR “control group” OR “controlled trial” OR “quasi-experiment” OR random*)  (“financial education” OR “financial counseling” OR “financial coaching” AND account) AND (evaluation OR intervention OR treatment OR outcome OR program OR trial OR experiment OR “control group” OR “controlled trial” OR “quasi-experiment” OR random*)  “Individual Development Account” OR “Child Development Account” OR “credit counseling” OR (“pre-purchase” AND home) OR (“pre-purchase” AND house) OR (“second chance” AND accounts) OR (“2^nd^ chance” AND accounts) OR (workplace OR “employer-sponsored” AND retirement OR savings) AND (evaluation OR intervention OR treatment OR outcome OR program OR trial OR experiment OR “control group” OR “controlled trial” OR “quasi-experiment” OR random*) |
| Bloomberg Professional Service | *Round 1 – varies - May 2017) | Abstracts  Abstracts | US | (financial OR economic OR bank) AND (education OR knowledge OR literacy) AND (capability OR access OR inclusion OR exclusion OR attachment) AND (evaluation OR intervention OR treatment OR outcome OR program OR trial OR experiment OR “control group” OR “controlled trial” OR “quasi-experiment” OR random*)  “Individual Development Account” OR “Child Development Account” OR “credit counseling” OR (“pre-purchase” AND home) OR (“pre-purchase” AND house) OR (“second chance” AND accounts) OR (“2^nd^ chance” AND accounts) OR (workplace OR “employer-sponsored” AND retirement OR savings) OR (“financial education” OR “financial counseling” OR “financial coaching” AND account) AND (evaluation OR intervention OR treatment OR outcome OR program OR trial OR experiment OR “control group” OR “controlled trial” OR “quasi-experiment” OR random*) |
| Business Source Premier  (EBSCO) | Round 1 – 1965- May 2017  Round 2 May 2017-May 2020 | Abstracts or Author-supplied abstracts  Abstracts or Author-supplied abstracts | US | (financial OR economic OR bank) AND (education OR knowledge OR literacy) AND (capability OR access OR inclusion OR exclusion OR attachment) AND (evaluation OR intervention OR treatment OR outcome OR program OR trial OR experiment OR “control group” OR “controlled trial” OR “quasi-experiment” OR random*)  “Individual Development Account” OR “Child Development Account” OR “credit counseling” OR (“pre-purchase” AND home) OR (“pre-purchase” AND house) OR (“second chance” AND accounts) OR (“2^nd^ chance” AND accounts) OR (workplace OR “employer-sponsored” AND retirement OR savings) OR (“financial education” OR “financial counseling” OR “financial coaching” AND account) AND (evaluation OR intervention OR treatment OR outcome OR program OR trial OR experiment OR “control group” OR “controlled trial” OR “quasi-experiment” OR random*) |
| Database of Research on International Education  (Australian Council for Educational Research) | Round 1 – 1990 - May 2017  Round 2 May 2017-May 2020 | (No field choices provided)  (No field choices provided) | AUS | (financial OR economic OR bank) AND (education OR knowledge OR literacy) AND (capability OR access OR inclusion OR exclusion OR attachment) AND (evaluation OR intervention OR treatment OR outcome OR program OR trial OR experiment OR “control group” OR “controlled trial” OR “quasi-experiment” OR random*)  “Individual Development Account” OR “Child Development Account” OR “credit counseling” OR (“pre-purchase” AND home) OR (“pre-purchase” AND house) OR (“second chance” AND accounts) OR (“2^nd^ chance” AND accounts) OR (workplace OR “employer-sponsored” AND retirement OR savings) OR (“financial education” OR “financial counseling” OR “financial coaching” AND account) AND (evaluation OR intervention OR treatment OR outcome OR program OR trial OR experiment OR “control group” OR “controlled trial” OR “quasi-experiment” OR random*) |
| Dissertation & Thesis Global  (ProQuest) | Round 1 – 1770 - May 2017  Round 2 May 2017-May 2020 | Abstracts  Anywhere  Abstracts | US | (financial OR economic OR bank) AND (capability OR access OR inclusion OR exclusion OR attachment) AND (evaluation OR intervention OR treatment OR outcome OR program OR trial OR experiment OR “control group” OR “controlled trial” OR “quasi-experiment” OR random*)  (“financial education” OR “financial counseling” OR “financial coaching” AND account) AND (evaluation OR intervention OR treatment OR outcome OR program OR trial OR experiment OR “control group” OR “controlled trial” OR “quasi-experiment” OR random*)  “Individual Development Account” OR “Child Development Account” OR “credit counseling” OR (“pre-purchase” AND home) OR (“pre-purchase” AND house) OR (“second chance” AND accounts) OR (“2^nd^ chance” AND accounts) OR (workplace OR “employer-sponsored” AND retirement OR savings) AND (evaluation OR intervention OR treatment OR outcome OR program OR trial OR experiment OR “control group” OR “controlled trial” OR “quasi-experiment” OR random*) |
| EconLit  (EBSCO) | Round 1 – 1886 - May 2017  Round 2 May 2017-May 2020 | Abstracts  Abstracts | US | (financial OR economic OR bank) AND (education OR knowledge OR literacy) AND (capability OR access OR inclusion OR exclusion OR attachment) AND (evaluation OR intervention OR treatment OR outcome OR program OR trial OR experiment OR “control group” OR “controlled trial” OR “quasi-experiment” OR random*)  “Individual Development Account” OR “Child Development Account” OR “credit counseling” OR (“pre-purchase” AND home) OR (“pre-purchase” AND house) OR (“second chance” AND accounts) OR (“2^nd^ chance” AND accounts) OR (workplace OR “employer-sponsored” AND retirement OR savings) OR (“financial education” OR “financial counseling” OR “financial coaching” AND account) AND (evaluation OR intervention OR treatment OR outcome OR program OR trial OR experiment OR “control group” OR “controlled trial” OR “quasi-experiment” OR random*) |
| Education Source  (EBSCO) | Round 1 -May 2017  Round 2 May 2017-May 2020 | Abstracts  Abstracts | US | (financial OR economic OR bank) AND (education OR knowledge OR literacy): AND (capability OR access OR inclusion OR exclusion OR attachment) AND (evaluation OR intervention OR treatment OR outcome OR program OR trial OR experiment OR “control group” OR “controlled trial” OR “quasi-experiment” OR random*)  “Individual Development Account” OR “Child Development Account” OR “credit counseling” OR (“pre-purchase” AND home) OR (“pre-purchase” AND house) OR (“second chance” AND accounts) OR (“2^nd^ chance” AND accounts) OR (workplace OR “employer-sponsored” AND retirement OR savings) OR (“financial education” OR “financial counseling” OR “financial coaching” AND account) AND (evaluation OR intervention OR treatment OR outcome OR program OR trial OR experiment OR “control group” OR “controlled trial” OR “quasi-experiment” OR random*) |
| ERIC  (EBSCO) | Round 1 -May 2017  Round 2 May 2017-May 2020 | All text  Abstracts | US | (financial OR economic OR bank) AND (education OR knowledge OR literacy) AND (capability OR access OR inclusion OR exclusion OR attachment) AND (evaluation OR intervention OR treatment OR outcome OR program OR trial OR experiment OR “control group” OR “controlled trial” OR “quasi-experiment” OR random*)  “Individual Development Account” OR “Child Development Account” OR “credit counseling” OR (“pre-purchase” AND home) OR (“pre-purchase” AND house) OR (“second chance” AND accounts) OR (“2^nd^ chance” AND accounts) OR (workplace OR “employer-sponsored” AND retirement OR savings) OR (“financial education” OR “financial counseling” OR “financial coaching” AND account) AND (evaluation OR intervention OR treatment OR outcome OR program OR trial OR experiment OR “control group” OR “controlled trial” OR “quasi-experiment” OR random*)” |
| JSTOR | Round 1 – varies - May 2017  Round 2 May 2017-May 2020 | All fields  All fields  All fields | US | financial AND ((education OR knowledge OR literacy) AND (evaluation OR intervention OR treatment OR outcome OR program OR trial OR experiment OR “control group” OR “controlled trial” OR “quasi-experiment” OR random*))  “Individual Development Accounts” OR “Child Development Accounts” OR “credit counseling” OR (“pre-purchase” AND home) OR (“pre-purchase” AND house) AND retirement OR savings)  sponsored” AND retirement OR savings, and “financial education”  (“financial education” OR “financial counseling” OR “financial coaching” AND account) OR  (“second chance” AND accounts) OR (“2^nd^ chance” AND accounts) OR (workplace OR “employer-sponsored” AND “financial education” |
| PAIS Index  (ProQuest) | Round 1 – 1915 - May 2017  Round 2 May 2017-May 2020 | Abstracts  Abstracts | US | (financial OR economic OR bank) AND (education OR knowledge OR literacy) AND (capability OR access OR inclusion OR exclusion OR attachment) AND (evaluation OR intervention OR treatment OR outcome OR program OR trial OR experiment OR “control group” OR “controlled trial” OR “quasi-experiment” OR random*)  “Individual Development Account” OR “Child Development Account” OR “credit counseling” OR (“pre-purchase” AND home) OR (“pre-purchase” AND house) OR (“second chance” AND accounts) OR (“2^nd^ chance” AND accounts) OR (workplace OR “employer-sponsored” AND retirement OR savings) OR (“financial education” OR “financial counseling” OR “financial coaching” AND account) AND (evaluation OR intervention OR treatment OR outcome OR program OR trial OR experiment OR “control group” OR “controlled trial” OR “quasi-experiment” OR random*) |
| PsychINFO  (EBSCO) | Round 1 -May 2017  Round 2 May 2017-May 2020 | Abstracts  Abstracts | US | (financial OR economic OR bank) AND (education OR knowledge OR literacy) AND (capability OR access OR inclusion OR exclusion OR attachment) AND (evaluation OR intervention OR treatment OR outcome OR program OR trial OR experiment OR “control group” OR “controlled trial” OR “quasi-experiment” OR random*)  “Individual Development Account” OR “Child Development Account” OR “credit counseling” OR (“pre-purchase” AND home) OR (“pre-purchase” AND house) OR (“second chance” AND accounts) OR (“2^nd^ chance” AND accounts) OR (workplace OR “employer-sponsored” AND retirement OR savings) OR (“financial education” OR “financial counseling” OR “financial coaching” AND account) AND (evaluation OR intervention OR treatment OR outcome OR program OR trial OR experiment OR “control group” OR “controlled trial” OR “quasi-experiment” OR random*) |
| Public Affairs Index  (EBSCO) | Round 1 – 1937 - May 2017  Round 2 May 2017-May 2020 | Abstracts  Abstracts | US | (financial OR economic OR bank) AND (education OR knowledge OR literacy) AND (capability OR access OR inclusion OR exclusion OR attachment) AND (evaluation OR intervention OR treatment OR outcome OR program OR trial OR experiment OR “control group” OR “controlled trial” OR “quasi-experiment” OR random*)  “Individual Development Account” OR “Child Development Account” OR “credit counseling” OR (“pre-purchase” AND home) OR (“pre-purchase” AND house) OR (“second chance” AND accounts) OR (“2^nd^ chance” AND accounts) OR (workplace OR “employer-sponsored” AND retirement OR savings) OR (“financial education” OR “financial counseling” OR “financial coaching” AND account) AND (evaluation OR intervention OR treatment OR outcome OR program OR trial OR experiment OR “control group” OR “controlled trial” OR “quasi-experiment” OR random*) |
| Social Science Citation Index  (Web of Science) | Round 1 – 1990 - May 2017  Round 2 May 2017-May 2020 | Topic  Topic | US | (financial OR economic OR bank) AND (education OR knowledge OR literacy): AND (capability OR access OR inclusion OR exclusion OR attachment) AND (evaluation OR intervention OR treatment OR outcome OR program OR trial OR experiment OR “control group” OR “controlled trial” OR “quasi-experiment” OR random*)  “Individual Development Account” OR “Child Development Account” OR “credit counseling” OR (“pre-purchase” AND home) OR (“pre-purchase” AND house) OR (“second chance” AND accounts) OR (“2^nd^ chance” AND accounts) OR (workplace OR “employer-sponsored” AND retirement OR savings) OR (“financial education” OR “financial counseling” OR “financial coaching” AND account) AND (evaluation OR intervention OR treatment OR outcome OR program OR trial OR experiment OR “control group” OR “controlled trial” OR “quasi-experiment” OR random*) |
| Social Sciences Research Index  (ProQuest) | Round 1 – 1994 - May 2017  Round 2 May 2017-May 2020 | Title, abstract, keywords, full text  Title, abstract, keywords, full text | US | (financial OR economic OR bank) AND (education OR knowledge OR literacy) AND (capability OR access OR inclusion OR exclusion OR attachment) AND (evaluation OR intervention OR treatment OR outcome OR program OR trial OR experiment OR “control group” OR “controlled trial” OR “quasi-experiment” OR random*)  “Individual Development Account” OR “Child Development Account” OR “credit counseling” OR (“pre-purchase” AND home) OR (“pre-purchase” AND house) OR (“second chance” AND accounts) OR (“2^nd^ chance” AND accounts) OR (workplace OR “employer-sponsored” AND retirement OR savings) OR (“financial education” OR “financial counseling” OR “financial coaching” AND account) AND (evaluation OR intervention OR treatment OR outcome OR program OR trial OR experiment OR “control group” OR “controlled trial” OR “quasi-experiment” OR random*) |
| Social Work Abstracts  (EBSCO) | Round 1 – 1965 - May 2017  Round 2 May 2017-May 2020 | All text  Abstracts | US | (financial OR economic OR bank) AND (education OR knowledge OR literacy) AND (capability OR access OR inclusion OR exclusion OR attachment) AND (evaluation OR intervention OR treatment OR outcome OR program OR trial OR experiment OR “control group” OR “controlled trial” OR “quasi-experiment” OR random*)  “Individual Development Account” OR “Child Development Account” OR “credit counseling” OR (“pre-purchase” AND home) OR (“pre-purchase” AND house) OR (“second chance” AND accounts) OR (“2^nd^ chance” AND accounts) OR (workplace OR “employer-sponsored” AND retirement OR savings) OR (“financial education” OR “financial counseling” OR “financial coaching” AND account) AND (evaluation OR intervention OR treatment OR outcome OR program OR trial OR experiment OR “control group” OR “controlled trial” OR “quasi-experiment” OR random*) |
| Sociological Abstracts  (ProQuest) | Round 1 – 1952 - May 2017  Round 2 May 2017-May 2020 | Abstract | US | financial OR economic OR bank) AND (education OR knowledge OR literacy) AND (capability OR access OR inclusion OR exclusion OR attachment) AND (evaluation OR intervention OR treatment OR outcome OR program OR trial OR experiment OR “control group” OR “controlled trial” OR “quasi-experiment” OR random*)  “Individual Development Account” OR “Child Development Account” OR “credit counseling” OR (“pre-purchase” AND home) OR (“pre-purchase” AND house) OR (“second chance” AND accounts) OR (“2^nd^ chance” AND accounts) OR (workplace OR “employer-sponsored” AND retirement OR savings) OR (“financial education” OR “financial counseling” OR “financial coaching” AND account) AND (evaluation OR intervention OR treatment OR outcome OR program OR trial OR experiment OR “control group” OR “controlled trial” OR “quasi-experiment” OR random*) |

Note: Search dates for all searches were set to the beginning of the database to May, 2020 unless otherwise noted

*Unable to access database due to it’s restricted access during COVID-19 pandemic

**Appendix B: SCREENING FORM**

**Interventions Designed to Improve Financial Capability by Improving Financial Behavior and Financial Access: A Systematic Review**

1. Study ID#: __ __ __ [STID]

2. Date of Screening: __ __- __ __- __ __ __ __ [SCDATE]

3. Primary Author: ____________________________ [AUTH]

4. Bibliographic info (APA format): [BIB]

5. Is this study a: [STYPE]

- - - - - 1. RCT
        - 2. QED with parallel cohort
        - 3. None of the above- IF CHECKED THEN STOP

6. Was this study conducted in one of the 35 member OECD countries? [OECD]

- - - - - 0. No- STOP
        - 1. Yes
        - 2. Unsure

7. Is this a study that examines the effectiveness of interventions that use a combination of financial education or information, and access to a financial product or service?

[INTVNT]

- - - - - 0. No- STOP
        - 1. Yes
        - 2. Unsure

8. Does this study report at least one of the following outcomes: behavior change or result from behavior change as defined in the protocol? [OUTCOME]

- - - - - 0. No- STOP
        - 1. Yes

9. Is this study eligible for the review? [ELIG]

- - - - - 0. No: Reason _______________________
        - 1. Yes
        - 2. Need more information to make decision

10. Notes/Comments [NOTE]

## APPENDIX C: CODING FORM

**Interventions Designed to Improve Financial Capability by Improving Financial Behavior and Financial Access: A Systematic** **Review**

**Data Coding Form**

Study ID#: __________ Coder: ____________ Date of coding: ______________

Country: [country]

- 1. USA
- 2. Canada
- 3. Australia
- 4. Europe
- 5. Another OECD country : _________________

Report Type: [rtype]

- 1. Journal Article
- 2. Book/book chapter
- 3. Gov't report (local, state, federal)
- 4. Conference proceedings
- 5. Thesis or Dissertation
- 6. Unpub report (non-gov’t, tech report) and other
- 7. Research brief
- 8. Expert Referral

**Methods**

Method of assignment to condition: [grp_assign]

- 1. Random, simple
- 2. Random, after matching, stratification, blocking, etc.
- 3. Quasi-random-assigned by some naturally occurring process
- 4. QED with parallel cohorts
- 99. Not specified / not enough information to determine

Results of statistical comparisons of pretest differences on outcomes: [grp_pre]

- - 1. No (statistical) comparisons made
  - 2. No statistically significant differences
  - 3. Statistically significant differences found

Results of statistical comparisons of pretest differences on demographics (race, ethnicity, income?): [grp_pre2]

- - 1. No (statistical) comparisons made
  - 2. No statistically significant differences
  - 3. Statistically significant differences found

Unit of assignment to conditions: [txassign]

- - 1. Individual participant
  - 2. Group/Cluster: specify __________________
  - 3. Other: _______________________
  - 99. Not enough information to determine

If groups were non-equivalent at baseline, were statistical controls used? [grp_ctrl]

- 1. Yes
- 2. No
- 3. Were equivalent
- 99. Not enough information to determine

If matching was used, how were groups matched? [grp_match]

- 1. Matched on pretest measure
- 2. Matched on demographics
- 3. Matched on both of the above
- 4. Propensity Score Matching
- 5. Other matching technique: ____________________
- 7. Not enough information to determine
- 8. Were not matched

Financial education: [fin_ed]

- 1. Low-touch
- 2. High touch
- 999. Unable to determine

**Population**

Mean Age of participants: ___________

99. Not specified

Age of participants who are the target beneficiaries: [age2]

- 0. Infants (O-K)
- 1. Children - Elementary School (K-5)
- 2. Children - Middle school (6-8)
- 3. Children - High school (9-12)
- 4. Parents of children
- 5. Young adults (18-25 yrs old)
- 6. Adults (age undetermined)
- 7. Older adults
- 99. Not enough information to determine

Child Parental Rights: [fostcare]

- 1. N/A
- 2. In foster care

Predominant Race/Ethnicity: [raceth]

- 1. African American
- 2. Asian
- 3. Whites
- 4. Hispanic
- 99. Not specified

Sex: [sex]

- 1. Male___%
- 999. Not specified

Income: [inc]

- 1. Low Income
- 2. Low and Moderate Income
- 3. All incomes levels
- 4. Not Specified

Previous financial education: [prevfined]

- 1. No ___%
- 999. Not Specified

Intervention targeted people without access to: [prevfinacc]

- 1. Bank account
- 2. Checking Account
- 3. Retirement savings account
- 4. Home mortgage
- 5.Credit card
- 6. Education savings account
- 7. Other (specify): __________
- 8. Assets (for all IDA studies)
- 99. Not specified

Intervention setting: [Interset]

- Community-based non-profit
- 2. School
- 3. Preschool/Headstart
- 4. Government agency
- 5. For-profit business
- 6. University
- 7. Community (no physical setting)
- 8. Other: __________________
- 99. Not specified

Who provided the services? (Check all that apply) [intagt]

- 1. Non-profit staff
- 2. For-profit company staff
- 3. Government/public staff
- 4. Researchers
- 5. Other: __________________(list)
- 99. Not specified

Is the intervention targeted to a specific population? [int_tar]

- 1. Yes ___________
- 99. Not specified

**Interventions**

Stated goal/purpose of the intervention (check all that apply): [goal]

- 1. Increase emergency/short-term savings
- 2. Increase college savings
- 3. Increase retirement savings
- 4. Increase savings for investment (education, business, home)
- 5. Increase financial management skills
- 6. Increase engagement with bank/credit union
- 7. Increase engagement with low-cost credit/loan
- 8. Increase financial knowledge
- 9. Other _________________________
- 99. Not specified

What financial capability strategies characterize the intervention? [strategy]

(check all that apply)

- 1. Matched savings accounts combined with financial education (individual development accounts)
- 2. Low- or no-cost transaction accounts combined with financial education
- 3. Second-chance accounts combined with financial education
- 4. Youth accounts combined with financial education
- 5. Retirement accounts combined with financial education
- 6. Pre-purchase home buying education with financial education
- 7. CDAs with financial education
- 8. Credit counselling
- 9. Savings programs (or CDs) combined with financial education
  - - - - 10. Other- specify: ________________________

Role of the evaluator/author/research team or staff in the program: [arole]

- 1. Researcher independent of treatment- research role only
- 2. Researcher not independent of treatment
- 99. Not specified

Treatment Format (check all that apply): [format]

- 1. Individual (one-on-one) – customized tx of some kind
- 2. Group – same tx for all subjects in tx group(s)
- 3. Individual & Group Mix of standard and customized tx
- 4. Other: _____________
- 99. Not specified

Length of treatment (# of weeks): [length]

- 1. Specified: _________
- 99. Not specified

Frequency of contact: [freq]

- 1. Once
- 2. Periodically/irregular
- 3. Per week: ____________
- 4. Per month: ___________
- 99. Not specified

Total # of sessions: ______________ [sessions]

- 1. Specified: _________
- 99. Not specified

Is this intervention manualized? ______ [manual]

- 1. Yes _______
- 99. Not specified

Did the study measure fidelity? [fidel]

- 1. Yes
- 2. No

How was fidelity assessed? [fidel_asses]

- 1. Researcher observations
- 2. Interviews of participants
- 3. Surveys of participants
- 4. Participant logs
- 5. Administrative records
- 6. Checklists
- 7. Other ______________________
- 99. Not specified

Did the treatment group have high attrition (for RCT/QED > 20%)? [grp_attrit]

- 1. Yes
- 2. No
- 99. Not enough information to calculate

What did the control/comparison group receive? [compcond]

- 1. Nothing or wait list
- 2. “Treatment as usual”: Specify _____________________
- 3. Specified treatment: Specify _____________________
- 4. Other: ______________________________

Did the control/comparison have high attrition (for RCT/QED > 20%)? [grp_attrit]

- 1. Yes
- 2. No
- 99. Not enough information to calculate

Timing of measurement of outcomes

Tx analytic sample size

Control group analytic sample size

**Outcomes**

(By intervention type) – see below

**Continuous and dichotomous outcomes**

| **Timing of measurement (during tx, end of tx, 3 month, etc.) CAN ADD if needed** | **Tx Baseline**  **Mean** | **Tx Baseline**  **SD** | **Tx baseline N** | **Tx Post**  **Mean** | **Tx Post SD** | **Tx Post N** | **Control group Baseline**  **Mean** | **Control group SD** | **Control group N** | **Values for t, F, other** |
| --- | --- | --- | --- | --- | --- | --- | --- | --- | --- | --- |
|  |  |  |  |  |  |  |  |  |  |  |
|  |  |  |  |  |  |  |  |  |  |  |
|  |  |  |  |  |  |  |  |  |  |  |
|  |  |  |  |  |  |  |  |  |  |  |

Continuous Construct ID: 1= Savings Amount; 2=Savings rate; 3=Fund balance; 4= Credit score; 5 = debt; 6 = value of asset; 7 = other

Dichotomous Construct ID: 1= Open account; 2= Active use of accounts; 3= Use new product

## APPENDIX D: EXCLUDED STUDIES (n=353)

| Study | Reason for Exclusion  (see screening tool for inclusion criteria) |
| --- | --- |
| Achieving the Dream/OneMain Financial. (2018). Partnering to ensure student financial stability. <http://ezp.slu.edu/login?url=http://search.ebscohost.com/login.aspx?direct=true&db=eric&AN=ED591921&site=ehost-live>  Administration for Children and Families, Office of Community Services. (2009). *Report to Congress: Assets for Independence Program Status at the Conclusion of the Tenth Year.* Washington, DC: U. S. Department of Health and Human Services. | Did not meet intervention criteria  Did not meet design criteria |
| Administration for Children and Families, Office of Community Services. (2010). *Report to Congress: Assets for Independence Program Status at the conclusion of the eleventh year,* Results through September 30, 2010*.* Washington, DC: U. S. Department of Health and Human Services. | Did not meet design criteria |
| Administration for Children and Families, Office of Community Services. (2011). *Report to Congress: Assets for Independence Program Status at the conclusion of the twelfth year,* Results through September 30, 2011*.* Washington, DC: U. S. Department of Health and Human Services. | Did not meet design criteria |
| Administration for Children and Families, Office of Community Services. (2012). *Report to Congress: Assets for Independence program status at the conclusion of the thirteenth year, Results through September 30, 2012.* Washington, DC: U. S. Department of Health and Human Services. | Did not meet design criteria |
| Administration for Children and Families, Office of Community Services. (2013). *Report to Congress: Assets for Independence program status at the conclusion for the fourteenth year,* Results through September 30, 2013*.* Washington, DC: U. S. Department of Health and Human Services. | Did not meet design criteria |
| Administration for Children and Families, Office of Community Services. (2014). *Report to Congress: Assets for Independence Program status at the conclusion of the fifteenth year,* Results through September 30, 2014. Washington, DC: U. S. Department of Health and Human Services. | Did not meet design criteria |
| Agarwal, S., Amromin, G., Ben-David, I., Chomsisengphet, S., & Evanoff, D. (2009). Learning to cope: Voluntary financial education programs and loan performance during a housing crisis. Ohio State University, Charles A. Dice Center for Research in Financial Economics, Working Paper Series.  Agarwal, S., Amromin, G., Ben-David, I., Chomsisengphet, S., & Evanoff, D. D. (2009). *Do financial counseling mandates improve mortgage choice and performance? Evidence from a legislative experiment* (No. 2009-07). Working Paper, Federal Reserve Bank of Chicago. | Did not meet design criteria  Did not meet design criteria |
| Alcon, A. M. (2000). *The efficacy of employer-sponsored financial education programs and activities: What impact on women's financial situation in old age?* (Order No. 9974021). Retrieved from ProQuest Dissertations & Theses Global. (304664906) | Did not meet design criteria |
| Allen, S. G., Clark, R. L., Maki, J., & Morrill, M. S. (2016). Golden years or financial fears? How plans change after retirement seminars. *Journal of Retirement*, *3*(3), 96-115. | Did not meet design criteria |
| Amagir, A., Groot, W., Maassen van den Brink, H., & Wilschut, A. (2019). SaveWise: The design of a financial education program in the Netherlands. *Citizenship, Social and Economics Education*, *18*(2), 100-120.  Ambuehl, S., Bernheim, B. D., Ersoy, F., & Harris, D. (2018). *Peer advice on financial decisions: A case of the blind leading the blind?* (No. W25034). National Bureau of Economic Research.  Anderson, C., & Card, K. (2015). Effective practices of financial education for college students: Students' perceptions of credit card use and financial responsibility. *College Student Journal*, *49*(2), 271-279.  Anderson, J. G., Uttley, C. M., & Kerbel, C. M. (2006). Outcomes of a workplace financial education program. *The Journal of Consumer Education, 23,* 37-49.  Anderson, S., Zhan, M., & Scott, J. (2007). Improving the knowledge and attitudes of low-income families about banking and predatory financial practices. *Families in Society: The Journal of Contemporary Social Services*, *88*(3), 443-452. | Did not meet intervention criteria  Did not meet intervention criteria  Did not meet intervention criteria  Did not meet  intervention criteria  Did not meet design criteria |
| Anderson, S., Scott, J., & Zhan, M. (2004). Financial links for low-income people (FLLIP): Final evaluation report. *School of Social Work, University of Illinois at Urbana-Champaign.* | Did not meet design criteria |
|  |  |
| Angel, S. (2018). Smart tools? A randomized controlled trial on the impact of three different media tools on personal finance. *Journal of Behavioral and Experimental Economics*, *74*, 104-111. | Did not meet design criteria |
| Archer, W., Fitterman, S., & Smith, M. T. (2009). Real Estate Brokerage, Homebuyer Training, and Homeownership Sustainability for Housing Assistance Programs. *Family and Consumer Sciences Research Journal*, *37*(4), 442-455.  Arnone, W. J. (2002). Financial planning for employees post-Enron. *Benefits Quarterly*, *18*(4), 35 | Did not meet intervention criteria  Did not meet design criteria |
|  |  |
| Atkinson, A. & Messy, F. (2013). *Promoting Financial Inclusion through Financial Education: OECD/INFE Evidence, Policies and Practice* (OECD Working Paper No. 34) Retrieved from <http://dx.doi.org/10.1787/5k3xz6m88smp-en> | Did not meet design criteria |
| Atkinson, A. & Messy, F. (2015). *Financial Education for Migrants and their Families.*(OECD Working Papers No. 38). Paris: OECD Publishing. Retrieved from <https://doi.org/10.1787/5js4h5rw17vh-en>. | Did not meet design criteria |
| Atkinson, A., Messy, F., Rabinovich, L. & Yoong, J. (2015). *Financial Education for Long-term Savings and Investments: Review of Research and Literature* (OECD Working Papers No. 39). Paris: OECD Publishing. Retrieved from <https://doi.org/10.1787/5jrtgzfl6g9w-en>. | Did not meet design criteria |
| Ansong, D., Chowa, G., Masa, R., Despard, M., Sherraden, M., Wu, S., & Osei-Akoto, I. (2019). Effects of youth savings accounts on school attendance and academic performance: evidence from a youth savings experiment. *Journal of Family and Economic Issues*, *40*(2), 269-281.  Argento, R. B., Brown, L. M., Koulayev, S., Li, G., Myhre, M., Pafenberg, F., & Patrabansh, S. (2019). First-time homebuyer counseling and the mortgage selection experience in the United States. *Cityscape*, *21*(2), 51-74.  Azurdia, G., & Freedman, S. (2016). Encouraging Nonretirement Savings at Tax Time: Final Impact Findings from the SaveUSA Evaluation. *New York: MDRC.* Retrieved from <https://www.mdrc.org/publication/encouraging-nonretirement-savings-tax-time> | Did not meet outcomes criteria  Did not meet design criteria  Did not meet intervention criteria |
| Banov, R. (2005). *The effect of health insurance on savings outcomes in Individual Development Accounts*(CSD Report 05-29).  St. Louis, MO: Washington University, Center for Social Development. | Did not meet design criteria |
| Barcellos, S. H., Carvalho, L. S., Smith, J. P., & Yoong, J. (2016). Financial education interventions targeting immigrants and children of immigrants: Results from a randomized control trial. *Journal of Consumer Affairs*, *50*(2), 263-285. | Did not meet intervention criteria |
| Barron, J. M., & Staten, M. E. (2011). *Is Technology-Enhanced Credit Counseling as Effective as In-Person Delivery?* (Working Paper No. 11-11). Philadelphia, PA: Research Department, Federal Reserve Bank of Philadelphia.  Bauer, R., Eberhardt, I., & Smeets, P. (2019). A fistful of dollars-financial incentives improves retirement information search. *Available at SSRN 3023943*. | Did not meet design criteria  Did not meet intervention criteria |
| Bayer, P. J., Bernheim, B. D., & Scholz, J. K. (2009). The effects of financial education in the workplace: Evidence from a survey of employers. *Economic Inquiry*, *47*(4), 605-624. | Did not meet design criteria |
| Bernheim, D. (1995). Do households appreciate their financial vulnerabilities? An analysis of actions, perceptions, and public policy. In M. B. Kotowski (Ed.), *Tax policy and economic growth*, (pp. 1-30). | Did not meet design criteria |
| Bernheim, D. D. (1998). Financial illiteracy, education, and retirement saving. In O.S. Mitchell & S. Schiber (Eds.), *Living with Defined Contribution Pensions.* Philadelphia: University of Pennsylvania Press. (pp. 36-68). | Did not meet design criteria |
| Bernheim, B. D. & Garrett, D. M. (1996). The determinants and consequences of financial education in the work place: Evidence from a survey of households (Working Paper No. 5667). National Bureau of Economic Research. | Did not meet design criteria |
| Bernheim, B. D. & Garrett, D. M. (2003). The effects of financial education in the workplace: Evidence from a survey of households. *Journal of Public Economics, 87*(7-8). 1487-1519. DOI: 10.1016/S0047-2727(01)00184-0. | Did not meet design criteria |
| Bhattacharya, R., Gill, A., & Stanley, D. (2016). The effectiveness of financial literacy instruction: The role of individual development accounts participation and the intensity of instruction. *Journal of Financial Counseling and Planning*, *27*(1), 20-35. | Did not meet outcomes criteria |
| Birkenmaier, J. M. (2006). *Providing access to affordable credit to the poor: Evaluating the effectiveness of credit counseling for building assets*(Order No. 3209943). Retrieved from ProQuest Dissertations & Theses Global. (304961606). | Did not meet design criteria |
| Birkenmaier, J., & Tyuse, S. W. (2006). Does homeownership education and counseling (HEC) help credit scores?. *Journal of Social Service Research*, *32*(2), 81-103. | Did not meet design criteria |
| Black, B. (2004). *Evaluation of the Individual Development Account Program for the New Hampshire Community Loan Fund*. Concord, NH: Betsy Black Consulting, LLC. | Did not meet design criteria |
| Blumenthal, A., & Shanks, T. R. (2019). Communication matters: A long-term follow-up study of child savings account program participation. *Children and Youth Services Review*, *100*, 136-146.  Bolanos, A. B. (2012). *Key determinants in building financial capability among middle schoolers with a school-based financial literacy education program*(Order No. 3533405). Retrieved from ProQuest Dissertations & Theses Global. (1221041706). | Did not meet outcomes criteria  Did not meet design criteria |
| Bolton, L. E., Bloom, P. N., & Cohen, J. B. (2011). Using loan plus lender literacy information to combat one-sided marketing of debt consolidation loans. *Journal of Marketing Research*, *48*(SPL), S51-S59.  Bover, O., Hospido, L., & Villanueva, E. (2018). *The impact of high school financial education on financial knowledge and choices: Evidence from a randomized trial in Spain*. Working Paper Series, Banco de Espana. | Did not meet design criteria  Did not meet intervention criteria |
| Boyer, M. M., d'Astous, P., & Michaud, P. C. (2019). *Tax-sheltered retirement accounts: Can financial education improve decisions?* (No. W26128). National Bureau of Economic Research.  Brown, S. (2010). Risk reduction and sustainable lending: The effect of pre-purchase homebuyer education on mortgage foreclosure. Vanderbilt University, Nashville, TN. | Did not meet intervention criteria  Did not meet design criteria |
| Brown, S. (2015). The influence of homebuyer education on default and foreclosure risk: A natural experiment. *Journal of Policy Analysis and Management, 35*(1), 145-172. DOI:10.1002/pam.21877.  Business West (2017). United Bank reports on PATH Plus program. *Chicopee, 34*(10), 74. | Did not meet design criteria  Did not meet design criteria |
| Burk, D. (2011). Evaluation of the USU Retirement and Savings Seminar. Master of Science Thesis in Family, Consumer, and Human Development. Logan, Utah: Utah State University. | Did not meet design criteria |
| Burke, L., & Bedrick, J. (2018). Personalizing education: How Florida families use education savings accounts. *EdChoice.* <http://ezp.slu.edu/login?url=http://search.ebscohost.com/login.aspx?direct=true&db=eric&AN=ED591348&site=ehost-live>  Burke, J., Jamison, J., Karlan, D., Mihaly, K., & Zinman, J. (2019). *Credit Building or Credit Crumbling? A Credit Builder Loan’s Effects on Consumer Behavior, Credit Scores and Their Predictive Power* (No. w26110). National Bureau of Economic Research. https://www.law.northwestern.edu/research-faculty/colloquium/law-economics/documents/karlanspring2020.pdf  Carbonell, S. Hicks, D. Savage, S. (2015). *Community college initiative: Helping students achieve financial stability by building their financial capability* (MDC Webinar). Boston, MA: Federal Reserve Bank of Boston. | Did not meet design criteria  Did not meet intervention criteria  Did not meet design criteria |
| Campbell, M. W. (2018). *Financial literacy among military for-profit undergraduate students: A quasi-experimental study* (Doctoral dissertation, University of Phoenix).  Carswell, A. T. (2009). Does housing counseling change consumer financial behaviors? Evidence from Philadelphia. *Journal of Family and Economic Issues*, *30*(4), 339. | Did not meet intervention criteria  Did not meet design criteria |
| Carswell, A. T. (2005). *Measuring the effectiveness outcomes of pre-purchase homeownership counseling recipients in the city of Philadelphia*(Order No. 3157282). Retrieved from ProQuest Dissertations & Theses Global. (304994037). | Did not meet design criteria |
| Chatterjee, S., Green-Pimentel, L., & Turner, P. (2010). Financial education and consumers’ willingness to change behavior. *Investment Management and Financial Innovations*, *7*(3), 73-81. | Did not meet design criteria |
| Chen, Z., Elliott, W., Wang, K., Zhang, A., & Zheng, H. (2020). Examining parental educational expectations in one of the oldest children’s savings account programs in the country: The Harold Alfond College Challenge. *Children and Youth Services Review*, *108*, 104582.  Child and Family Research Partnership. (2016). *Saving made simple: An evaluation of an employer-based automatic savings program in Austin, TX* (CFRP Policy Brief B.022.0316). The University of Texas at Austin Lyndon B. Johnson School of Public Affairs. | Did not meet design criteria  Did not meet design criteria |
| Choi, L. (2009). *Bank accounts and youth financial knowledge: Connecting experience and education* (Working paper 2009-07). San Francisco, US: The Federal Reserve Bank of San Francisco. Retrieved from http://www. frbsf.org/community | Did not meet design criteria |
| Choi, L. (2011). Prize Linked Accounts for Youth (PLAY): A new approach to youth financial education and saving. *Community Investments*, *23*(1), 2-8. | Did not meet design criteria |
| Choi, J. J., Laibson, D., Madrian, B. C., & Metrick, A. (2002). Defined contribution pensions: Plan rules, participant choices, and the path of least resistance. *Tax policy and the economy*, *16*, 67-113. | Did not meet design criteria |
| Choi J, Laibson D, Madrian B, Metrick A. [Saving for Retirement on the Path of Least Resistance](https://scholar.harvard.edu/laibson/publications/saving-retirement-path-least-resistance). In: E. McCaffrey and J, Slemrod (Eds.), *Behavioral Public Finance: Toward a New Agenda.* New York: Russell Sage Foundation. (pp. 304-351). | Did not meet design criteria |
| Christy-McMullin, K., Shobe, M. A., & Wills, J. (2008). Arkansas IDA programs: Examining asset retention and perceptions of well-being. *Journal of Social Service Research*, *35*(1), 65-76. | Did not meet design criteria |
| Clancy, M., Grinstein-Weiss, M., & Schreiner, M. (2001). Financial education and savings outcomes in Individual Development Accounts (CSD Working Paper 01-2). St. Louis, MO: Washington University, Center for Social Development. | Did not meet design criteria |
| Clancy, M., Schreiner, M., & Sherraden, M. (2002). The United Way of greater St. Louis Individual Development Account pilot program (CSD Report 02-18). St. Louis, MO: Washington University, Center for Social Development. | Did not meet design criteria |
| Clancy, M., & Sherraden, M. (2014). *Automatic deposits for all at birth: Maine’s Harold Alfond College Challenge*(CSD Policy Report 14-05). St. Louis, MO: Washington University, Center for Social Development. | Did not meet design criteria |
| Clark, R., d'Ambrosio, M., McDermed, A. A., Sawant, K. (2003, March). Financial education and retirement savings. In *Sustainable Community Development: What Works, What Doesn’t, and Why* (pp. 1-43)*.* Washington, DC: Federal Reserve System. | Did not meet design criteria |
| Clark, R. L., & d’Ambrosio, M. B. (2003). Ignorance is not bliss: The importance of financial education. *Research Dialogue, 78,* 1-14. | Did not meet design criteria |
| Clark, R., & d’Ambrosio, M. (2008). Adjusting retirement goals and saving behavior: The role of financial education. In A. Lusadi (Ed.), *Overcoming the saving slump: How to increase the effectiveness of financial education and saving programs* (pp. 237-256). Chicago: University of Chicago Press. | Did not meet design criteria |
| Clark, R., d’Ambrosio, M., McDermed, A., & Sawant, K. (2004). Sex differences, financial education and retirement goals. In O. S. Mitchell and S. P. Utkus (Eds.), *Pension design and structure: New lessons from behavioral finance* (pp. 185-206). New York, NY: Oxford University Press. | Did not meet design criteria |
| Clark, R. L., d'Ambrosio, M. B., McDermed, A. A., & Sawant, K. (2006). Retirement plans and saving decisions: the role of information and education. *Journal of Pension Economics & Finance*, *5*(1), 45-67. | Did not meet design criteria |
| Clark, R. L., Maki, J. A., & Morrill, M. S. (2014). Can simple informational nudges increase employee participation in a 401 (k) plan?. *Southern Economic Journal*, *80*(3), 677-701. | Did not meet design criteria |
| Clark, R. L., Morrill, M. S., & Allen, S. G. (2010, April). Employer-provided retirement planning programs. In *Annual Conference of the Pension Research Council (pp. 1-43).* Philadelphia, PA: University of Pennsylvania. | Did not meet design criteria |
| Clark, R. L., Morrill, M. S., & Allen, S. G. (2010). Evaluating workplace education for new hires. Presentation, North Carolina State University *Financial Literacy Center.* Retrieved from <https://regroup-production.s3.amazonaws.com/documents/reviewreference/38198501> | Did not meet design criteria |
| Clark, R. L., Morrill, M. S., & Allen, S. G. (2010). Pension plan distributions: The importance of financial literacy (PRC working paper 20110-27).  *Philadelphia, PA:* University of Pennsylvania, Pension Research Council. | Did not meet design criteria |
| Clark, R. L., & Schieber, S. J. (1998). Factors affecting participation rates and contribution levels in 401 (k) plans. In O. S. Mitchell and S. J. Schieber (Eds.), *Living with defined contribution pensions: Remaking responsibility for retirement*, (pp. 69-97). | Did not meet design criteria |
| Collard, S., & McKay, S. (2006). Closing the savings gap? The role of the Saving Gateway. *Local Economy*, *21*(1), 25-35. | Did not meet design criteria |
| Collins, J. M., Gjertson, L., & O’Rourke, C. (2016). MyBudgetCoach Pilot Evaluation. Center for Financial Security. Madison, WI: University of Wisconsin-Madison. | Did not meet design criteria |
| Consumer Financial Protection Bureau. (2014). *Financial Wellness at Work: A Review of Promising Practices and Policies.* Retrieved from <https://files.consumerfinance.gov/f/201408_cfpb_report_financial-wellness-at-work.pdf>  Curley, J. & Grinstein-Weiss, M. (2003). A comparative analysis of rural and urban saving performance in Individual Development Account programs. *Social Development Issues, 25*(1) 89-105. | Did not meet design criteria  Did not meet design criteria |
| Curley, J., & Robertson, A. S. (2014). *Head Start family financial capability: 2013–2014 annual report of the ASSET Project* (CSD Research Report 14-27). St. Louis, MO: Washington University, Center for Social Development. | Did not meet design criteria |
| Curley, J. C. (2004). *The role of institutions in the saving participation and performance of low -income households in individual development accounts*(Order No. 3147434). Retrieved from ProQuest Dissertations & Theses Global. (305108605). | Did not meet design criteria |
| Curley, J., Ssewamala, F., & Sherraden, M. (2009). Institutions and savings in low-income households. *Journal of Sociology & Social Welfare*, *36*(3), 9-32. | Did not meet design criteria |
| Delgadillo, L. (2015). Using Individual Development Accounts to sustain homeownership and foster financial skills, practices and self-efficacy. *Journal of Family and Consumer Sciences, 107*(3), 18-26.  Dare, S. E., van Dijk, W. W., van Dijk, E., van Dillen, L. F., Gallucci, M., & Simonse, O. (2020). The effect of financial education on pupils’ financial knowledge and skills: Evidence from a Solomon four-group design. *The Journal of Educational Research*, *113*(2), 93-107.  Davison, G. Frank-Miler, E., Roll, S.P. & Grinstein-Weiss, M. (2018). Promoting savings at tax time: Insights from online and in-person tax preparation services. Center for Social Development Research Report No. 18-33. | Did not meet design criteria  Did not meet intervention criteria  Did not meet outcomes criteria |
| DeMarco, A., DeMarco, M., Biggers, A., West, M., Young, J., & Levy, R. (2015). Can people experiencing homelessness acquire financial assets? *Journal of Sociology & Social Welfare*, *42*(4), 55-78. | Did not meet design criteria |
| DeMarco, D., Mills, G., & Ciurea, M. (2008). *Assets for Independence Act evaluation* (Final report). Retrieved from <https://www.acf.hhs.gov/sites/default/files/ocs/afi_final_process_study_0_0.pdf>  Despard, M., Grinstein-Weiss, M., Guo, S., Taylor, S., & Russell, B. (2018). Financial shocks, liquid assets, and material hardship in low-and moderate-income households: Differences by race. *Journal of Economics, Race, and Policy*, *1*(4), 205-216.  Despard, M. R., Grinstein‐Weiss, M., Ren, C., Guo, S., & Raghavan, R. (2017). Effects of a tax‐time savings intervention on use of Alternative Financial Services among lower‐income households. *Journal of Consumer Affairs*, *51*(2), 355-379. | Did not meet design criteria  Did not meet design criteria  Did not meet outcomes criteria |
| Despard, M., Zeng, Y., Fox-Dichter, S., Frank-Miller, E., & Grinstein-Weiss, M. (2020). Financial counseling for front-line workers: A pilot study of engagement and outcomes. Social Policy Institute at Washington University in St. Louis.  DeVaney, S. A., Gorham, L., Bechman, J. C., & Haldeman, V. (1995). Saving and investing for retirement: The effect of a financial education program. *Family Economics and Resource Management Biennial*, *21*, 71-80. | Did not meet intervention criteria  Did not meet design criteria |
| Dietz, N., de Leon, E., Fyffe, S., Kuehn, D., Gaddy, M., Collazos, J. (2016). *An assessment of the local initiatives support corporation’s Financial Opportunity Centers* (Research report). Retrieved from [https://www.urban.org/sites/default/files/publication/84276/2000933-An-Assessment-of-the-Local-Initiatives-Support-Corporation’s-Financial-Opportunity-Centers.pdf](https://www.urban.org/sites/default/files/publication/84276/2000933-An-Assessment-of-the-Local-Initiatives-Support-Corporation's-Financial-Opportunity-Centers.pdf) | Did not meet intervention criteria |
| Dolls, M., Doerrenberg, P., Peichl, A., & Stichnoth, H. (2016). *Do savings increase in response to salient information about retirement and expected pensions?* (NBER Working Paper No. 22684). Retrieved from <http://www.nber.org/papers/w22684> | Did not meet design criteria |
| Dolvin, S. D. & Templeton, W. K. (2006). Financial education and asset allocation. *Financial Services Review 15*(2006), 133-149. | Did not meet design criteria |
| Duflo, E. & Saez, E. (2002). The role of information and social interactions in retirement plan decisions: Evidence from a randomized experiment (Working Paper No. 8885). National Bureau of Economic Research. | Did not meet design criteria |
| Edmiston, K. D. & Gillett-Fisher, M. C. (2006). Financial education at the workplace (Working Paper No. 06-02). Federal Reserve Bank of Kansas City Community Affairs Department. | Did not meet design criteria |
| Edmiston, K. D., Gillet-Fisher, M. C., & McGrath, M. (2009) Weighing the effects of financial education in the workplace (Working Paper No. 09-01). The Federal Reserve Bank of Kansas City Community Affairs Department. | Did not meet design criteria |
| Edwards, A. M. (2013). A quantitative quasi-experimental retrospective study of microenterprise development programs and low-income female micro entrepreneurs. Doctoral Dissertation, University of Phoenix, Phoenix, AZ. | Did not meet design criteria |
| Elliehausen, G., Lundquist, E. C., & Staten, M. E. (2007). The impact of credit counseling on subsequent borrower behavior. *The Journal of Consumer Affairs, 41*(1), 1-28. | Did not meet design criteria |
| Elliott, W. & Kim, J.S. (2013). The role of identity-based motivation and solution-focus brief therapy in unifying accounts and financial education in school-related CDA programs. *Children and Youth Service Review, 35*(2013)*,* 402-410. | Did not meet design criteria |
| Eschbach, C. L., Weber, R., Tobe, E., Hale, L., & Washington, V. (2016). Evaluating an Outcomes‐based Standardized Homeownership Education Program. *Family and Consumer Sciences Research Journal*, *45*(2), 138-149. | Did not meet design criteria |
| Feng, J., Gerrans, P., & Clark, G. (2014). *Understanding superannuation contribution decisions: Theory and evidence* (CSIRO-Monash Superannuation Research Cluster Working Paper). Retrieved from <http://www.superresearchcluster.com/media/documents/outcomes-to-date/cp3wp1-understanding-superannuation-contribution-decisions-theory-and-evidence-1.pdf> | Did not meet design criteria |
| Federation of Credit Unions United to Serve the Underserved & Feline. (n.d.) Borrow and Save feasibility study report. Retrieved from <https://filene.org/assets/images-layout/BorrowandSave_FeasibiltyStudy_FINAL.pdf>  Fernández-Olit, B., Martín, J. M. M., & González, E. P. (2019). Systematized literature review on financial inclusion and exclusion in developed countries. *International Journal of Bank Marketing, 38*(3), 600-626.  Fernández-Olit, B., Paredes-Gázquez, J. D., & de la Cuesta-González, M. (2018). Are social and financial exclusion two sides of the same coin? An analysis of the financial integration of vulnerable people. *Social Indicators Research*, *135*(1), 245-268.  Fernández-Olit, B., Ruza, C., de la Cuesta-González, M., & Matilla-García, M. (2019). Banks and financial discrimination: What can be learnt from the Spanish Experience?. *Journal of Consumer Policy*, *42*(2), 303-323. | Did not meet design criteria  Did not meet design criteria  Did not meet design criteria  Did not meet design criteria |
| Finseraas, H. & Jakobsson, N. (2014). Does information about the pension system affect knowledge and retirement plans? Evidence from a survey experiment. *Journal of Pension Economics and Finance, 13*(3), 250-271. DOI:10.1017/S1474747213000310 | Did not meet design criteria |
| Fletcher, C. N., Beebout, G., & Mendenhall, S. (1997). Developing and evaluating personal finance education at the worksite: A case study. *Personal Finances and Worker Productivity*, *1*(1), 54-59.  Foltice, B., Arling, P. A., Kirby, J. E., & Saajasto, K. (2018). Persistent anchoring to default rates when electing 401 (k) contributions. *Review of Behavioral Finance*, *10*(1), 88-104.  Fraczek, B., & Matula, A. G. (2019). Financial education and the level of basic financial knowledge and its usage among people with Asperger syndrome. *Cypriot Journal of Educational Sciences*, *14*(4), 742-749. | Did not meet design criteria  Did not meet intervention criteria  Did not meet design criteria |
| Franco, A. (2012). *Who Needs and who Wants Financial Education? A Study of the Characteristics of Mexican Immigrants Participating in a Financial Education Program in New York City* (Doctoral dissertation, Columbia University). | Did not meet design criteria |
| Franz, C. (2016). Financial empowerment and health related quality of life in Family Scholar House participants. *Journal of Financial Therapy, 7*(1), 38-57. | Did not meet design criteria |
| Frey, J. J., Svoboda, D., Sander, R. L., Osteen, P. J., Callahan, C., & Elkinson, A. (2015). Evaluation of a continuing education training on client financial capability. *Journal of Social Work Education*, *51*(3), 439-456. | Did not meet design criteria |
| Frijns, B., Gilbert, A., & Tourani-Rad, A. (2014). Learning by doing: The role of financial experience in financial literacy. *Journal of Public Policy*, *34*(1), 123-154. | Did not meet intervention criteria |
| Fry, T., Mihajilo, S., Russell, R., & Brooks, R. (2006). The factors influencing saving in a Matched Savings Program: The case of the Australian Saver Plus Program. (May 2006),195-214. | Did not meet design criteria |
| Fry, T. R., Mihajilo, S., Russell, R., & Brooks, R. (2008). The factors influencing saving in a matched savings program: Goals, knowledge of payment instruments, and other behavior. *Journal of Family and Economic Issues*, *29*(2), 234-250.  Fürstenau, B., & Hommel, M. (2019). Developing financial competence about mortgage loans by informal learning using banks’ online calculators. *Empirical Research in Vocational Education and Training*, *11*(1), 10.  Galvez, M., Gilbert, B., Oneto, A., & DuBois, N. (2017). *Tacoma Housing Authority’s Children’s Savings Account Program Evaluation Interim Report*. Urban Institute. | Did not meet design criteria  Did not meet intervention criteria  Did not meet design criteria |
| Garbow, J., Jokela, R. H., Rudi, J., & Serido, J. (2019). Using American Indian legends to teach youths financial literacy: Innovative approaches to cultural adaptation. *Journal of Extension*, *57*(1), 1-5.  Garcia, Z. A., Francis, D., Christensen, A., MacArthur, S. S., Memmott, M., & Hill, P. A. (2017). The Money Mentors Program: Increasing financial literacy in Utah youths. *The Journal of Extension*, *55*(6), 1-4.  Garman, E. T., Kim, J., Kratzer, C. Y., Brunson, B. H., & Joo, S. (1999). Workplace financial education improves personal financial wellness. *Journal of Financial Counseling and Planning, 10,* 82-91. | Did not meet intervention criteria  Did not meet intervention criteria  Did not meet design criteria |
| Gartner, K. & Todd, R. M. (2005). *Effectiveness of online early intervention financial education programs for credit-card holders.* Proceedings 962, Federal Reserve Bank of Chicago.  Geyer, J., Freiman, L., Lubell, J., & Villarreal, M. (2019). Using the family self‐sufficiency program to help families with housing assistance improve earnings, credit score, and debt levels: A quasi‐experimental analysis. *Journal of Consumer Affairs*, *53*(3), 796-824.  Glidden, M. D., & Brown, T. C. (2017). Separated by bars or dollar signs? A comparative examination of the financial literacy of those incarcerated and the general population. *American Journal of Criminal Justice*, *42*(3), 533-553. | Did not meet design criteria  Did not meet design criteria  Did not meet intervention criteria |
| Gill, A., & Bhattacharya, R. (2017). The interaction of financial attitudes and financial knowledge: Evidence for low-income Hispanic families. *Atlantic Economic Journal*, *45*(4), 497-510.  Grimes, P. W., Rogers, K. E., & Smith, R. C. (2010). High school economic education and access to financial services. *Journal of Consumer Affairs*, *44*(2), 317-335. | Did not meet design criteria  Did not meet design criteria |
| Grinstead, M. L., Maulkin, T., Sabia, J.J., Koonce, J. & Palmer, L. (2011). Saving for success: Financial education and savings goal achievement in Individual Development Accounts. *Journal of Financial Counseling and Planning, 22(*2), 28-40. | Did not meet design criteria |
| Grinstein-Weiss, M., Chowa, G.A.N., & Casalotti, A. M. (2010). Individual Development Accounts for housing policy: Analysis of individual and program characteristics. *Housing Studies*, *25*(1), 63-82. | Did not meet design criteria |
| Grinstein‐Weiss, M., Curley, J., & Charles, P. (2007). Asset building in rural communities: The experience of Individual Development Accounts. *Rural Sociology*, *72*(1), 25-46. | Did not meet design criteria |
| Grinstein‐Weiss, M., Guo, S., Reinertson, V., & Russell, B. (2015). Financial education and savings outcomes for low‐income IDA Participants: Does Age Make a Difference?. *Journal of Consumer Affairs*, *49*(1), 156-185. | Did not meet design criteria |
| Grinstein-Weiss, M., Despard, M., Guo, S., Russell, B., Key, C. & Raghavan, R. (2016). Do tax-time savings deposits reduce hardship among low-income filers? A propensity score analysis. Journal of the Society for Social Work and Research, 7(4), 707-728.  Grinstein-Weiss, M., Irish, K., Parish, S. L., & Wagner, K. M. (2007). Using Individual Development Accounts to save for a home: Are there differences by race? *Social Service Review*, *81*(4), 657-681. | Did not meet outcomes criteria  Did not meet design  criteria |
| Grinstein-Weiss, M., Schreiner, M., Clancy, M., & Sherraden, M. (2001).  Family assets for independence in Minnesota (CSD Report 01-16).  St. Louis, MO: Washington University, Center for Social Development. | Did not meet design criteria |
| Grinstein-Weiss, M., Shanks, T. R. W., & Beverly, S. G. (2014). Family assets and child outcomes: Evidence and directions. *The Future of Children*, 24(1), 147-170. | Did not meet design criteria |
| Grinstein-Weiss, M. (2004). *IDAs for housing policy: Analysis of saving outcomes and racial differences*(Order No. 3147439). Retrieved from ProQuest Dissertations & Theses Global. (305107884). | Did not meet design criteria |
| Grinstein-Weiss, M., Kondratjeva, O., Roll, S. P., Pinto, O., & Gottlieb, D. (2019). The Saving for Every Child Program in Israel: An overview of a universal asset-building policy. *Asia Pacific Journal of Social Work and Development*, *29*(1), 20-33.  Grinstein-Weiss, M., Pinto, O., Kondratjeva, O., Roll, S. P., Bufe, S., Barkali, N., & Gottlieb, D. (2019). Enrollment and participation in a universal child savings program: Evidence from the rollout of Israel's National Program. *Children and Youth Services Review*, *101*, 225-238.  Grinstein-Weiss, M., Wagner, K., & Ssewamala, F. M. (2006). Saving and asset accumulation among low-income families with children in IDAs. *Children and Youth Services Review*, *28*(2), 193-211. | Did not meet design criteria  Did not meet design criteria  Did not meet design criteria |
| Grinstein-Weiss, M., Yeo, Y. H., Despard, M. R., Casalotti, A. M., & Zhan, M. (2010). Does prior banking experience matter? Differences of the banked and unbanked in individual development accounts. *Journal of Family and Economic Issues*, *31*(2), 212-227. | Did not meet design criteria |
| Grinstein‐Weiss, M., Zhan, M., & Sherraden, M. (2006). Saving performance in Individual Development Accounts: Does marital status matter? *Journal of Marriage and Family*, *68*(1), 192-204. | Did not meet design criteria |
| Guo, L., Arnould, E. J., Gruen, T. W., & Tang, C. (2013). Socializing to co-produce: Pathways to consumers’ financial well-being. *Journal of Service Research*, *16*(4), 549-563.  Gyurovski, I. I. (2017). *Predictors and implications of personal finance management* (No. THESIS). University of Chicago. | Did not meet design criteria  Did not meet intervention criteria |
| Habila, M. (2015). *Influence of Financial Education on Retirement Security: Evidence from the state of Illinois* (MPRA working paper no. 73988). <https://mpra.ub.uni-muenchen.de/73988/> | Did not meet design criteria |
| Haliassos, M., Jansson, T., & Karabulut, Y. (2020). Financial literacy externalities. *The Review of Financial Studies*, *33*(2), 950-989.  Han, C. (2007). *Savings in Individual Development Accounts: Multilevel analyses of institutions*(Order No. 3299956). Retrieved from ProQuest Dissertations & Theses Global. (304801702). | Did not meet intervention  Did not meet design  criteria |
| Han, C. (2009). Unemployment, financial hardship, and savings in Individual Development Accounts. *Journal of Poverty*, *13*(1), 74-95.  Harper, A., Baker, M., Edwards, D., Herring, Y., & Staeheli, M. (2018). Disabled, poor, and poorly served: access to and use of financial services by people with serious mental illness. *Social Service Review*, *92*(2), 202-240.  Haroon, H., Derbigny, D., Wadbia, O. &Wiedrich, K. (2019). *From paychecks to prosperity: Building the financial capability of youth in workforce programs.* Prosperity Now and Citi Foundation. | Did not meet design criteria  Did not meet design  criteria  Did not meet design  criteria |
| Hartarska, V. & Gonzalez-Vega, C. (2005). Credit counseling and mortgage termination by low-income households. *Journal of Real Estate Finance and Economics 30*(3), 227-243. DOI:10.1007/s11146-005-6405-z | Did not meet design criteria |
| Hartarska, V. & Gonzalez-Vega, C . (2006). Evidence on the effect of credit counseling on mortgage loan default by low-income households. *Journal of Housing Economics, 15*(1), 63-79. DOI:10.1016/j.jhe.2006.02.002  Hastings, J., & Mitchell, O. S. (2020). How financial literacy and impatience shape retirement wealth and investment behaviors. *Journal of Pension Economics & Finance*, *19*(1), 1-20. | Did not meet design criteria  Did not meet intervention criteria |
| Haupt, M. (2012). Pension information, financial literacy, and retirement savings behavior in Germany (first draft). Retrieved from <http://fileserver.carloalberto.org/cerp/2012cerpconferencepapers/Haupt_CeRP.pdf> | Did not meet design criteria |
| Haynes-Bordas, R., Kiss, D. E., & Yilmazer, T. (2008). Effectiveness of financial education on financial management behavior and account usage: Evidence from a ‘second chance’ program. *Journal of Family and Economic Issues*, *29*(3), 362. | Did not meet design criteria |
| Heckman, S. J. (2012). *A comparison of two savings measures: An application of Institutional Theory among low-income households* (Thesis). Ohio State University. | Did not meet design criteria |
| Hein, M., Losby, J., & Shir, A. (2006). The Office of Refugee Resettlement’s Individual Development Account (IDA) Program: An Evaluation Report. *Institute for Social and Economic Development, Washington, DC*. | Did not meet design criteria |
| Helman, R., Copeland, C., VanDerhei, J. (2006). *Will more of us be working forever? The 2006 Retirement Confidence Survey* (EBRI issue brief No. 292). | Did not meet design criteria |
| Helman, R. & Paladino, V. (2004). *Will Americans Ever Become Savers? The 14th Retirement Confidence Survey* (EBRI Issue Brief No. 268). Retrieved from <https://www.ebri.org/crawler/view/will-americans-ever-become-savers-the-14th-retirement-confidence-survey-2004-496>  Herbaut, E., & Geven, K. M. (2019). *What works to reduce inequalities in Higher Education? A systematic review of the (quasi-) experimental literature on outreach and financial aid*. The World Bank. | Did not meet design criteria  Did not meet design criteria |
| Herispon, H. (2018). The effect of bank behavior, financial literacy on financial inclusion and debt behavior in household consumption. In *International Conference on Social Sciences, Humanities, Economics and Law*. European Alliance for Innovation (EAI).  Hershey, D. A., Mowen, J. C., & Jacobs-Lawson, J. M. (2003). An experimental comparison of retirement planning intervention seminars. *Educational Gerontology*, *29*(4), 339-359. | Did not meet design criteria  Did not meet intervention criteria |
| Hetling, A., Postmus, J. L., & Kaltz, C. (2016). A randomized controlled trial of a financial literacy curriculum for survivors of intimate partner violence. *Journal of Family and Economic Issues*, *37*(4), 672-685. | Did not meet design criteria |
| Hirad, A., Zorn, P. M., Retsinas, N., & Belsky, E. (2002). A little knowledge is a good thing: Empirical evidence of the effectiveness of pre-purchase homeownership counseling. Washington, DC: The Brookings Institution Press. | Did not meet design criteria |
| Holguin, I. (2015). Start2Save: Helping working families meet unexpected expenses and opportunities. In J. M. Collins (Ed.), *A Fragile Balance* (pp. 175-191). New York: Palgrave Macmillan. | Did not meet design criteria |
| Huang, J., Beverly, S., Clancy, M., Lassar, T., & Sherraden, M. (2013). Early program enrollment in a statewide Child Development Account Program. *Journal of Policy Practice*, *12*(1), 62-81.  Huang, J., Beverly, S. G., Kim, Y., Clancy, M. M., & Sherraden, M. (2019). Exploring a model for integrating Child Development Accounts with social services for vulnerable families. *Journal of Consumer Affairs*, *53*(3), 770-795. | Did not meet design criteria  Did not meet outcomes criteria |
| Huang, J., Kim, Y., & Sherraden, M. (2017). Material hardship and children's social‐emotional development: Testing mitigating effects of Child Development Accounts in a randomized experiment. *Child: Care, Health and Development*, *43*(1), 89-96. | Did not meet outcomes criteria |
| Huang, J., Kim, Y., Sherraden, M., & Clancy, M. (2017). Unmarried mothers and children’s social-emotional development: The role of Child Development Accounts. *Journal of Child and Family Studies*, *26*(1), 234-247. | Did not meet outcomes criteria |
| Huang, J., Nam, Y., & Lee, E. J. (2015). Financial capability and economic hardship among low-income older Asian immigrants in a supported employment program. *Journal of Family and Economic Issues*, *36*(2), 239-250. | Did not meet intervention criteria |
| Huang, J., Nam, Y., Sherraden, M., & Clancy, M. M. (2016). Improved financial capability can reduce material hardship among mothers. *Social Work*, *61*(4), 313-320. | Did not meet design criteria |
| Huang, J., Sherraden, M., & Purnell, J. Q. (2014). Impacts of Child Development Accounts on maternal depressive symptoms: Evidence from a randomized statewide policy experiment. *Social Science & Medicine*, *112*, 30-38. | Did not meet outcomes criteria |
| Hudson, C. R., & Palmer, L. (2014). Low-income employees: The relationship between information from formal advisors and financial behaviors. *Financial Services Review*, *23*(1), 25-44.  Iterbeke, K., De Witte, K., Declercq, K., & Schelfhout, W. (2019). The effect of ability matching and differentiated instruction in financial literacy education. Evidence from two randomised control trials. *Economics of Education Review*, 78. <https://doi.org/10.1016/j.econedurev.2019.101949> | Did not meet design criteria  Did not meet intervention criteria |
| Jamison, J. C., Karlan, D., & Zinman, J. (2014). Financial education and access to savings accounts: Complements or substitutes? Evidence from Ugandan youth clubs (Working Paper 20135). National Bureau of Economic Research.  Johnson, C. L., Bartholomae, S., Serido, J., Katras, M. J., & Tobe, E. (2020). Launching a text message intervention to assist student loan borrowers with repayment decisions. *Family and Consumer Sciences Research Journal*, *48*(3), 230-244.  Johnson, L. (2018). *Increasing financial empowerment for survivors of intimate partner violence: a longitudinal evaluation of a financial knowledge curriculum* (Doctoral dissertation, Rutgers University-School of Graduate Studies).  Johnson, L., Lee, Y., Njenga, G., Kieyah, J., Osei-Akoto, I., Orgales, C. R., ... & Sherraden, M. (2018). School banking as a strategy for strengthening youth economic participation in developing countries: Lessons from YouthSave. *Global Social Welfare*, *5*(4), 265-275. | Did not meet OECD criteria  Did not meet intervention criteria  Did not meet intervention criteria  Did not meet OECD criteria |
| Johnson, S., Miller, M., Qiu, J., Spenser, J., & Stout, O. (2011). Effects of homeownership education on foreclosure prevention for first-time homebuyers. Master of Public Administration Capstone Applied Research Project; University of Oregon Department of Planning, Public Policy, and Management. | Did not meet design criteria |
| Johnson, E., & Sherraden, M. S. (2007). From financial literacy to financial capability among youth. *Journal of Sociology & Social Welfare*, *34(3)*, 119-145. | Did not meet design criteria |
| Jones, L. E. (2009) A behavioral approach to saving: Evidence from a randomized field experiment. Doctoral Dissertation, Ohio State University, Columbus, OH. | Did not meet design criteria |
| Jones, M. A. (2002). *Empowered by choices of entrepreneurship: An intervention for female african american high school students through the My Entrepreneurial Journey (MEJ) program*(Order No. 3052988). Retrieved from ProQuest Dissertations & Theses Global. (305497241).  Jones, W. D. (2019). *The relationship between personal & family characteristics, Promoting readiness in minors receiving Supplemental Security (PROMISE) interventions, and Individual Development Account (IDA) saving participation among transition-age youth with disabilities*. The University of Wisconsin-Madison.  Kalmi, P. (2018). The Effects of Financial Education: Evidence from Finnish Lower Secondary Schools. *Economic Notes: Review of Banking, Finance and Monetary Economics*, *47*(2-3), 353-386.  Kalwij, A., Alessie, R., Dinkova, M., Schonewille, G., Van der Schors, A., & Van der Werf, M. (2019). The effects of financial education on financial literacy and savings behavior: Evidence from a controlled field experiment in Dutch primary schools. *Journal of Consumer Affairs*, *53*(3), 699-730. | Did not meet design criteria  Did not meet outcomes criteria  Did not meet intervention criteria  Did not meet intervention criteria |
| Karlan, D., Shafir, E., & Zinman, J. (2012). Super savers? A randomized evaluation of commitment savings and financial counseling in New York City (Working Paper). Innovation Poverty Action. | Did not meet design criteria |
| Kempson, E., McKay, S. & Collard, S. (2005). Incentives to save: Encouraging saving among low-income households (Final report on the Saving Gateway pilot project). Bristol, UK: University of Bristol, Personal Finance Research Centre. | Did not meet intervention criteria |
| Key, C., Tucker, J. N., Grinstein-Weiss, M. & Comer, I. (2015). Tax-time savings among low-income households in the $aveNYC program. Journal of Consumer Affairs, 49(3), 489-518. | Did not meet intervention criteria |
| Kim, J. (2004). Impact of a workplace financial education program on financial attitude, financial behavior, financial well-being, and financial knowledge. In *Proceedings of the Association for Financial Counseling and Planning Education*. Retrieved from <https://pfeef.org/wp-content/uploads/2016/09/Kim-Impacts-of-WFE-AFCPE-04.pdf> | Did not meet design criteria |
| Kim, J., Bagwell, D. C., & Garman, E. T. (1998). Evaluation of workplace personal financial education. *Personal Finances and Worker Productivity*, *2*(1), 187-192. | Did not meet design criteria |
| Kim, J., & Garman, E. T. (2003). Financial education and advice changes worker attitudes and behaviors. *Journal of Compensation and Benefits*, *19*(5), 7-13. | Did not meet design criteria |
| Kim, J., Garman, E. T., & Quach, A. (2005). Workplace financial education participation and retirement savings by employees and their spouses. *Journal of Personal Finance 4*(3), 92-108.  Kim, Y., Huang, J., Sherraden, M., & Clancy, M. (2017). Child development accounts, parental savings, and parental educational expectations: A path model. *Children and Youth Services Review*, *79*, 20-28. | Did not meet design criteria  Did not meet outcomes criteria |
| Kim, J., Kwon, J., & Anderson, E. A. (2005). Factors related to retirement confidence: Retirement preparation and workplace financial education. *Financial Counseling and Planning*, *16*(2), 77-89. | Did not meet design criteria |
| Kim, J. S., & Johnson, T. K. (2012). The academic and behavioral effects of a child savings account program on at-risk high school students. *School Social Work Journal*, *37*(1), 75-95. | Did not meet design criteria |
| Kim, Y., Sherraden, M., Huang, J., & Clancy, M. (2015). Child Development Accounts and parental educational expectations for young children: Early evidence from a statewide social experiment. *Social Service Review*, *89*(1), 99-137.  Knoll, M. A., Cooper, C. R., Johnson, H., Sieminski, D., & Banker, M. (2019). Planning for tax-time savings. *Consumer Financial Protection Bureau Data Point Series*, (19-2).  Koomson, I., Villano, R. A., & Hadley, D. (2020). Intensifying financial inclusion through the provision of financial literacy training: A gendered perspective. *Applied Economics*, *52*(4), 375-387.  Krische, S. D. (2019). Investment experience, financial literacy, and investment‐related judgments. *Contemporary Accounting Research*, *36*(3), 1634-1668. | Did not meet outcomes criteria  Did not meet intervention criteriantervention  Did not meet intervention criteria  Did not meet design criteria |
| Klawitter, M., Anderson, C L., & Gugerty, M. K. (2012). Savings and personal discount rates in a Matched Savings Program for Low Income Families. *Contemporary Economic Policy*, *31*(3), 468–485. | Did not meet design criteria |
| Klawitter, M., Stromski, L., & Calleja, J. (2005). United Way of King County Collaborative: Individual Development Account (Progress Report). Seattle, WA: United Way of King County, Individual Development Account Collaborative.  Kondratjeva, O., Roll, S., Bufe, S., & Grinstein-Weiss, M. (2019). Providing financial tips to low-and moderate-income tax filers: Awareness, usage, and usefulness. *Consumer Interests Annual*, *65,* 1-6. | Did not meet design criteria  Did not meet outcome criteria |
| Krajnak, P. A., Burns, S. A., & Natchek, S. M. (2008). Retirement education in the workplace. *Financial Services Review*, *17*(2), 131-141.  Lahav, E., Shavit, T., & Benzion, U. (2018). Don’t let them fool you: Adolescents’ present-orientation and inferior financial understanding. *Young*, *26*(3), 271-289.  L’Esperance, M. (2019). *Essays in household finance: The role of non-traditional approaches to building financial capability* (Doctoral dissertation, University of Wisconsin--Madison).  Lee, N., Beeler Stücklin, S., Lopez Rodriguez, P., Faris, M., & Mukaka, I. (2019). Financial education for HIV-vulnerable youth, orphans and vulnerable children: a systematic review of outcome evidence. *Vulnerable Children and Youth Studies*, *14*(3), 191-218. | Did not meet design criteria  Did not meet design criteria  Did not meet design criteria  Did not meet design criteria |
| Lerman, R. L. & Bell, E. (2006). *Financial literacy strategies: Where do we go from here?* (Policy brief 2006-PB-10). Terre Haute, Indiana: Indiana State University, Networks Financial Institute. | Did not meet design criteria |
| Lewis, M., O’Brien, M., & Elliott, W. (2017). Immigrant Latino families saving against great odds: The case of CSAs and the Prosperity Kids Program. *Race and Social Problems*, *9*(3), 192-206. | Did not meet design criteria |
| Li, W., Bai, B., Goodman, L., & Zhu, J. (2016). NeighborWorks America’s homeownership education and counseling: Who receives it and is it effective? The Urban Institute. | Did not meet design criteria |
| Linnenbrink, M. L. (2006). Factors associated with savings and the achievement of savings goals in Individual Development Accounts: Evidence from the American dream demonstration (Theses). University of Georgia. | Did not meet design criteria |
| Loibl, C. & Bird, B. R. (2009). Survey of former IDA program participants: How do they fare? *Journal of Extension, 47*(6), 1-12. | Did not meet design criteria |
| Loibl, C., Bird, B. R., Grinstein-Weiss, M., & Zhan, M. (2009). Yes, the poor can be taught to save - Evidence from a survey of IDA Program participants. *Advances in Consumer Research, 36,* 868. | Did not meet design criteria |
| Loibl, C., Grinstein-Weiss, M., Zhan, M., & Bird, B. R. (2010). More than a penny saved: Long-term changes in behavior among savings program participants. *Journal of Consumer Affairs, 44*(1), 98-126. DOI:10.1111/j.1745-6606.2010.01159.x | Did not meet design criteria |
| Loibl, C., & Hira, T. K. (2005). Self-directed financial learning and financial satisfaction. *Journal of Financial Counseling and Planning*, *16*(1), 11-21. | Did not meet design criteria |
| Loibl, C., Jones, L., & Loewenstein, G. (2018). Testing strategies to increase saving and retention in IDA programs. Working paper. | Did not meet design criteria |
| Loke, V., & Choi, L. (2015). Increasing youth financial capability: A subsample analysis of Asian American and Pacific Islander participants in the MyPath Savings Initiative. *AAPI Nexus: Policy, Practice and Community*, *13*(1), 45-71. | Did not meet design criteria |
| Loke, V., Choi, L., & Libby, M. (2015). Increasing youth financial capability: An evaluation of the MyPath savings initiative. *Journal of Consumer Affairs*, *49*(1), 97-126. | Did not meet design criteria |
| Loke, V., Clancy, M., & Zager, R. (2009). *Account monitoring research at Michigan SEED* (CSD Research Report No. 09-62). St. Louis: Washington University Center for Social Development. | Did not meet design criteria |
| Lombe, M., Huang, J., Putnam, M., & Cooney, K. (2010). Exploring saving performance in an IDA program: Findings for people with disabilities. *Social Work Research*, *34*(2), 83-93. | Did not meet design criteria |
| Lombe, M., Inoue, M., Mahoney, K., Chu, Y., & Putnam, M. (2016). Understanding effects of flexible spending accounts on people with disabilities: The case of a consumer-directed care program. *Journal of social work in disability & rehabilitation*, *15*(1), 62-75. | Did not meet design criteria |
| Lombe, M., Nebbitt, V., & Buerlein, J. (2007). Perceived effects of participation in an asset-building program on construction of future possibilities. *Families in Society: The Journal of Contemporary Social Services*, *88*(3), 463-471. | Did not meet design criteria |
| Lombe, M., Putnam, M., & Huang, J. (2008). Exploring effects of institutional characteristics on saving outcome: The case of the Cash and Counseling program. *Journal of Policy Practice*, *7*(4), 260-279. | Did not meet design criteria |
| Losby, J. L. (2003). *Michigan IDA partnership: Year 2 program evaluation report (executive summary).* Newark, DE: Institute for Social and Economic Development. | Did not meet design criteria |
| Lusardi, A. (2003). *The impact of financial education on savings and asset allocation* (working paper 2003-61). University of Michigan Retirement Research Center. | Did not meet design criteria |
| Lusardi, A. (2004). Saving and the effectiveness of financial education. Working Paper. Dartmouth College. | Did not meet design criteria |
| Lyons, A.C., & Scherpf, E. (2004). Moving from unbanked to banked: Evidence from the Money Smart program. *Financial Services Review, 13*(3), 215-231.  Lyskawa, J. A. (2011). *Social and financial supports: The effect on saving in Individual Development Accounts*(Order No. 1491477). Retrieved from ProQuest Dissertations & Theses Global. (865286916). | Did not meet design criteria  Did not meet design criteria |
| Mandell, L. (2009). *Two cheers for school-based financial education (Issue brief).* The Aspen Institute, Initiative on Financial Security. Retrieved from <https://www.aspeninstitute.org/publications/two-cheer-school-based-financial-education/> | Did not meet design criteria |
| Manturuk, K., Dorrance, J., & Riley, S. (2012). Factors affecting completion of a matched savings program: Impacts of time preference, discount rate, and financial hardship. *The Journal of Socio-Economics*, *41*(6), 836-842.  Martínez, C., & Puentes, E. (2018). Micro-entrepreneurship debt level and access to credit: Short-term impacts of a financial literacy program. *The European Journal of Development Research*, *30*(4), 613-629.  Martinez, R. A. Y. (2018). 5 ways your bank can improve family financial wellness. *Virginia Banking, 2*, 18-19. | Did not meet design criteria  Did not meet intervention criteria  Did not meet design criteria |
| Marzahl, D., Owen, O. S., Neumann, S., & Harriman, J. (2006). First accounts: a US Treasury Department program to expand access to financial institutions. *Profitwise News and Views*, 15-19. | Did not meet design criteria |
| Mason, L. R., Nam, Y., Clancy, M., Kim, Y., & Loke, V. (2010). Child Development Accounts and saving for children's future: Do financial incentives matter?. *Children and Youth Services Review*, *32*(11), 1570-1576. | Did not meet design criteria |
| Maxwell, D., & Paxton, W. (2005). [ADD: Lessons from the UK](https://csd.wustl.edu/Publications/Documents/RP05-39.pdf)(CSD Research Report 05-39). St. Louis, MO: Washington University, Center for Social Development. | Did not meet design criteria |
| Mayer, N. S. & Temkin, K. (2013). Prepurchase counseling effects on mortgage performance: Empirical analysis of NeighborWorks® America's experience. Neil Mayer & Associates, Albany, CA. | Did not meet design criteria |
| Mayer, N. S. & Temkin, K. (2016). Prepurchase counseling effects on mortgage performance: Empirical analysis of NeighborWorks® America's experience. *Cityscape: A Journal of Policy Development and Reseach, 18*(2), 73-98. | Did not meet design criteria |
| McBride, A. M. (2003). *Asset ownership among low-income and low wealth individuals: opportunity asset ownership, and civic engagement.* Retrieved from Dissertation Abstracts International. (UMI No. 3105970) | Did not meet outcomes criteria |
| McBride, A. M., Lombe, M., & Beverly, S. G. (2003). *The effects of Individual Development Account programs: Perceptions of participants*(CSD Working Paper No. 03-06). St. Louis, MO: Washington University, Center for Social Development. | Did not meet design criteria |
| McKernan, S. M., Rademacher, I., Ratcliffe, C., Wiedrich, K., & Gallagher, M. (2011). Weathering the storm: How have IDA homebuyers fared in the foreclosure crisis? *Housing Policy Debate, 21*(4), 605-625. DOI:10.1080/10511482.2011.600698 | Did not meet design criteria |
| McKernan, S. M., Ratcliffe, C. & Nam, Y. (2007). *The effects of welfare and IDA program rules on the asset holdings of low-income families, A report in the series Poor Finances: Assets and Low-Income Households.* Retrieved from <https://www.urban.org/research/publication/effects-welfare-and-ida-program-rules-asset-holdings-low-income-families> | Did not meet design criteria |
| McMath, V. M. Financial pre-purchase homebuyer education: The pre- and post impact, behaviors, and perceptions of course completers. Doctoral Dissertation, Trevecca Nazarene University, Nashville TN. | Did not meet design criteria |
| Mende, M., & Van Doorn, J. (2015). Coproduction of transformative services as a pathway to improved consumer well-being: Findings from a longitudinal study on financial counseling. *Journal of Service Research*, *18*(3), 351-368.  Mielitz, K. K. S., MacDonald, M., & Lurtz, M. (2018). Financial literacy education in a work release program for an incarcerated sample. *Journal of Financial Counseling and Planning*, *29*(2), 316-327. | Did not meet design criteria  Did not meet intervention criteria |
| Migheli, M., & Moscarola, F. C. (2017). Gender differences in financial education: Evidence from primary school. *De Economist*, *165*(3), 321-347.  Moore, A., Beverly, S., Schreiner, M., Sherraden, M., Lombe, M., Cho, E. N. Y., et al. (2001). *Saving, IDA programs, and effects of IDAs: A survey of participants* (CSD Report 01-24). St. Louis, MO: Washington University, Center for Social Development. | Did not meet design criteria  Did not meet design criteria |
| Moulton, S., Loibl, C., Collins, J. M., & Savikhin, A. (2011). *Field Experiments on the Impacts of Financial Planning Interventions for Recent Homebuyers* (Working Paper 2011-CFS.5). Madison, WI: University of Wisconsin-Madison, Center for Financial Security.  Moulton, S. R., Peck, L. R., Fiore, N., Gruenstein Bocian, D., & DeMarco, D. (2019). An examination of participation in Homebuyer Education and Counseling services. *Journal of Consumer Affairs*, *53*(3), 825-847. | Did not meet design criteria  Did not meet design criteria |
| Muller, L. A. (2001). Does retirement education teach people to save pension distributions. *Social Security Bulletin*, *64*, 48. | Did not meet design criteria |
| Muller, L. A. (2003). Investment choice in defined contribution plans: The effects of retirement education on asset allocation. *Benefits Quarterly*, *19*(2), 76. | Did not meet design criteria |
| Murphy-Erby, Y., Jordan, S., Shobe, M., & Christy-McMullin, K. (2009, March). Individual Development Accounts and social justice. In *Forum on Public Policy, 1,* 1-21. | Did not meet design criteria |
| Nam, Y., Hole, E., Sherraden, M., & Clancy, M. (2014). *Program knowledge and savings outcomes in a Child Development Account experiment* (CSD Working Paper No. 14-22). St. Louis, MO: Washington University, Center for Social Development. | Did not meet design criteria |
| Nam, Y., Wikoff, N., & Sherraden, M. (2016). Economic intervention and parenting: A randomized experiment of statewide Child Development Accounts. *Research on Social Work Practice*, *26*(4), 339-349.  Nam, Y., Hole, E., Sherraden, M., & Clancy, M. M. (2018). Program Knowledge and Racial Disparities in Savings Outcomes in a Child Development Account Experiment. *Journal of Family and Economic Issues*, *39*(1), 145-162. | Did not meet outcomes criteria  Did not meet design criteria |
| Nguyen, K. N. (2013). *High school seniors' financial knowledge: The impact of financial literacy classes and developmental assets*(Order No. 1542137). Retrieved from ProQuest Dissertations & Theses Global. (1425304607).  Nguyen, T. T. T. (2017). *Exploring the impacts of financial education on behaviour change in personal finance management: Evidence from the PUFin Educational Programme “Managing My Money”* (Doctoral dissertation, The Open University). | Did not meet outcomes criteria  Did not meet intervention criteria |
| Ntalianis, M. & Wise, V. (2011). The role of financial education in retirement planning. *Australasian Accounting, Business and Finance Journal,* *5*(2), 23-37. Retrieved from http://ro.uow.edu.au/aabfj/vol5/iss2/3 | Did not meet design criteria |
| Nyce, S. A. (2005). *The importance of financial communication for participation rates and contribution levels in 401 (k) plans* (PRC working paper No. 2005-3). Philadelphia, PA: University of Pennsylvania, Wharton School, Pension Research Council. | Did not meet design criteria |
| O’Neill, B. (2006). IDA financial education: Quantitative and qualitative impacts. *Journal of Financial Counseling and Planning Volume*, *16*(1), 73-87. | Did not meet design criteria |
| O'Neill, B., Sorhaindo, B., Xiao, J. J., & Garman, E. T. (2005). Financially distressed consumers: Their financial practices, financial well-being, and health. *Journal of Financial Counseling and Planning, 16*(1), 73-87. | Did not meet design criteria |
| Okech, D. (2008). *Individual characteristics and social services associated with asset building in a children's college savings account program for low-income families*(Order No. 3336867). Retrieved from ProQuest Dissertations & Theses Global. (304148493). | Did not meet design criteria |
| Okech, D. (2013). The independent effects of socio-demographic and programmatic factors on economic strain among parents in a Child Savings Accounts program. *Children and Youth Services Review*, *35*(6), 950-959. | Did not meet outcomes criteria |
| Okech, D., Little, T. D., & Williams-Shanks, T. (2011). Early savings for children's higher education: A comparison between savers and non-savers in a Child Development Account program. *Children and Youth Services Review, 33*(2011), 1592-1598. doi:10.1016/j.childyouth.2011.03.025 | Did not meet design criteria |
| Olsen, A., & Whitman, K. (2007). Effective retirement savings programs: Design features and financial education. *Social Security Bulletin*, *67*, 53.  Ottaviani, C., & Vandone, D. (2018). Financial literacy, debt burden and impulsivity: A mediation analysis. *Economic Notes: Review of Banking, Finance and Monetary Economics*, *47*(2-3), 439-454.  Otto, N. (2018). Financial wellness: Financial wellbeing programs on the rise. *Employee Benefit News, 32*(3), 1. | Did not meet design criteria  Did not meet intervention criteria  Did not meet design criteria |
| Ouverson, K. & Johnson, P. (2013). Family assets for independence in Minnesota (FAIM). Research report. West Central Minnesota Community Action, Inc. Retrieved from [www.minnesotafaim.org](http://www.minnesotafaim.org) | Did not meet design criteria |
| Palmer, L., Pichot, T., & Kunovskaya, I. (2016). Promoting savings at tax time through a video-based solution-focused brief coaching intervention. *Journal of Financial Therapy*, *7*(1), 2. | Did not meet intervention criteria |
| Parker, J. (2013). Developing financial capability through IDA saving clubs. In J. Birkenmaier, J., M. Sherraden, & J. Curley (Eds.), *Financial Education and Capability: Research, Education, Policy, and Practice*, (pp. 174-191). | Did not meet design criteria |
| Peeters, N., Rijk, K., Soetens, B., Storms, B., & Hermans, K. (2018). A systematic literature review to identify successful elements for financial education and counseling in groups. *Journal of Consumer Affairs*, *52*(2), 415-440.  Phillips, L., & Chavarin, M. (2014). *Connecting domestic violence survivors to one-on-one financial counseling services: Lessons learned.* San Francisco, CA: San Francisco Office of Financial Empowerment. | Did not meet design criteria  Did not meet design criteria |
| Potrich, A. C. G., Vieira, K. M., & Mendes-Da-Silva, W. (2016). Development of a financial literacy model for university students. *Management Research Review*, *39*(3), 356-376. | Did not meet design criteria |
| Prawitz, A., & Cohart, J. (2014). Workplace financial education facilitates improvement in personal financial behaviors. *Journal of Financial Counseling and Planning, 25*(1), 5-26. | Did not meet intervention criteria |
| Quercia, R., & Spader, J. (2008). Does homeownership counseling affect the prepayment and default behavior of affordable mortgage borrowers? *Journal of Policy Analysis and Management*, *27*(2), 304-325. | Did not meet design criteria |
| Quinn, J. M. (2000). Mainstreaming financial education as an employee benefit. *Journal of Financial Planning*, *13*(5), 70. | Did not meet design criteria |
| Quittman, L. (2010). Community Financial Access Pilot: Creating templates for expanding financial opportunities. *Community Investment, 22*(2), 34-45.  Rashid, A. A. A., Jantan, M. S., Fairuz, M. A. F. A., & Halim, N. A. A. (2020). Financial Knowledge and Personal Financial Literacy in Investment Priorities Among University Students. *Available at SSRN 3541998*. | Did not meet design criteria  Did not meet intervention criteria |
| Rauscher, E., Elliott, W., O'Brien, M., Callahan, J., & Steensma, J. (2017). Examining the relationship between parental educational expectations and a community-based children's savings account program. *Children and Youth Services Review, 74*(2017), 96-107. | Did not meet outcomes criteria |
| Reutebuch, T. G. (1999). *Asset accumulation and the economic and social development of working poor households and communities*(Order No. 9941417). Retrieved from ProQuest Dissertations & Theses Global. (304540045). | Did not meet design criteria |
| Riitsalu, L. (2018). Goals, commitment and peer effects as tools for improving the behavioural outcomes of financial education. *Citizenship, Social and Economics Education*, *17*(3), 188-209.  Robertson, A. S., & Curley, J. (2016). *Annual report on the ASSET Project’s Head Start Family Financial Capability Pilot: 2014–2015* (CSD Research Report No. 16-04). St. Louis, MO: Washington University, Center for Social Development. | Did not meet intervention criteria  Did not meet design criteria |
| Rohe, W. M., Gorham, L. S., & Quercia, R. G. (2005). Individual development accounts: Participants’ characteristics and success. *Journal of Urban Affairs*, *27*(5), 503-520. | Did not meet outcomes criteria |
| Roll, S. & Moulton, S. (2016). The impact of credit counseling on consumer outcomes: Evidence from a national demonstration program. Washington, D.C.: Federal Deposit Insurance Corporation. | Did not meet design criteria |
| Roll, S. P. (2016) Credit counseling, financial coaching, and client outcomes: An examination of program impacts and implementation dynamics. Doctoral Dissertation. Columbus, OH: The Ohio State University. | Did not meet design criteria |
| Rosato, N. S. (2005). An Evaluation of the Success of Saving-growing Personal Assets Project: Individual Development Accounts for People with Developmental Disabilities. New Brunswick, NJ: Rutgers Center for State Health Policy. | Did not meet design criteria |
| Rothwell, D. W. (2010). Asset Building Among Native Hawaiians: Lessons From the Kahikü Individual Development Account Program. *Hülili: Multidisciplinary Research on Hawaiian Well-Being , 6*(2010), 187-211. | Did not meet design criteria |
| Rothwell, D. W. (2013). Pathways to higher education for Native Hawaiian Individual Development Account participants. *The International Indigenous Policy Journal*, *4*(4), 1-18. | Did not meet design criteria |
| Rothwell, D. W. & Sultana, N. (2013). Cash-flow and savings practices of low-income households: Evidence from a follow-up study of IDA participants. *Journal of Social Service Research, 39(*2), 281-292. DOI:10.1080/01488376.2012.754828  Sari, R. C., & Fatimah, P. R. (2017). Bringing voluntary financial education in emerging economy: Role of financial socialization during elementary years. *The Asia-Pacific Education Researcher*, *26*(3-4), 183-192.  Shi, X., Prevett, P., Farnsworth, V., Kwong, K. C., Wan, W., He, F., ... & Zhen, L. (2019). Modeling changes to survey response items over time in a Britain financial literacy education study. *Journal of Financial Counseling and Planning*, *30*(1), 56-66. | Did not meet design criteria  Did not meet intervention criteria  Did not meet intervention criteria |
| Southern Good Faith Fund. (2006). Arkansas Individual Development Account (IDA) program: Survey shows broad impact. *Policy Points*, *28*, 1-5.  Stacy, C. P., Theodos, B., & Bai, B. (2018). How to prevent mortgage default without skin in the game: Evidence from an integrated homeownership support nonprofit. *Journal of Housing Economics*, *39*, 17-24.  Roll, S. (2016). *Credit counseling, financial coaching, and client outcomes: An examination of program impacts and implementation dynamics* (Doctoral dissertation, The Ohio State University). | Did not meet design criteria  Did not meet design criteria  Did not meet design criteria |
| Russell, R., Brooks, R. D., & Nair, A. (2006). Evaluating a financial literacy program: The case of the Australian MoneyMinded program. In M. Jackson, S. Singh, & R. Russell (Eds.), *Proceedings of the Financial Literacy, Banking & Identity Conference*(pp. 1 - 11). | Did not meet design criteria |
| Russell, R., Brooks, R., Nair, A., & Fredline, L. (2006). The initial impacts of a matched savings program: The Saver Plus program. *Economic Papers*, *25*(1), 32-40. | Did not meet design criteria |
| Russell, R., & Fredline, L. (2004). Saver Plus Progress and Perspectives: Evaluation of the Savers Plus Pilot Project (Interim Report). Melbourne, AU: RMIT University, Research Development Unit. | Did not meet design criteria |
| Saliterman, V., & Sheckley, B. G. (2004). Adult learning principles and pension participant behavior. In Olivia S. Mitchell & Stephen P. Utkus (Eds.), *Pension design and structure: New lessons from behavioral finance* (pp. 222-234). Norfold: Oxford University Press. | Did not meet design criteria |
| Sanders, C. K. (2014). Savings for survivors: An individual development account program for survivors of intimate-partner violence. *Journal of Social Service Research*, *40*(3), 297-312.  Sari, R. C., & Fatimah, P. R. (2017). Bringing voluntary financial education in emerging economy: Role of financial socialization during elementary years. *The Asia-Pacific Education Researcher*, *26*(3-4), 183-192. | Did not meet design criteria  Did not meet intervention criteria |
| Schreiner, M., Clancy, M., & Sherraden, M. (2002). Saving performance in the American Dream Demonstration: A national demonstration of Individual Development Accounts (CSD Report 02-15).  St. Louis, MO: Washington University, Center for Social Development | Did not meet design criteria |
| Schreinder, M. & Sherraden, M. (2017). *Can the poor save?: Saving and asset building in individual development accounts*. New York: Routledge. | Did not meet design criteria |
| Schreiner, M., Sherraden, M., Clancy, M., Johnson, L., Curley, J., Grinstein-Weiss, M., et al. (2001). *Savings and asset accumulation in individual development accounts*(CSD Report 01-23). St. Louis, MO: Washington University, Center for Social Development. | Did not meet design criteria |
| Schreiner, M., Sherraden, M., Clancy, M., Johnson, L., Curley, J., Zhan, M., ... & Grinstein-Weiss, M. (2005). Assets and the poor: Evidence from individual development accounts. *Inclusion in the American dream: Assets, poverty, and public policy*, 185-215. | Did not meet design criteria |
| Seligman, J. S., & Bose, R. (2012). Learning by doing: Active employer sponsored retirement savings plan participation and household wealth accumulation. *The Quarterly Review of Economics and Finance*, *52*(2), 162-172. | Did not meet outcomes criteria |
| Servon, L. J., & Kaestner, R. (2008). Consumer financial literacy and the impact of online banking on the financial behavior of lower‐income bank customers. Journal of Consumer Affairs, 42(2), 271-305. | Did not meet intervention criteria |
| Shelton, G. G., & Hill, O. L. (1995). First-time homebuyers programs as an impetus for change in budget behavior. *Journal of Financial Counseling and Planning*, *6*, 83. | Did not meet design criteria |
| Sherraden, M. S., Johnson, L., Guo, B., & Elliott, W. (2011). Financial capability in children: Effects of participation in a school-based financial education and savings program. *Journal of Family and Economic Issues, 32*(3), 385-399. | Did not meet design criteria |
| Sherraden, M. S., Johnson, L., Elliott III, W., Porterfield, S., & Rainford, W. (2007). School-based children's saving accounts for college: The I Can Save program. *Children and Youth Services Review*, *29*(3), 294-312. | Did not meet design criteria |
| Sherraden, M., Schreiner, M., & Beverly, S. (2003). Income, institutions, and saving performance in individual development accounts. *Economic Development Quarterly*, *17*(1), 95-112. | Did not meet design criteria |
| Sherraden, M., & Stevens, J. (Eds.) (2010). [*Lessons from SEED: A national demonstration of Child Development Accounts*](https://csd.wustl.edu/Publications/Documents/RR10-35_SEEDSynthesis_Final.pdf). St. Louis, MO: Washington University, Center for Social Development.  Shim, S., Serido, J., & Lee, S. K. (2019). Problem‐solving orientations, financial self‐efficacy, and student‐loan repayment stress. *Journal of Consumer Affairs*, *53*(3), 1273-1296. | Did not meet design criteria  Did not meet design criteria |
| Shobe, M. A. (2001). *Relationships between assets and personal, social, and economic well-being*(Order No. 3018536). Retrieved from ProQuest Dissertations & Theses Global. (304697150). | Did not meet design criteria |
| Shockey, S. S. (2002). *Low -wealth adults' financial literacy, money management behaviors, and associated factors, including critical thinking*(Order No. 3039524). Retrieved from ProQuest Dissertations & Theses Global. (251720258). | Did not meet design criteria |
| Shtauber, A. A. (2013). *The Effects of Access to Mainstream Financial Services on the Poor: Evidence from Data on Recipients of Financial Education* (Columbia Business School Research Paper No. 14-11). Doi: <http://dx.doi.org./10.2139/ssrn.2403335> | Did not meet design criteria |
| Signal, L., Lanumata, T., & Bowers, S. (2012). Punching loan sharks on the nose: Effective interventions to reduce financial hardship in New Zealand. *Health Promotion Journal of Australia*, *23*(2), 108-111. | Did not meet design criteria |
| Skimmyhorn, W. (2012). Essays in behavioral household finance. Doctoral dissertation. Cambridge, MA: Harvard University. | Did not meet design criteria |
| Skimmyhorn, W. L., Davies, E. R., Mun, D., & Mitchell, B. (2016). Assessing financial education methods: Principles vs. rules-of-thumb approaches. *The Journal of Economic Education*, *47*(3), 193-210. | Did not meet intervention criteria |
| Skimmyhorn, W. (2016). Assessing financial education: Evidence from boot camp. *American Economic Journal: Economic Policy 8*(2), 322-343. | Did not meet design criteria |
| Smith-Brake, J. M. (2011). Enhancing financial capability among youth in Hochelaga-Maisonneuve (Québec Canada) dissertation, Southern New Hampshire University. | Did not meet design criteria |
| Smith, R. C., Sharp, E. H., & Campbell, R. (2011). Evaluation of financial fitness for life program and future outlook in the Mississippi Delta. *Journal of Economics and Economic Education Research*, *12*(2), 25-39. | Did not meet design criteria |
| Sommer, H. (2013). *Independent living service programs for foster youth: How individual factors and program features affect participation and outcomes*(Order No. 3593993). Retrieved from ProQuest Dissertations & Theses Global. (1441715980). | Did not meet intervention criteria |
| Ssewamala, F. M., & Sherraden, M. (2004). Saving for microenterprise in individual development accounts: Lessons from the American Dream Demonstration (CSD Report 04-18). St. Louis, MO: Washington University, Center for Social Development. | Did not meet design criteria |
| Ssewamala, F. M., Lombe, M., & Curley, J. C. (2006). Using individual development accounts for microenterprise development. *Journal of Developmental Entrepreneurship*, *11*(02), 117-131. | Did not meet design criteria |
| Ssewamala, F. M., & Sherraden, M. (2004). Integrating saving into microenterprise programs for the poor: Do institutions matter?. *Social Service Review*, *78*(3), 404-429. | Did not meet design criteria |
| Stegman, M. A., Faris, R., & Gonzalez, O. U. (2005). The impacts of IDA programs on family savings and asset holdings. Chapel Hill, NC: Center for Community Capitalism. | Did not meet design criteria |
| Stromski, L. & Holcomb, T. (2007). *United Way of King County: Individual Development Account* (Progress Report). Seattle, WA: United Way of King County. | Did not meet design criteria |
| Sun, J., Patel, F., Kirzner, R., Newton-Famous, N., Owens, C., Welles, S. L., & Chilton, M. (2016). The Building Wealth and Health Network: Methods and baseline characteristics from a randomized controlled trial for families with young children participating in temporary assistance for needy families (TANF). *BMC Public Health*, *16*(1), 583. | Did not meet outcomes criteria |
| Sykes, J., Elder, S., Gurbuzer, Y., & Principi, M. (2016). Exploring the linkages between youth financial inclusion and job creation: Evidence from the ILO school-to-work transition surveys (Work4Youth Publication Series No. 42). | Did not meet design criteria |
| Tang, N., & Peter, P. C. (2015). Financial knowledge acquisition among the young: The role of financial education, financial experience, and parents' financial experience. *Financial Services Review*, *24*(2), 119. | Did not meet design criteria |
| Taylor‐Carter, M. A., Cook, K., & Weinberg, C. (1997). Planning and expectations of the retirement experience. *Educational Gerontology: An International Quarterly*, *23*(3), 273-288 | Did not meet design criteria |
| Te’eni-Harari, T. (2016). Financial literacy among children: The role of involvement in saving money. *Young Consumers, 17*(2), 197-208.  Theodos, B., Stacy, C. P., & Daniels, R. (2018). Client led coaching: A random assignment evaluation of the impacts of financial coaching programs. *Journal of Economic Behavior & Organization*, *155*, 140-158. | Did not meet design criteria  Did not meet intervention criteria |
| Turnham, J. & Jefferson, A. (2012). *Pre-purchase counseling outcome study: Research brief.* U.S. Department of Housing and Urban Development. | Did not meet design criteria |
| Var, C. A. (2016). *Predictors of completion of financial behavior: Measuring self-efficacy, resilience, and financial mastery* (Doctoral dissertation, University of South Alabama).  Vosylis, R., & Klimstra, T. (2020). How does financial life shape emerging adulthood? Short-term longitudinal associations between perceived features of emerging adulthood, financial behaviors, and financial well-being. *Emerging Adulthood*, 1-19. [https://doi.org/10.1177/2167696820908970](https://doi.org/10.1177%2F2167696820908970)  Whitaker, E. A., Bokemeier, J. L., & Loveridge, S. (2011). Retirement plan participation in an era of change: The case of a rural region. *Rural Sociology, 76*(3), 319-346.  White, N. D., Packard, K. A., Flecky, K. A., Kalkowski, J. C., Furze, J. A., Ryan-Haddad, A. M., ... & Qi, Y. (2018). Two year sustainability of the effect of a financial education program on the health and wellbeing of single, low-income women. *Journal of Financial Counseling and Planning*, *29*(1), 68-74. | Did not meet design criteria  Did not meet OECD criteria  Did not meet design criteria  Did not meet intervention criteria |
| Wiedrich, K., Collins, J. M., Rosen, L., & Rademacher, I. (2014). *Financial education and account access among elementary students: Findings from the Assessing Financial Capability Outcomes (AFCO) youth pilot.* Washington, DC: Corporation for Enterprise Development (CFED). | Did not meet intervention criteria |
| Wilkinson, K. J. (2013). *Evaluation of the division of securities investor education seminars*(Order No. 1537236). Retrieved from ProQuest Dissertations & Theses Global. (1357148451). | Did not meet intervention criteria |
| Willett, M. (2008). A new model for retirement education and counseling. *Financial Services Review*, *17*(2). | Did not meet design criteria |
| Wolla, S. (2017). Evaluating the effectiveness of an online module for increasing financial literacy. *Social Studies Research and Practice*, *12*(2), 154-167.  Xiao, J. J., Sorhaindo, B., & Garman, E. T. (2006). Financial behaviours of consumers in credit counseling. *International Journal of Consumer Studies*, *30*(2), 108-121. | Did not meet design criteria  Did not meet design criteria |
| Xiao, J. J., & Wu, J. (2008). Completing debt management plans in credit counseling: An application of the theory of planned behavior. *Financial Counseling and Planning, 19*(2), 29-45.  Xu, X. (2018). Assessing a community-based financial literacy program: A case study in California’s Silicon Valley. *Journal of Financial Counseling and Planning*, *29*(1), 142-153. | Did not meet design criteria  Did not meet intervention criteria |
| Zhan, M. (2003). Savings outcomes of single mothers in Individual Development Accounts (CSD Working Paper 03-07). St. Louis, MO: Washington University, Center for Social Development. | Did not meet design criteria |
| Zhan, Min; Anderson, Steven; Scott, Jeff. (2009). Banking knowledge and attitudes of immigrants: Effects of a financial education program. *Social Development Issues, 31*(3), 15-32. | Did not meet design criteria |
| Zhan, M., & Grinstein-Weiss, M. (2007). Educational status and savings performance in Individual Development Accounts. *Journal of Policy Practice*, *6*(1), 27-46. | Did not meet design criteria |
| Zhan, M., Sherraden, M., & Schreiner, M. (2004). Welfare recipiency and savings outcomes in Individual Development Accounts. *Social Work Research*, *28*(3), 165-181.  Zhou, H., Dai, H., & Jung, N. (2020). Empowering migrant domestic helpers through financial education. *International Journal of Social Welfare*, *29*(2), 129-141. | Did not meet design criteria  Did not meet intervention criteria |

## Appendix E: Characteristics of Reports (63) that Reported on Studies that Met Inclusion Criteria

| **Study Name/Primary Reports** | **Secondary/**  **Duplicate Reports** | **Study Design** | **Larger Study** | **Intervention**  **(all include financial education)** | **Outcomes Studied** |
| --- | --- | --- | --- | --- | --- |
| **American Dream Demonstration Project**  Grinstein-Weiss, M., Lee, J., Greeson, J., Han, C., Yeo, Y., & Irish, K. (2008)  Grinstein-Weiss, M., Sherraden, M., Gale, W. G., Rohe, W. M., Schreiner, M., & Key, C. C. (2012)  Grinstein-Weiss, M., Sherraden, M., Gale, W. G., Rohe, W. M., Schreiner, M., & Key. C. C. (2013a)  Grinstein-Weiss, M., Sherraden, M., Gale, W. G., Rohe, W. M., Schreiner, M., Key. C. C. & Oliphant, J. E. (2015)  Han, C-K., Grinstein‐Weiss, M., & Sherraden, M. (2009)  Huang, J. (2010)  Huang, J., Lombe, M., Putnam, M., Grinstein-Weiss, M., & Sherraden, M. (2016)  Lombe, M. (2004)  Mills, G., Gale, W. G., Patterson, R., & Apotolov, E. (2006) | Grinstein-Weiss, M., Sherraden, M., Gale, W. G., Rohe, W., Schreiner, M. & Key, C. (2011)  Grinstein-Weiss, M., Sherraden, M., Gale, W. G., Rohe, W. M., Schreiner, M., & Key, C. C. (2013b)  Lombe, M. & Sherraden, M. (2008)  Mills, G., DeMarco, D., Patterson, R., Kaul, B., Rodger, C., Conjorni, J., & Tsen, W. (2003)  Mills, G., Patterson, R., Orr, L, & DeMarco, D. (2004)  Mills, G., Gale, W. G., Patterson, R. Engelhardt, G. V., Eriksen, M. D., & Apostolov, E. (2008) | RCT  RCT  RCT  RCT  RCT  RCT  RCT  RCT  RCT | American Dream Demonstration  American Dream Demonstration  American Dream Demonstration  American Dream Demonstration  American Dream Demonstration  American Dream Demonstration  American Dream Demonstration  American Dream Demonstration  American Dream Demonstration | Individual Development Accounts  Individual Development Accounts  Individual Development Accounts  Individual Development Accounts  Individual Development Accounts  Individual Development Accounts  Individual Development Accounts  Individual Development Accounts  Individual Development Accounts | Debt Amount  Asset Purchase – Homeownership  Debt Amount  Asset Purchase - Education  Asset Purchase – Home ownership  Saving Rate  Saving Amount  Asset Value  Asset Purchase  Debt Amount  Asset Value  Account Opening  Asset Purchase – Home Ownership  Asset Purchase – Business  Asset Value  Asset Purchase |
| **Assets for Independence**  Mills, G., McKernan, S-M., Ratcliffe, C., Edelstein, S., Pergamit, M., Braga, B., Hahn, H. & Elkin, S. (2016)  Ratcliffe, C., McKernan, S-M., Mills, G., Pergamit, M. & Braga, B. (2019) | Mills, G., McKernan, S-M., Ratcliffe, C., Edelstein, S., Pergamit, M. & Braga, B. (2019)  McKernan, S-M., Mills, G., Ratcliffe, C., Congdon, W. J., Pergamit, M., Braga, B, & Martinchek, K. (2020) | RCT  RCT | Assets for Independence  Assets for Independence | Individual Development Accounts  Individual Development Accounts | Saving Amount  Asset Purchase  Saving Amount  Asset Purchase |
| Collins, J. M., & Urban, C. (2016) |  | QED  (Author states RCT) | NA | Retirement Accounts | Account Opening  Retirement Savings  Retirement Savings Rate  Budget  Savings Amount |
| **Credit Building in IDA Programs**  Birkenmaier, J., Curley, J., & Kelly, P. (2012)  Birkenmaier, J., Curley, J., & Kelly, P. (2014a)  Birkenmaier, J., Curley, J., & Kelly, P. (2014b) |  | QED  QED  QED | Credit and IDAs  Credit and IDAs  Credit and IDAs | Individual Development Accounts  Individual Development Accounts  Individual Development Accounts | Credit Score  Credit Score  Credit Score |
| Duflo, E., Gale, W., Liebman, J., Orszag, P., & Saez, E. (2006) |  | RCT | NA | Retirement Accounts | Account Opening  Savings Amount |
| Goda, G., Manchester, C., & Sojourner, A. (2012) |  | RCT | NA | Retirement Accounts | Retirement Savings Retirement Savings Rate |
| Grinstein-Weiss, M., Cryder, C., Despard, M. R., Perantie, D. C., Oliphant, J. E. & Ariely, D. (2017a) |  | RCT | Refund to Savings | Tax Refund Savings/Investment | Savings Rate  Savings Amount |
| Grinstein-Weiss, M., Perantie, D. C., Russell, B. D., Comer, K., Taylor, S. H., Luo, L., Key, C., & Ariely, D. (2015) |  | RCT | Refund to Savings | Tax Refund Savings/Investment | Saving Amount |
| Grinstein-Weiss, M., Russell, B. D., Gale, W. G., Key, C., & Ariely, D. (2017b) |  | RCT | Refund to Savings | Tax Refund Savings/Investment | Saving Amount |
| Kim, J., Garman, E. T., & Sorhaindo, B. (2005) | Kim, Garman, E. & Sorhaindo, B. (2003) | QED | NA | Adult Financial Education Counseling and Coaching | Debt Amount  Budgeting |
| **Learn$ave IDA Project**  Leckie, N., Hui, T. S-W., Tattrie, D., Robson, J., & Voyer, J-P. (2010a) | Leckie, N., Hui, T. S-W., Tattrie, D., Robson, J., & Voyer, J-P. (2010b) | RCT | LearnSave | Individual Development Accounts | Saving Amount  Asset Value |
| Loke, V., Choi, L., Larin, L., & Libby, M. (2016) |  | QED | NA | Youth Bank Accounts | Saving Amount |
| Lusardi, A., Keller, P. A., & Keller, A. (2009) |  | QED | NA | Retirement Accounts | Account Opening |
| **Michigan SEED (MI SEED)**  Engelhardt, G. V., Dubnicki, A. L, Marks, E. L., & Rhodes, B. B. (2012)  Marks, E. L., Rhodes, B. B., Engelhardt, G. V., Scheffler, S., & Wallace, I. F. (2009) |  | RCT  RCT (Author describes as a QED) | Michigan SEED  Michigan SEED | Child Development Account  Child Development Account | Account Opening  Savings Amount  Budgeting  Savings Amount |
| Modestino, A. S. Sederberg, R, & Tuller, L. (2019) |  | RCT | NA | Adult Financial Education Counseling and Coaching | Credit Score |
| Moulton, S., Collins, J. M., Loibl, C. & Samek, A. (2015) |  | RCT | NA | Adult Financial Education Counseling and Coaching | Credit Score  Saving Amount |
| Osborne, C., Bobbitt, K., & Hovey, I. (2016) |  | QED | NA | Child Development Accounts | Account Opening  Saving Amount |
| **Parks Opportunity Program**  Collins, M. J. & Nafzinger, M. (2019)  Gons, N. (2013)  Weidrich, K., Gons, N., Collins, J.M. & Drever, A. (2014) | Collins, M. J., Gons, N. & Wiedrich, C. (2014) | RCT  RCT  RCT | Parks Opportunity Program  Parks Opportunity Program  Parks Opportunity Program | Adult Financial Education Counseling and Coaching  Adult Financial Education Counseling and Coaching  Adult Financial Education Counseling and Coaching | Credit Score  Debt  Savings Amount  Credit Score  Debt Amount  Credit Score  Debt Amount  Saving Amount |
| Roder, A. (2016) |  | QED | NA | Adult Financial Education Counseling and Coaching | Credit Score |
| Roll, S. P., Davison, G., Grinstein-Weiss, M., Despard, M. R., & Bufe, S. (2018) |  | RCT | Refund to Savings | Tax Refund Savings/Investment | Savings Amount |
| **SEED OK**  Beverly, S. G., Clancy, M. & Sherraden, M. (2014)  Beverly, S., Kim, Y., Sherraden, M., Nam, Y. & Clancy, M. (2015a)  Clancy, M. M., Beverly, S. G., Sherraden, M., & Huang, J. (2016)  Huang, J., Nam, Y., & Sherraden, M. S. (2013)  Huang, J., Nam, Y., Sherraden, M., & Clancy, M. (2015)  Huang, J., Kim, Y., Sherraden, M., & Clancy, M. (2017)  Huang, J., Beverly, S. G., Kim, Y., Clancy, M. M., & Sherraden, M. (2019)  Nam, Y., Kim, Y., Clancy, M., Zager, R., & Sherraden, M. (2013)  Wikoff, N., Huang, J., Kim, Y., & Sherraden, M. (2015) | Beverly, S. G., Clancy, M. Huang, J., & Sherraden, M. (2015b)  Mason, L. R., Nam, Y., Clancy, M. & Sherraden, M. (2013)  Sherraden, M., Clancy, M., Nam, Y., Huang, J., Kim, Y., Beverly, S. G., Mason, L. R., Wikoff, N E., Schreiner, M. & Parnell, J. Q. (2015)  Zager, R., Kim, Y., Nam, Y., Clancy, M., & Sherraden, M. (2010) | RCT  RCT  RCT  RCT  RCT  RCT  RCT  RCT  RCT | SEED OK  SEED OK  SEED OK  SEED OK  SEED OK  SEED OK  SEED OK  SEED OK  SEED OK | Child Development Accounts  Child Development Accounts  Child Development Accounts  Child Development Accounts  Child Development Accounts  Child Development Accounts  Child Development Accounts  Child Development Accounts  Child Development Accounts | Account Opening  Account Opening  Saving Amount  Account Opening  Saving Amount  Account Opening  Saving Amount  Asset Value  Account Opening  Saving Amount  Account Opening  Asset Value  Account Opening  Saving Amount  Account Opening |
| Smith, M. M., Hochberg, D., & Greene, W. H. (2017) |  | RCT | NA | Pre-purchase Homeownership Education and Mortgage Product | Credit Score  Debt Amount |
|  | Smith, M. M., Hochberg, D., & Greene, W. H. (2014) |  |  |  |  |
| Theodos, B., et al., (2016) |  | RCT | NA | Adult Financial Education Counseling and Coaching | Savings Amount  Credit Score  Debt Amount |
| Tufano, P. (2011) |  | RCT | NA | Tax-Time Saving/Investment | Saving Amount  Asset Purchase |

Individual Development Accounts = matched savings accounts with financial educatio
